# Anti-inflammatory butenolide derivatives from the coral-derived fungus *Aspergillus terreus* and structure revisions of aspernolides D and G, butyrolactone VI and 4′,8′′-diacetoxy butyrolactone VI†

**DOI:** 10.1039/c8ra01840e

**Published:** 2018-04-09

**Authors:** Mengting Liu, Qun Zhou, Jianping Wang, Junjun Liu, Changxing Qi, Yongji Lai, Hucheng Zhu, Yongbo Xue, Zhengxi Hu, Yonghui Zhang

**Affiliations:** Hubei Key Laboratory of Natural Medicinal Chemistry and Resource Evaluation, School of Pharmacy, Tongji Medical College, Huazhong University of Science and Technology Wuhan 430030 Hubei Province People's Republic of China zhangyh@mails.tjmu.edu.cn hzx616@126.com +86-27-83692762 +86-27-83692892; Department of Pharmacy, The Central Hospital of Wuhan Wuhan 430014 Hubei Province People's Republic of China

## Abstract

Chemical investigation of the coral-derived fungus *Aspergillus terreus* led to the discovery of ten butenolide derivatives (1–10), including four new ones (1–4). The new structures were characterized on the basis of comprehensive spectroscopic analysis, including 1D and 2D NMR and HRESIMS data. Compounds 1 and 2 were a pair of rare C-8′′ epimers with vicinal diol motifs. The absolute configurations of 1–4 were determined *via* [Mo_2_(AcO)_4_] induced circular dichroism (ICD) spectra and comparison of their experimental ECD spectra. Importantly, the structures of reported aspernolides D and G, butyrolactone VI and 4′,8′′-diacetoxy butyrolactone VI have been correspondingly revised *via* a combined strategy of experimental validations, ^13^C NMR predictions by ACD/Labs software, and ^13^C NMR calculations. Herein we provide valuable referenced ^13^C NMR data (C-7′′, C-8′′, and C-9′′) for the structure elucidations of butenolide derivatives with 1-(2-hydroxyphenyl)-3-methylbutane-2,3-diol, 2-(2,3-dihydrobenzofuran-2-yl)propan-2-ol, or 2,2-dimethylchroman-3-ol motifs. Additionally, all the isolates (1–10) were assessed for anti-inflammatory activity by measuring the amount of NO production in lipopolysaccharide (LPS)-induced RAW 264.7 mouse macrophages, and compound 10 showed an even stronger inhibitory effect than the postive control indomethacin, presenting it as a promising lead compound for the development of new anti-inflammatory agents.

## Abstract 2

Chemical investigation of the coral-derived fungus *Aspergillus terreus* led to the discovery of ten butenolide derivatives (1–10), including four new ones (1–4).

## Graphical abstract

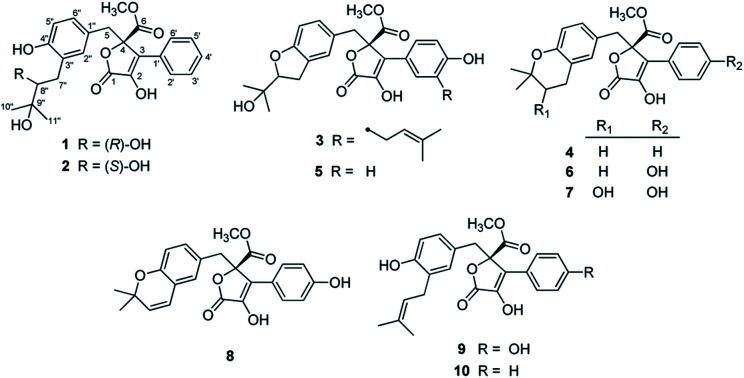

## Introduction

Microorganisms have been regarded as an under-explored source of structurally interesting and bioactive natural products with the potential to provide attractive lead compounds for drug discovery.^1^ As one of the most useful fungi, the *Aspergillus* genus was found to have powerful clusters to biosynthesize plenty of complex secondary metabolites,^2^ including lignans, alkaloids, terpenes, polyketides, peptides, *etc.*, showing intriguing pharmaceutical activities, upon which some ground-breaking research has been finished by our research group. For example, *Aspergillus flavipes* produces several bioactive merocytochalasans (anti-tumor agents), namely asperchalasine A,^3^ epicochalasines A and B,^4^ asperflavipine A,^5^ and aspergilasines A–D,^6^ which were characterized by architecturally complex polycyclic rings and multiple chiral centers; *Aspergillus terreus* produces two unprecedented meroterpenoids, namely asperterpenes A and B,^7^ showing potent BACE1 inhibitory activities for Alzheimer's disease treatment; *Aspergillus* sp. TJ23 produces a bridged spirocyclic meroterpenoid, namely spiroaspertrione A,^8^ which was found to be a PBP2a inhibitor and act as a potent potentiator of oxacillin against methicillin-resistant *Staphylococcus aureus*. Inspired by these structurally unexpected natural products with tempting pharmacological activities from the *Aspergillus* genus, we are devoted to the investigation of *Aspergillus* species from different origins for chemical and pharmacological diversity.

**Fig. 1:**
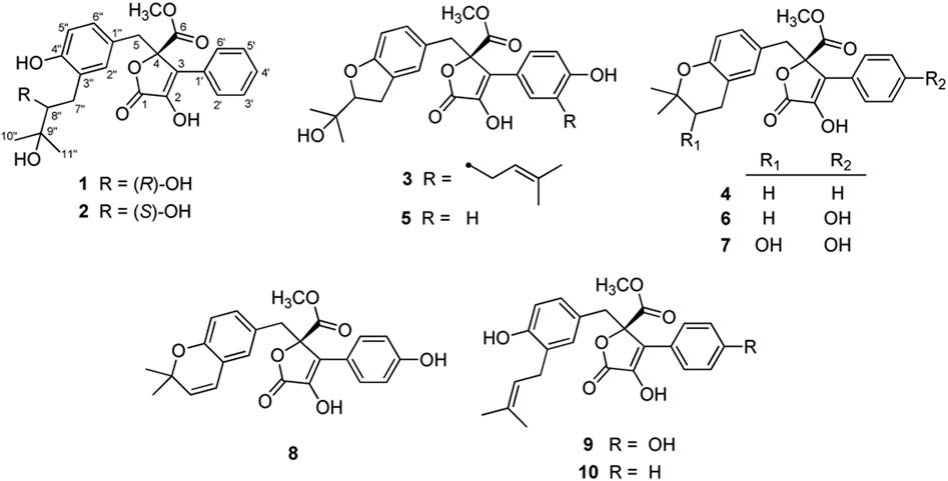
Chemical structures of compounds 1–10.

In our efforts to explore bioactive natural products from marine-derived fungi,^9^ we performed a chemical investigation on the fermented rice substrate of a coral-derived fungus *Aspergillus terreus*, resulting in the isolation of ten butenolide derivatives (1–10), including four new ones (1–4), wherein 1 and 2 were a pair of rare C-8′′ epimers with vicinal diol motifs. Importantly, the NMR data of 5 and 7 were closely similar to those of reported aspernolide D^10^ and butyrolactone VI,^11^ which inspired us to perform the structure reassignments of reported aspernolides D and G, butyrolactone VI and 4′,8′′-diacetoxy butyrolactone VI, as assisted by a combined strategy of experimental validations, ^13^C NMR predictions by ACD/Labs software, and ^13^C NMR calculations. Herein, we report the isolation, structure elucidation, structure reassignments, and anti-inflammatory activity of these butenolide derivatives (Fig. 1).

## Results and discussion

Compounds 1 and 2, both obtained as white, amorphous powders, were identified to have the same molecular formula C_24_H_26_O_8_, according to their HRESIMS and ^13^C NMR data, indicative of twelve indices of hydrogen deficiency. The close resemblances of 1D and 2D NMR data (Table 1) of 1 and 2 indicated that both compounds were a pair of epimers. The IR spectrum of 1 showed broad and intense absorption bands for hydroxy (3433 cm^−1^), ester/lactone carbonyl (1743 cm^−1^) and aromatic rings (1617, 1501, 1438 cm^−1^). In the ^1^H NMR data of 1, a 1,2,4-trisubstituted benzene motif was observed based on the ABX system for three aromatic protons (*δ*_H_ 6.70, s, H-2′′; 6.52, d, *J* = 8.1 Hz, H-5′′; 6.29, d, *J* = 8.1 Hz, H-6′′). Additionally, the 1D NMR data also showed the signals of *δ*_H_ 7.66 (d, *J* = 7.7 Hz, H-2′, 6′)/*δ*_C_ 127.7 (C-2′, 6′), *δ*_H_ 7.40 (dd, *J* = 7.3, 7.7 Hz, H-3′, 5′)/*δ*_C_ 129.1 (C-3′, 5′), and *δ*_H_ 7.35 (dd, *J* = 7.3, 7.3 Hz, H-4′)/*δ*_C_ 129.2 (C-4′), indicating the presence of a mono-substituted benzene motif. These characteristic data suggested that compound 1 was a butenolide derivative.

**Table 1:** ^1^H and ^13^C NMR data for compounds 1–4 (*δ* in ppm and *J* in Hz)

No.	1		2		3		4	
	*δ*_H_ a ^,^ b ^,^ d	*δ*_C_ c ^,^ d	*δ*_H_ a ^,^ b ^,^ d	*δ*_C_ c ^,^ d	*δ*_H_ a ^,^ b ^,^ e	*δ*_C_ c ^,^ e	*δ*_H_ a ^,^ b ^,^ e	*δ*_C_ c ^,^ e
1	—	170.0 C	—	170.1 C	—	171.8 C	—	170.0 C
2	—	140.3 C	—	140.4 C	—	140.4 C	—	142.1 C
3	—	127.8 C	—	127.5 C	—	129.5 C	—	128.6 C
4	—	86.2 C	—	86.2 C	—	87.0 C	—	86.9 C
5	3.50 m	38.7 CH_2_	3.51 d (4.2)	38.6 CH_2_	3.44 d (9.1)	39.7 CH_2_	3.42 d (1.6)	39.4 CH_2_
6	—	170.0 C	—	170.1 C	—	171.8 C	—	171.4 C
6-OMe	3.76 s	53.8 CH_3_	3.78 s	53.8 CH_3_	3.77 s	53.8 CH_3_	3.76 s	53.9 CH_3_
1′	—	130.2 C	—	130.4 C	—	125.2 C	—	131.9 C
2′	7.66 d (7.7)	127.7 CH	7.71 d (7.8)	127.5 CH	6.45 d (2.1)	132.5 CH	7.64 d (7.4)	128.6 CH
3′	7.40 dd (7.3, 7.7)	129.1 CH	7.42 dd (7.1, 7.8)	129.2 CH	—	128.4 C	7.43 dd (7.3, 7.4)	129.8 CH
4′	7.35 dd (7.3, 7.3)	129.2 CH	7.33 dd (7.1, 7.1)	129.1 CH	—	155.1 C	7.35 dd (7.3, 7.3)	129.7 CH
5′	7.40 dd (7.3, 7.7)	129.1 CH	7.42 dd (7.1, 7.8)	129.2 CH	6.48 d (8.1)	115.1 CH	7.43 dd (7.3, 7.4)	129.8 CH
6′	7.66 d (7.7)	127.7 CH	7.71 d (7.8)	127.5 CH	6.53 dd (2.1, 8.1)	129.8 CH	7.64 d (7.4)	128.6 CH
7′	—	—	—	—	3.08 br d (7.4)	28.8 CH_2_	—	—
8′	—	—	—	—	5.09 m	123.8 CH	—	—
9′	—	—	—	—	—	132.8 C	—	—
10′	—	—	—	—	1.67 s	26.0 CH_3_	—	—
11′	—	—	—	—	1.59 s	17.8 CH_3_	—	—
1′′	—	124.9 C	—	124.7 C	—	124.7 C	—	125.4 C
2′′	6.70 s	133.1 CH	6.35 s	133.2 CH	7.68 s	125.6 CH	6.41 d (2.1)	132.6 CH
3′′	—	126.2 C	—	125.6 C	—	129.5 C	—	121.4 C
4′′	—	154.5 C	—	154.5 C	—	161.6 C	—	154.3 C
5′′	6.52 d (8.1)	116.5 CH	6.62 d (8.2)	116.5 CH	6.83 d (8.5)	110.1 CH	6.38 d (8.3)	117.4 CH
6′′	6.29 d (8.1)	130.1 CH	6.64 d (8.2)	130.7 CH	7.43 d (8.5)	128.7 CH	6.45 dd (2.1, 8.3)	130.2 CH
7′′	2.43 br d (14.0); 2.69 dd (10.2, 14.0)	33.7 CH_2_	2.33 br d (14.0); 2.61 dd (10.2, 14.0)	33.9 CH_2_	3.23 m	31.4 CH_2_	2.51 m	23.2 CH_2_
8′′	3.48 m	81.2 CH	3.43 m	80.9 CH	4.66 dd (8.3, 9.5)	91.0 CH	1.67 t (6.8)	33.7 CH_2_
9′′	—	74.0 C	—	73.9 C	—	72.5 C	—	75.1 C
10′′	1.14 s	22.5 CH_3_	1.14 s	23.0 CH_3_	1.28 s	25.1 CH_3_	1.20 s	27.0 CH_3_
11′′	1.21 s	26.2 CH_3_	1.19 s	26.3 CH_3_	1.25 s	25.4 CH_3_	1.20 s	27.1 CH_3_

a Recorded at 400 MHz.

b “m” means overlapped or multiplet with other signals.

c Recorded at 100 MHz.

d Recorded in CDCl_3_.

e Recorded in methanol-*d*_4_.

**Fig. 2:**
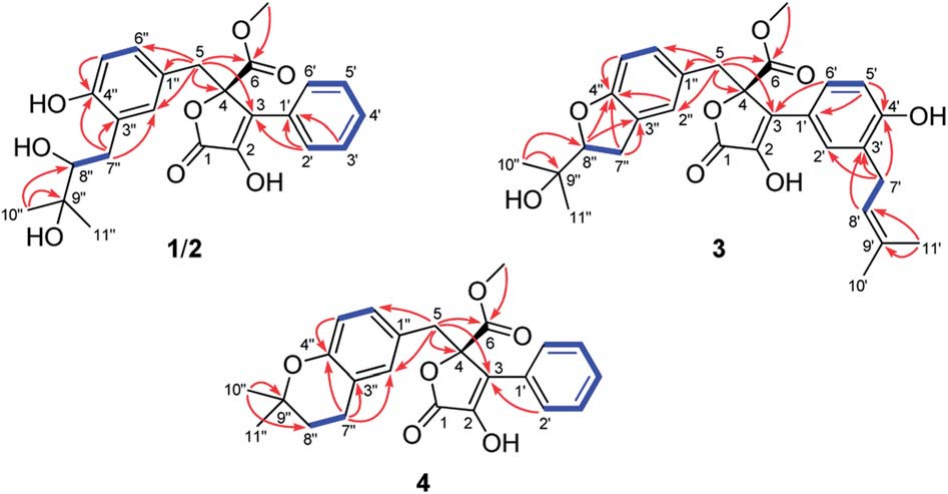
Selected ^1^H–^1^H COSY (blue lines) and HMBC (red arrows) correlations of compounds 1–4.

Detailed analysis of the 1D and 2D NMR data of 1 implied that its structural features were closely related to those of the known compound versicolactone B (10),^12^ whose absolute structure was confirmed by single-crystal X-ray diffraction analysis, with the only difference that a Δ^8′′,9′′^ double bond in 10 was replaced by an oxygenated methine carbon (*δ*_C_ 81.2, C-8′′) and an oxygenated tertiary carbon (*δ*_C_ 74.0, C-9′′) in 1, as supported *via* the molecular formula C_24_H_26_O_8_ required by its HRESIMS data and the HMBC correlations from H_3_-10′′ to C-8′′ and C-9′′. The gross structures of 1 and 2 were further defined as shown *via* 2D NMR analysis, including ^1^H–^1^H COSY and HMBC spectral data (Fig. 2).

**Fig. 3:**
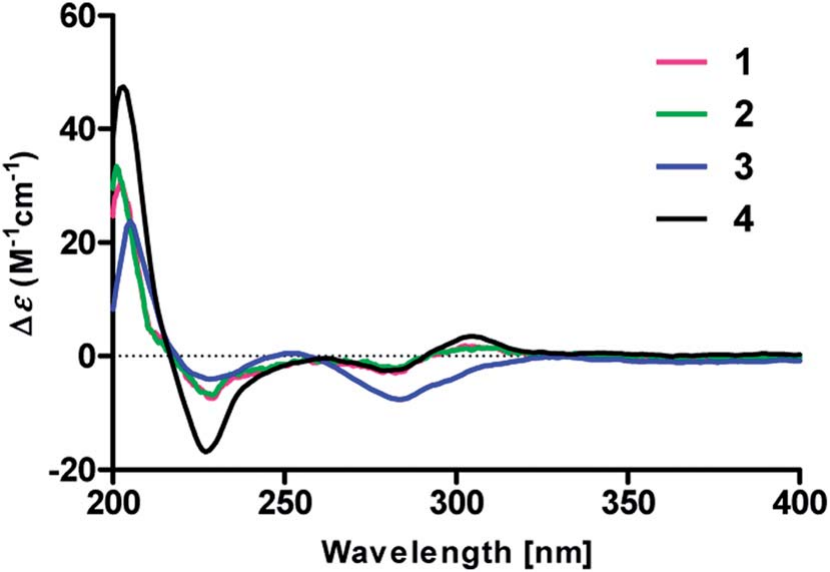
Experimental ECD spectra of compounds 1–4 in MeOH.

To determine the absolute configurations, the experimental ECD spectra of compounds 1 and 2 were measured in MeOH (Fig. 3), which were identical to that of versicolactone B,^12^ showing positive Cotton effects at approximately 203 and 307 nm and a negative Cotton effect at approximately 230 nm that were ascribed to the conjugated functionality of an α,β-unsaturated carboxylic ester motif linked to a benzene group. Thus, the C-4 in 1 and 2 were both defined to be *R*-configurations. Accordingly, compounds 1 and 2 should be a pair of C-8′′ epimers.

**Fig. 4:**
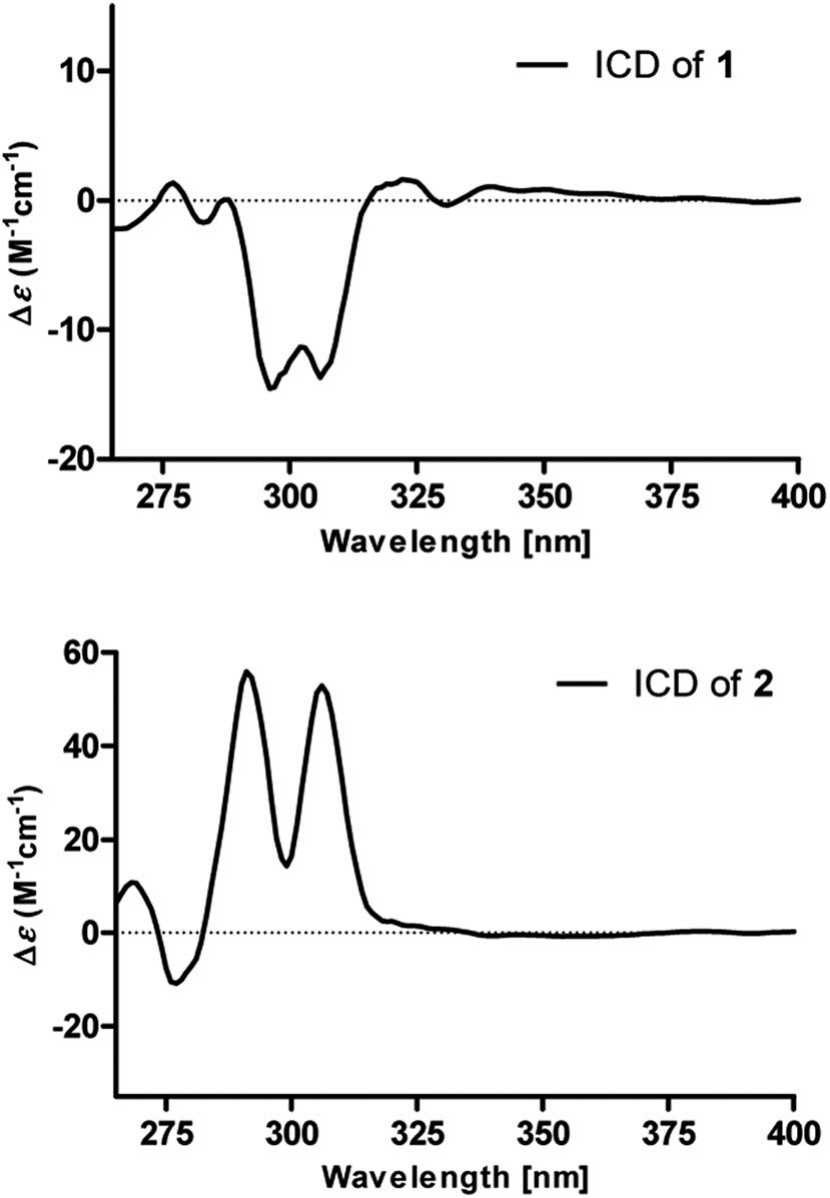
[Mo_2_(OAc)_4_] induced ICD spectra of 1 and 2 in DMSO.

The absolute configurations of 8′′,9′′-diol motifs in 1 and 2 were determined on the basic of *in situ* dimolybdenum CD method.^13^ Compound 1 was mixed with Mo_2_(AcO)_4_ in DMSO to provide a metal complex, which showed a negative Cotton effect at approximately 305 nm (Fig. 4), permitting assignment of the 8′′*R*-configuration for 1, according to the empirical helicity rule relating the Cotton effect sign of the diagnostic O–C–C–O moiety.^13^ Just using the same method like 1, compound 2 showed a positive Cotton effect at approximately 305 nm (Fig. 4), thus suggesting the 8′′*S*-configuration for 2.^14^ Therefore, the absolute structures of 1 and 2 were defined and named 8′′*R*,9′′-diol versicolactone B and 8′′*S*,9′′-diol versicolactone B, respectively.

Compound 3 was obtained as a white, amorphous powder. The HRESIMS data showed a sodium adduct ion at *m*/*z* 531.1986 [M + Na]^+^ (calcd for C_29_H_32_O_8_Na, 531.1995), indicating a molecular formula of C_29_H_32_O_8_. A direct comparison of its 1D NMR data (Table 1) with those of 5 indicated that a 1,4-disubstituted benzene motif in 5 was replaced by a 1,2,4-trisubstituted benzene group in 3 with an isopentene group positioned at C-3′, as supported by the ^1^H–^1^H COSY correlation of H_2_-7′ and H-8′ and HMBC correlations from H_3_-10′ and H_3_-11′ to C-8′ and C-9′ and from H_2_-7′ and H-8′ to C-3′ (*δ*_C_ 128.4) (Fig. 2). Moreover, the experimental ECD spectrum of 3 was related to those of 1 and 2 (Fig. 3), suggesting a 4*R*-configuration for 3. Hence, the structure of 3 was defined and named 3′-isoamylene butyrolactone IV.

Compound 4, also purified as a white, amorphous powder, was assigned the molecular formula C_24_H_24_O_6_ based on HRESIMS data at *m*/*z* 431.1464 [M + Na]^+^ (calcd for C_24_H_24_O_6_Na, 431.1471). The ^1^H and ^13^C NMR data of 4 (Table 1) were similar to those of 6, with the only difference being that a 1,4-disubstituted benzene motif in 6 was replaced by a mono-substituted benzene group linked to C-3 in 4, as supported *via* the ^1^H–^1^H COSY correlations of H-2′/H-3′/H-4′/H-5′/H-6′ and HMBC correlation from H-2′ to C-3 (Fig. 2). Furthermore, the experimental ECD spectrum (Fig. 3) of 4 coincided well with those of 1 and 2, suggesting that a 4*R*-configuration should also exist for 4. Hence, the absolute structure of 4 was defined and named 4′-dehydroxy aspernolide A.

The six known butenolide derivatives were identified as butyrolactone IV (5),^15^ aspernolide A (6),^16^ butyrolactone V (7),^17^ aspernolide E (8),^18^ butyrolactone I (9),^12^ and versicolactone B (10)^12^ by comparison of their spectroscopic data with those reported in the literature.

**Fig. 5:**
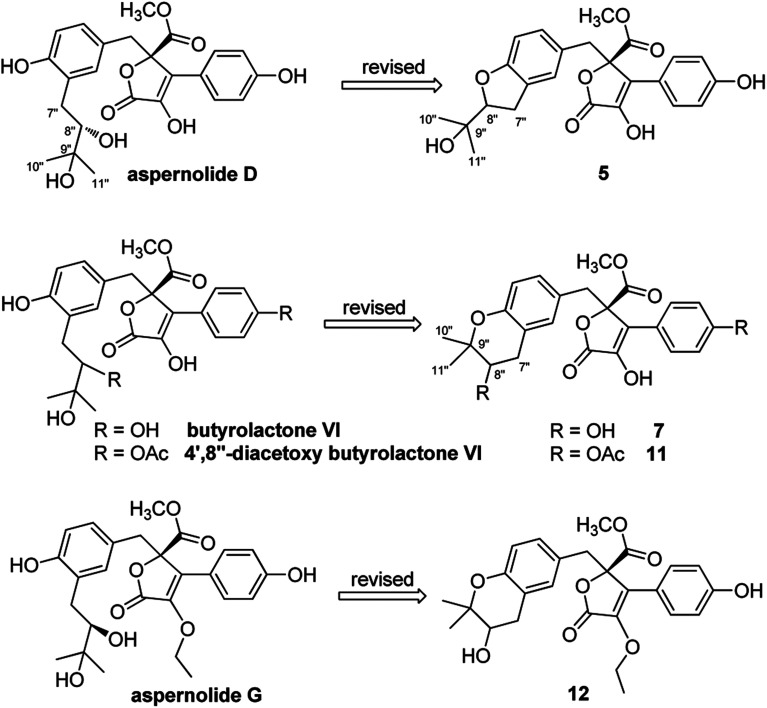
Structure revisions of aspernolides D and G, butyrolactone VI and 4′,8′′-diacetoxy butyrolactone VI.

**Fig. 6:**
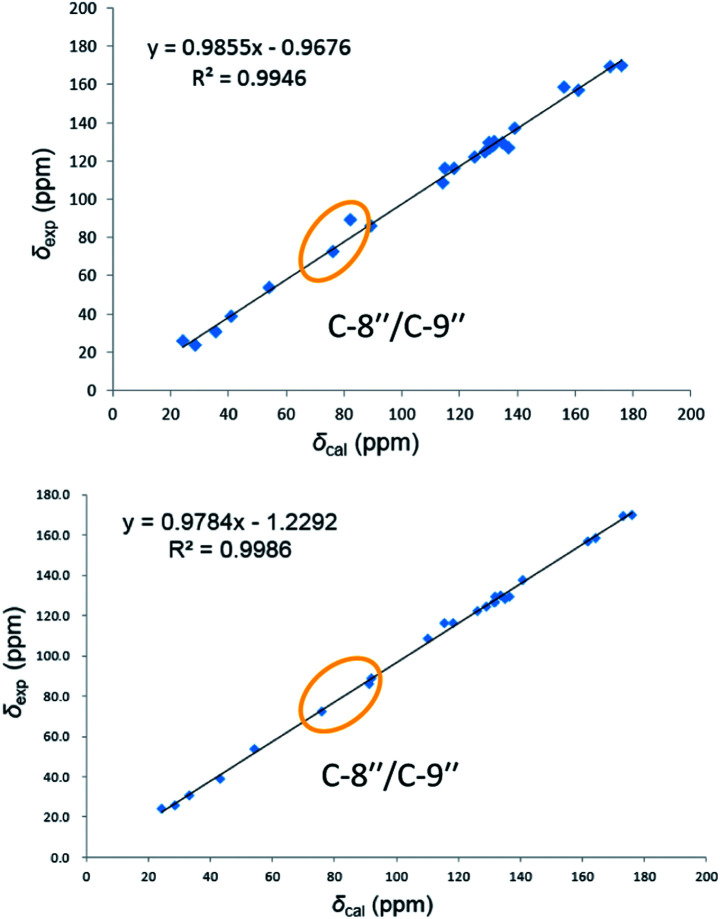
Linear correlations between the calculated and experimental ^13^C NMR chemical shifts for aspernolide D (up) and 5 (down).

**Fig. 7:**
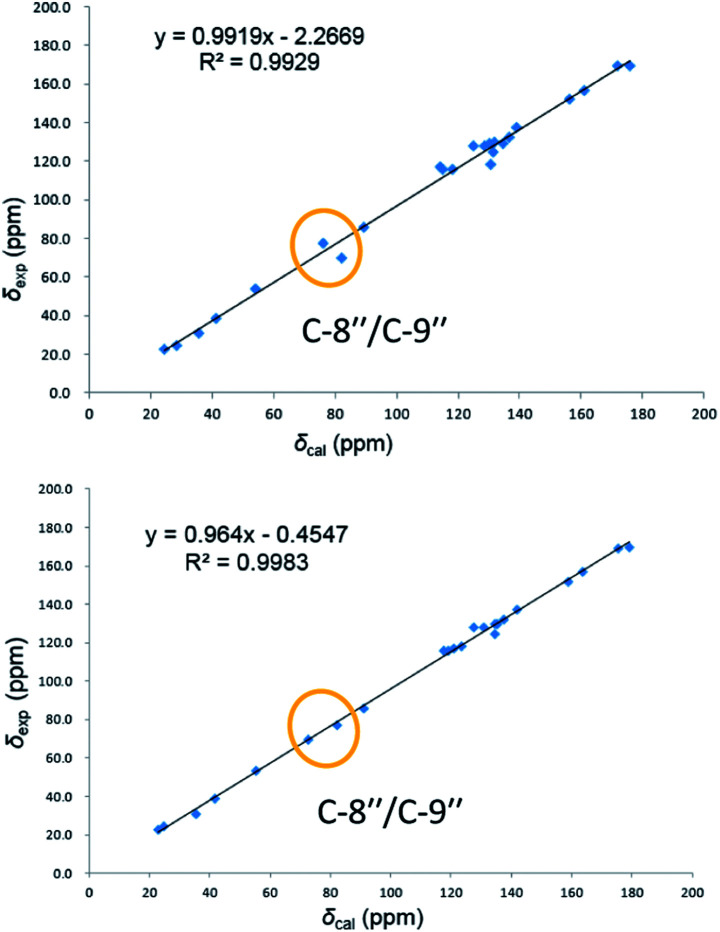
Linear correlations between the calculated and experimental ^13^C NMR chemical shifts for butyrolactone VI (up) and 7 (down).

On reviewing the literature, the pivotal ^13^C NMR data for aspernolide D^10^ [*δ*_C_ 30.5 (CH_2_, C-7′′) 89.1 (CH, C-8′′), and 72.4 (C, C-9′′)] and butyrolactone VI^11^ [*δ*_C_ 31.0 (CH_2_, C-7′′) 69.6 (CH, C-8′′), and 77.2 (C, C-9′′)] showed close resemblances to those of compounds 5 and 7 (Table 2), respectively, which inspired us to investigate the regular ^13^C NMR data of C-7′′, C-8′′, and C-9′′ in the 1-(2-hydroxyphenyl)-3-methylbutane-2,3-diol, 2-(2,3-dihydrobenzofuran-2-yl)propan-2-ol, and 2,2-dimethylchroman-3-ol motifs for the butenolide derivatives. Take 1, 5, and 7 for examples (Table 2), their chemical shifts at C-7′′ showed no obviously diagnostic differences; however, the chemical shifts at C-8′′ and C-9′′ showed apparent differences [*δ*_C_ 81.2 (C-8′′) and 74.0 (C-9′′) for 1; *δ*_C_ 90.4 (C-8′′) and 72.5 (C-9′′) for 5; *δ*_C_ 70.4 (C-8′′) and 78.0 (C-9′′) for 7], corresponding to the predicted ^13^C NMR data *via* “C + H NMR Predictor and DB” within the ACD/Labs software suite, which was regarded as a powerful and useful tool to predict the chemical shifts of a given input structure and resolve constitutional structure revisions.^19^ The above-mentioned results indicated that aspernolide D and butyrolactone VI should be revised to 5 and 7 (Fig. 5), respectively, as supported by the calculations of ^13^C NMR chemical shifts with two sets of *R*^2^ values: 0.9946 for aspernolide D and 0.9986 for 5 (Fig. 6); 0.9929 for butyrolactone VI and 0.9983 for 7 (Fig. 7). Accordingly, the acetylated product of butyrolactone VI [*δ*_C_ 28.1 (CH_2_, C-7′′) 70.9 (CH, C-8′′), and 75.1 (C, C-9′′)],^11^ named 4′,8′′-diacetoxy butyrolactone VI, was also revised to 11 (Fig. 5) with an identical 2,2-dimethylchroman-3-ol motif. In addition, the ^13^C NMR data [*δ*_C_ 31.4 (CH_2_, C-7′′) 68.4 (CH, C-8′′), and 77.4 (C, C-9′′)] of aspernolide G^20^ were very consistent with those of 7 [*δ*_C_ 32.0 (CH_2_, C-7′′) 70.4 (CH, C-8′′), and 78.0 (C, C-9′′)] (Table 2), indicating that aspernolide G should be revised to 12 (Fig. 5). Remarkably, our current work provide a valuable referenced ^13^C NMR data (C-7′′, C-8′′, and C-9′′) for structure elucidations of the butenolide derivatives with planar 1-(2-hydroxyphenyl)-3-methylbutane-2,3-diol, 2-(2,3-dihydrobenzofuran-2-yl)propan-2-ol, or 2,2-dimethylchroman-3-ol motifs. However, for the determination of absolute configuration of C-8′′, maybe some reliable methods, including Mosher's technique, [Rh_2_(OCOCF_3_)_4_] induced circular dichroism (ICD) spectra, X-ray diffraction crystallography, *etc.*, were best to be used for these compounds.

**Table 2:** Comparison of chemical shifts of 1, 5, and 7 at C-7′′, C-8′′, and C-9′′, respectively, *via* experimental validations, ^13^C NMR predictions by ACD/Labs software, and ^13^C NMR calculations (*δ* in ppm, in CDCl_3_)

Compd no.	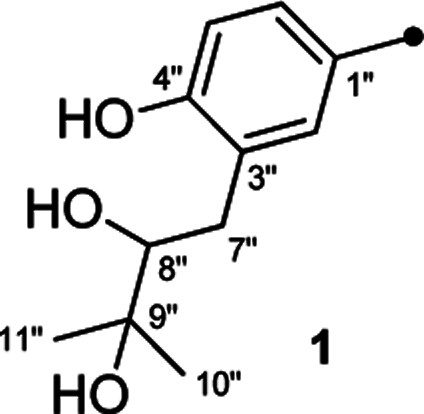	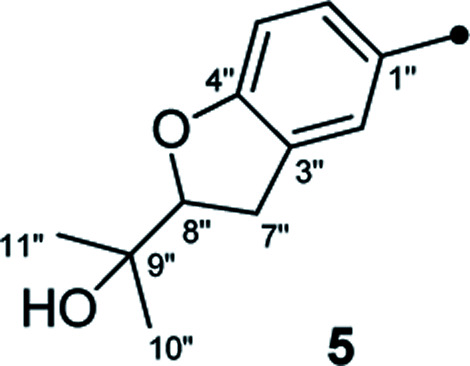	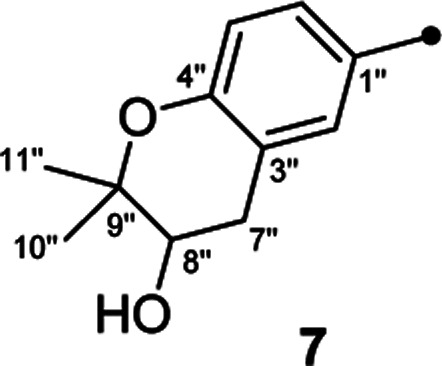
	1-(2-Hydroxyphenyl)-3-methylbutane-2,3-diol motif	2-(2,3-Dihydrobenzofuran-2-yl)propan-2-ol motif	2,2-Dimethylchroman-3-ol motif
**Exptl chemical shifts**			
7′′	33.7	31.4	32.0
8′′	81.2	90.4	70.4
9′′	74.0	72.5	78.0

**C + H NMR predictors and DB in ACD/Labs**			
7′′	32.0	30.1	30.8
8′′	79.1	88.8	69.9
9′′	73.5	71.9	77.5

**Calcd chemical shifts**			
7′′	32.2	29.9	31.8
8′′	76.0	85.3	67.1
9′′	70.3	70.0	76.1

**Fig. 8:**
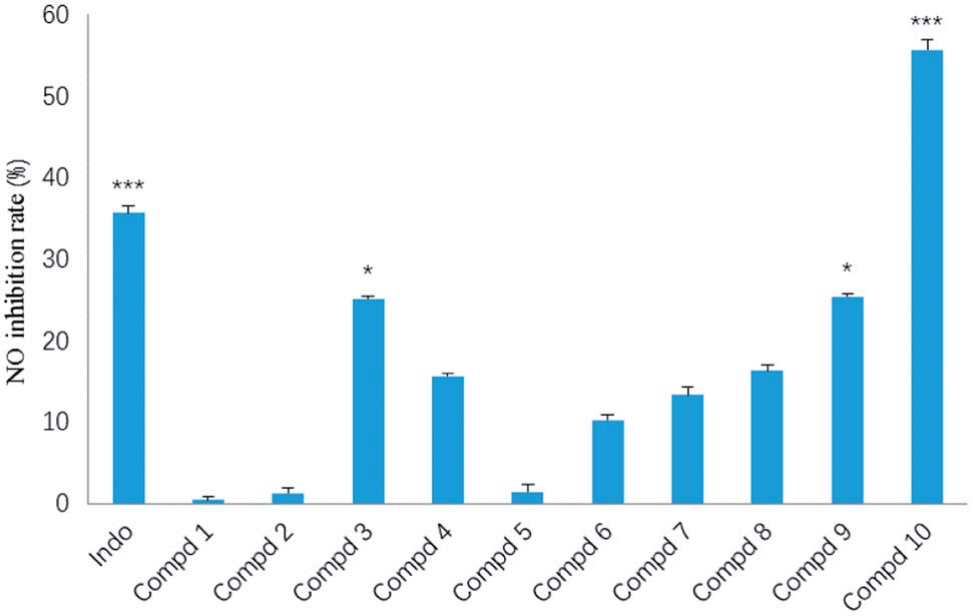
Inhibitory effect of compounds 1–10 against NO production in LPS-stimulated RAW264.7 cells. The results are expressed as the mean ± SD from three independent experiments. *p < 0.05, **p < 0.01, ***p < 0.001 as compared to LPS group.

In our screening of anti-inflammatory agents from natural products,^21^ all the isolates (1–10) were evaluated for inhibitory effects against NO production in RAW264.7 mouse macrophages induced by lipopolysaccharide (LPS) at a concentration of 20 μM, with indomethacin (50 μM) as the positive control. Among them (Fig. 8), the inhibitory effect of compound 10 (***p < 0.001) was even stronger than that of indomethacin. Additionally, compounds 3 and 9 also exerted modest inhibitory effect (*p < 0.05) on NO production with inhibition ratios of nearly 25.1% and 25.3%, respectively. The remaining seven compounds (1, 2 and 4–8) were inactive against NO production.

## Conclusions

In conclusion, ten butenolide derivatives (1–10), including four new ones (1–4), were isolated from the coral-derived fungus *Aspergillus terreus*. Remarkably, compounds 1 and 2 were a pair of rare C-8′′ epimers with vicinal diol motifs, and the absolute configurations of 1–4 were determined *via* [Mo_2_(AcO)_4_] induced circular dichroism (ICD) spectra and comparison of their experimental ECD spectra. Importantly, the structures of reported aspernolides D and G, butyrolactone VI and 4′,8′′-diacetoxy butyrolactone VI have been correspondingly revised *via* a combined strategy of experimental validations, ^13^C NMR predictions by ACD/Labs software, and ^13^C NMR calculations. Remarkably, compounds 3, 9 and 10 showed remarkable inhibitory effects against NO production, of which compound 10, was even stronger than that of indomethacin (a positive control), endowing 10 as a promising lead compound for the development of new anti-inflammatory agents. Our findings in this report not only enrich our knowledge about the chemical and pharmacological diversities of butenolide derivatives in the *Aspergillus* genus, but also provide a valuable referenced ^13^C NMR data (C-7′′, C-8′′, and C-9′′) for structure elucidations of the butenolide derivatives with 1-(2-hydroxyphenyl)-3-methylbutane-2,3-diol, 2-(2,3-dihydrobenzofuran-2-yl)propan-2-ol, or 2,2-dimethylchroman-3-ol motifs.

## Experimental section

### General experimental procedures

Optical rotations were recorded using a PerkinElmer PE-341 instrument (PerkinElmer, Waltham, MA, USA). UV spectra were recorded with a Varian Cary 50 UV/vis spectrophotometer (Varian, Salt Lake City, UT, USA). IR spectra were measured with a Bruker Vertex 70 FT-IR spectrophotometer (Bruker, Karlsruhe, Germany) with KBr pellets. ECD data were collected with a JASCO-810 spectrometer. 1D and 2D NMR spectra were recorded with a Bruker AM-400 NMR spectrometer (Bruker, Karlsruhe, Germany) using TMS as internal standard. All chemical shifts (*δ*) were expressed in ppm with reference to the solvent signals for CDCl_3_ (*δ*_H_ 7.24 and *δ*_C_ 77.23) or methanol-*d*_4_ (*δ*_H_ 3.31 and *δ*_C_ 49.0). High-resolution electrospray ionization mass spectrometry (HRESIMS) data were recorded with a Thermo Fisher LTQ XL LC/MS (Thermo Fisher, Palo Alto, CA, USA), by calibrating the instrument with aqueous sodium trifluoroacetate solution and then dissolving and infusing the samples with eluent CH_3_CN–H_2_O (1 : 1, v/v). Semi-preparative HPLC was performed on an Agilent 1200 liquid chromatograph with Zorbax SB-C_18_ (9.4 mm × 250 mm) column. Silica gel (200–300 mesh, Qingdao Marine Chemical, Inc., Qingdao, People's Republic of China) and Lichroprep RP-C_18_ gel (40–63 μm, Merck, Darmstadt, Germany) were performed for column chromatography (CC). Precoated TLC plates (200–250 μm thickness, silica gel 60 F_254_, Qingdao Marine Chemical, Inc.) was performed for thin-layer chromatography. Fractions were monitored by TLC and spots were visualized by heating silica gel plates sprayed with 10% H_2_SO_4_ in EtOH.

### Fungus material

The fungal strain *Aspergillus terreus* was isolated from a piece of tissue from the inner part of the soft coral *Sarcophyton subviride* collected from the Xisha Island (16°45′N, 111°65′E) in the South China Sea in October 2016. It was identified by one of the authors (J. Wang) according to its morphology and sequence analysis of the ITS region of the rDNA (GenBank accession no. MF972904). The strain has been deposited in the culture collection of Tongji Medical College, Huazhong University of Science and Technology.

### Fermentation, extraction, and isolation

The fungal strain *Aspergillus terreus* was grown on PDA medium at 28 °C for 7 days, which was inoculated statically in 300 × 500 mL Erlenmeyer flasks (each containing 200 g rice and 200 mL water) for 28 days. The whole rice solid medium was extracted seven times in 95% aqueous EtOH at room temperature, and the solvent was concentrated under reduced pressure to afford a total residue, which was then suspended in water and partitioned successfully with EtOAc. The EtOAc extract (1.5 kg) was subjected to silica gel CC eluted with a stepwise gradient of petroleum ether–ethyl acetate–MeOH (10 : 1 : 0, 7 : 1 : 0, 5 : 1 : 0, 3 : 1 : 0, 1 : 1 : 0, 2 : 2 : 1, 1 : 1 : 1) to yield seven fractions (A–G).

Fraction C (75 g) was subjected to an RP-C_18_ column eluted with MeOH–H_2_O (from 20 : 80 to 100 : 0, v/v) to afford five fractions (C1–C5). Fraction C3 (2.3 g) was repeatedly separated *via* Sephadex LH-20 eluted with CH_2_Cl_2_–MeOH (1 : 1, v/v), and followed by silica gel CC (stepwise petroleum ether–ethyl acetate, 4 : 1–1 : 1) and semi-preparative HPLC using MeOH–H_2_O (60 : 40, v/v, 2.0 mL min^−1^), to yield compounds 6 (22.3 mg; *t*_R_ 31.5 min), 8 (11.1 mg; *t*_R_ 28.2 min) and 9 (36.1 mg; *t*_R_ 23.5 min). Fraction C4 (320.5 mg) was purified by semi-preparative HPLC (MeOH–H_2_O, 65 : 35, v/v, 3.0 mL min^−1^) to give compounds 4 (27.6 mg; *t*_R_ 28.4 min) and 10 (20.1 mg; *t*_R_ 24.6 min).

Fraction D (198 g) was separated by an RP-C_18_ column with MeOH–H_2_O (from 20 : 80 to 100 : 0, v/v) as eluent to yield five fractions (D1–D5). Fraction D3 (42 g) was separated through Sephadex LH-20 eluted with CH_2_Cl_2_–MeOH (1 : 1, v/v) and RP-C_18_ column with MeOH–H_2_O (from 20 : 80 to 80 : 20, v/v), and followed by semi-preparative HPLC using CH_3_CN–H_2_O (60 : 40, v/v, 3.0 mL min^−1^) to yield compound 3 (13.4 mg; *t*_R_ 23.8 min).

Fraction E (186 g) was chromatographed on silica gel CC (CH_2_Cl_2_–MeOH, 1 : 0–50 : 1, v/v) to yield five main fractions (E1–E5). Fraction E4 (4.6 g) was applied to Sephadex LH-20 using CH_2_Cl_2_–MeOH (1 : 1, v/v), and followed by semi-preparative HPLC using CH_3_CN–H_2_O (55 : 45, v/v, 3.0 mL min^−1^) to afford compounds 5 (19.6 mg; *t*_R_ 31.2 min) and 7 (23.2 mg; *t*_R_ 34.5 min). Repeated purification of fraction E5 using Sephadex LH-20 with CH_3_OH as eluent, RP-C_18_ column (MeOH–H_2_O, from 30 : 70 to 100 : 0, v/v), and semi-preparative HPLC (MeOH–H_2_O, 62 : 38, v/v, 3.0 mL min^−1^) afforded compounds 1 (24.0 mg; *t*_R_ 25.8 min) and 2 (5.8 mg; *t*_R_ 31.5 min).

#### 8′′*R*,9′′-Diol versicolactone B (1)

White, amorphous powder; [*α*]^25^_D_ + 57.0 (*c* 1.00, MeOH); UV (MeOH) *λ*_max_ (log *ε*) = 203 (4.62), 286 (4.13), 330 (3.74) nm; ECD (*c* 0.10, MeOH) = Δ*ε*_202_ + 40.44, Δ*ε*_230_ − 9.96, Δ*ε*_307_ + 2.35; IR *ν*_max_ = 3433, 2976, 1743, 1617, 1501, 1438, 1389, 1259, 1176, 1066, 1041, 798, 763, 695 cm^−1^; HRESIMS *m*/*z* 481.1255 [M + K]^+^ (calcd for C_24_H_26_O_8_K, 481.1265); For ^1^H NMR and ^13^C NMR data, see Table 1.

#### 8′′*S*,9′′-Diol versicolactone B (2)

White, amorphous powder; [*α*]^25^_D_ + 92.0 (*c* 1.00, MeOH); UV (MeOH) *λ*_max_ (log *ε*) = 203 (4.71), 286 (4.24), 330 (3.82) nm; ECD (*c* 0.10, MeOH) = Δ*ε*_201_ + 44.79, Δ*ε*_229_ − 9.30, Δ*ε*_306_ + 1.95; IR *ν*_max_ = 3431, 2924, 2851, 1743, 1640, 1546, 1511, 1502, 1440, 1390, 1260, 1180, 1117, 1066, 1041, 764, 694, 563 cm^−1^; HRESIMS *m*/*z* 465.1529 [M + Na]^+^ (calcd for C_24_H_26_O_8_Na, 465.1525); For ^1^H NMR and ^13^C NMR data, see Table 1.

#### 3′-Isoamylene butyrolactone IV (3)

White, amorphous powder; [*α*]^25^_D_ + 30 (*c* 1.00, MeOH); UV (MeOH) *λ*_max_ (log *ε*) = 202 (4.82), 317 (4.28) nm; ECD (*c* 0.17, MeOH) = Δ*ε*_206_ + 18.93, Δ*ε*_228_ − 3.70, Δ*ε*_283_ –6.97; IR *ν*_max_ = 3437, 2970, 2925, 1746, 1624, 1499, 1443, 1383, 1253, 1175, 1114, 1052 cm^−1^; HRESIMS *m*/*z* 531.1986 [M + Na]^+^ (calcd for C_29_H_32_O_8_Na, 531.1995) and *m*/*z* 547.1751 [M + K]^+^ (calcd for C_29_H_32_O_8_K, 547.1734); For ^1^H NMR and ^13^C NMR data, see Table 1.

#### 4′-Dehydroxy aspernolide A (4)

White, amorphous powder; [*α*]^25^_D_ + 67 (*c* 1.00, MeOH); UV (MeOH) *λ*_max_ (log *ε*) = 203 (4.64), 221 (4.17), 288 (4.16) nm; ECD (*c* 0.17, MeOH) = Δ*ε*_203_ + 35.21, Δ*ε*_227_ − 12.51, Δ*ε*_304_ + 2.59; IR *ν*_max_ = 3434, 2973, 2928, 2856, 1743, 1629, 1498, 1260, 1164, 1120, 1039, 764, 608 cm^−1^; HRESIMS *m*/*z* 431.1464 [M + Na]^+^ (calcd for C_24_H_24_O_6_Na, 431.1471); For ^1^H NMR and ^13^C NMR data, see Table 1.

### [Mo_2_(AcO)_4_] induced circular dichroism

[Mo_2_(AcO)_4_] (1 mg) dissolved in DMSO (1 mL) was prepared as the stock solution, to which compounds 1 and 2 (each 0.5 mg) were added, respectively. The circular dichroism (CD) spectra were recorded immediately after mixing and scanned every 10 min for 30 min, to afford the stationary [Mo_2_(AcO)_4_] induced circular dichroism spectra for each compound.

### ^13^C NMR calculations

The conformations generated by BALLOON were subjected to semiempirical PM3 quantum mechanical geometry optimizations using the Gaussian 09 program.^22^ Duplicate conformations were identified and removed when the root-mean-square (RMS) distance was less than 0.5 Å for any two geometry-optimized conformations. The remaining conformations were further optimized at the B3LYP/6-31G(d) level in chloroform with the IEFPCM solvation model using Gaussian 09, and the duplicate conformations emerging after these calculations were removed according to the same RMS criteria above. The number of conformers from the conformational search and final optimization for compounds 1, 5, and 7 were 400 to 9, 259 to 10, and 160 to 11, respectively. The harmonic vibrational frequencies were calculated to confirm the stability of the final conformers. The NMR chemical shifts were calculated for each conformer at the B3LYP/6-311++G(d,p)//B3LYP/6-31G(d) level with chloroform as solvent by the IEFPCM solvation model implemented in Gaussian 09 program, which were then combined using Boltzmann weighting according to their population contributions.

### Anti-inflammatory assay

The anti-inflammatory activity of compounds 1–10 was assessed by measuring the amount of NO production in LPS-induced RAW 264.7 mouse macrophages (positive control, indomethacin), according to the previously described method.^23^

## Conflicts of interest

There are no conflicts to declare.

## Supplementary Material

RA-008-C8RA01840E-s001
