# Supplementary material for: Anti-inflammatory butenolide derivatives from the coral-derived fungus Aspergillus terreus and structure revisions of aspernolides D and G, butyrolactone VI and 4′,8′′-diacetoxy butyrolactone VI
Source: RSC Adv. 2018 Apr 9;8(23):13040–7. doi: 10.1039/c8ra01840e (PMC9079733; doi:10.1039/c8ra01840e)
Supplement: RA-008-C8RA01840E-s001 [file RA-008-C8RA01840E-s001.pdf]

Electronic Supplementary Material (ESI) for RSC Advances.
This journal is © The Royal Society of Chemistry 2018

Anti-inflammatory butenolide derivatives from the coral-derived fungus *Aspergillus terreus* and structure revisions of aspernolides D and G, butyrolactone VI and 4′,8″-diacetoxy butyrolactone VI

Mengting Liu,[a,†] Qun Zhou,[a,†] Jianping Wang,[a,†] Junjun Liu,[a] Changxing Qi,[a] Yongji Lai,[b] Hucheng Zhu,[a] Yongbo Xue,[a] Zhengxi Hu,[a,*] and Yonghui Zhang[a,*]

[a]*Hubei Key Laboratory of Natural Medicinal Chemistry and Resource Evaluation, School of Pharmacy, Tongji Medical College, Huazhong University of Science and Technology, Wuhan 430030, Hubei Province, People's Republic of China*

[b]*Department of Pharmacy, the Central Hospital of Wuhan, Wuhan 430014, Hubei Province, People's Republic of China*

---

* Corresponding author Tel.: +86 027 83692892.
E-mail addresses: zhangyh@mails.tjmu.edu.cn (Y. Zhang); hzx616@126.com (Z. Hu).

CONTENTS

Figure S1. ^{1}H NMR spectrum of compound **1** (Recorded in $CDCl_3$) 1
Figure S2. ^{13}C NMR spectrum of compound **1** (Recorded in $CDCl_3$) 2
Figure S3. DEPT spectrum of compound **1** (Recorded in $CDCl_3$) 3
Figure S4. HSQC spectrum of compound **1** (Recorded in $CDCl_3$) 4
Figure S5. HMBC spectrum of compound **1** (Recorded in $CDCl_3$) 5
Figure S6. ^{1}H–^{1}H COSY spectrum of compound **1** (Recorded in $CDCl_3$) 6
Figure S7. NOESY spectrum of compound **1** (Recorded in $CDCl_3$) 7
Figure S8. HRESIMS spectrum of compound **1** 8
Figure S9. UV spectrum of compound **1** 9
Figure S10. IR spectrum of compound **1** 10
Figure S11. ^{1}H NMR spectrum of compound **2** (Recorded in $CDCl_3$) 11
Figure S12. ^{13}C NMR spectrum of compound **2** (Recorded in $CDCl_3$) 12
Figure S13. DEPT spectrum of compound **2** (Recorded in $CDCl_3$) 13
Figure S14. HSQC spectrum of compound **2** (Recorded in $CDCl_3$) 14
Figure S15. HMBC spectrum of compound **2** (Recorded in $CDCl_3$) 15
Figure S16. ^{1}H–^{1}H COSY spectrum of compound **2** (Recorded in $CDCl_3$) 16
Figure S17. NOESY spectrum of compound **2** (Recorded in $CDCl_3$) 17
Figure S18. HRESIMS spectrum of compound **2** 18
Figure S19. UV spectrum of compound **2** 19
Figure S20. IR spectrum of compound **2** 20
Figure S21. ^{1}H NMR spectrum of compound **3** (Recorded in methanol-d_4) 21
Figure S22. ^{13}C NMR spectrum of compound **3** (Recorded in methanol-d_4) 22
Figure S23. DEPT spectrum of compound **3** (Recorded in methanol-d_4) 23
Figure S24. HSQC spectrum of compound **3** (Recorded in methanol-d_4) 24

Figure S25. HMBC spectrum of compound **3** (Recorded in methanol-d_4)........25
Figure S26. ^{1}H–^{1}H COSY spectrum of compound **3** (Recorded in methanol-d_4)........26
Figure S27. NOESY spectrum of compound **3** (Recorded in methanol-d_4)........27
Figure S28. HRESIMS spectrum of compound **3**........28
Figure S29. UV spectrum of compound **3**........29
Figure S30. IR spectrum of compound **3**........30
Figure S31. ^{1}H NMR spectrum of compound **4** (Recorded in methanol-d_4)........31
Figure S32. ^{13}C NMR spectrum of compound **4** (Recorded in methanol-d_4)........32
Figure S33. DEPT spectrum of compound **4** (Recorded in methanol-d_4)........33
Figure S34. HSQC spectrum of compound **4** (Recorded in methanol-d_4)........34
Figure S35. HMBC spectrum of compound **4** (Recorded in methanol-d_4)........35
Figure S36. ^{1}H–^{1}H COSY spectrum of compound **4** (Recorded in methanol-d_4)........36
Figure S37. NOESY spectrum of compound **4** (Recorded in methanol-d_4)........37
Figure S38. HRESIMS spectrum of compound **4**........38
Figure S39. UV spectrum of compound **4**........39
Figure S40. IR spectrum of compound **4**........40
Figure S41. ^{1}H NMR spectrum of compound **5** (Recorded in methanol-d_4)........41
Figure S42. ^{13}C NMR spectrum of compound **5** (Recorded in methanol-d_4)........42
Figure S43. DEPT spectrum of compound **5** (Recorded in methanol-d_4)........43
Figure S44. HRESIMS spectrum of compound **5**........44
Figure S45. ^{1}H NMR spectrum of compound **7** (Recorded in methanol-d_4)........45
Figure S46. ^{13}C NMR spectrum of compound **7** (Recorded in methanol-d_4)........46
Figure S47. DEPT spectrum of compound **7** (Recorded in methanol-d_4)........47
Figure S48. HRESIMS spectrum of compound **7**........48
Figure S49. ^{13}C NMR prediction at C-7″ for compound **1** by ACD/Labs software........49
Figure S50. ^{13}C NMR prediction at C-8″ for compound **1** by ACD/Labs software........50

Figure S51. ^{13}C NMR prediction at C-9″ for compound **1** by ACD/Labs software 51
Figure S52. ^{13}C NMR prediction at C-7″ for compound **5** by ACD/Labs software 52
Figure S53. ^{13}C NMR prediction at C-8″ for compound **5** by ACD/Labs software 53
Figure S54. ^{13}C NMR prediction at C-9″ for compound **5** by ACD/Labs software 54
Figure S55. ^{13}C NMR prediction at C-7″ for compound **7** by ACD/Labs software 55
Figure S56. ^{13}C NMR prediction at C-8″ for compound **7** by ACD/Labs software 56
Figure S57. ^{13}C NMR prediction at C-9″ for compound **7** by ACD/Labs software 57
^{13}C NMR calculation data 58

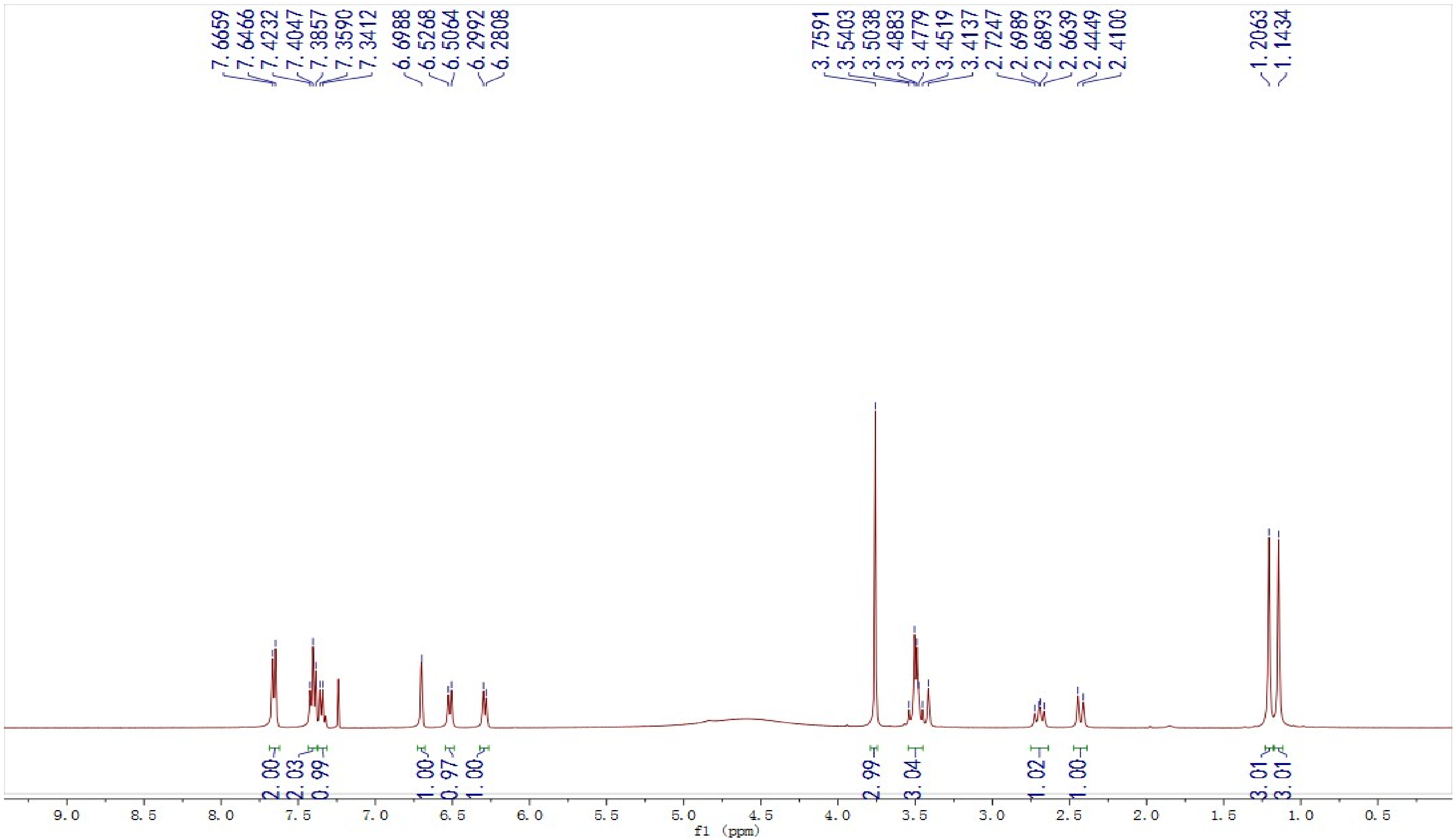

Figure S1. ^{1}H NMR spectrum of compound **1** (Recorded in $CDCl_3$)

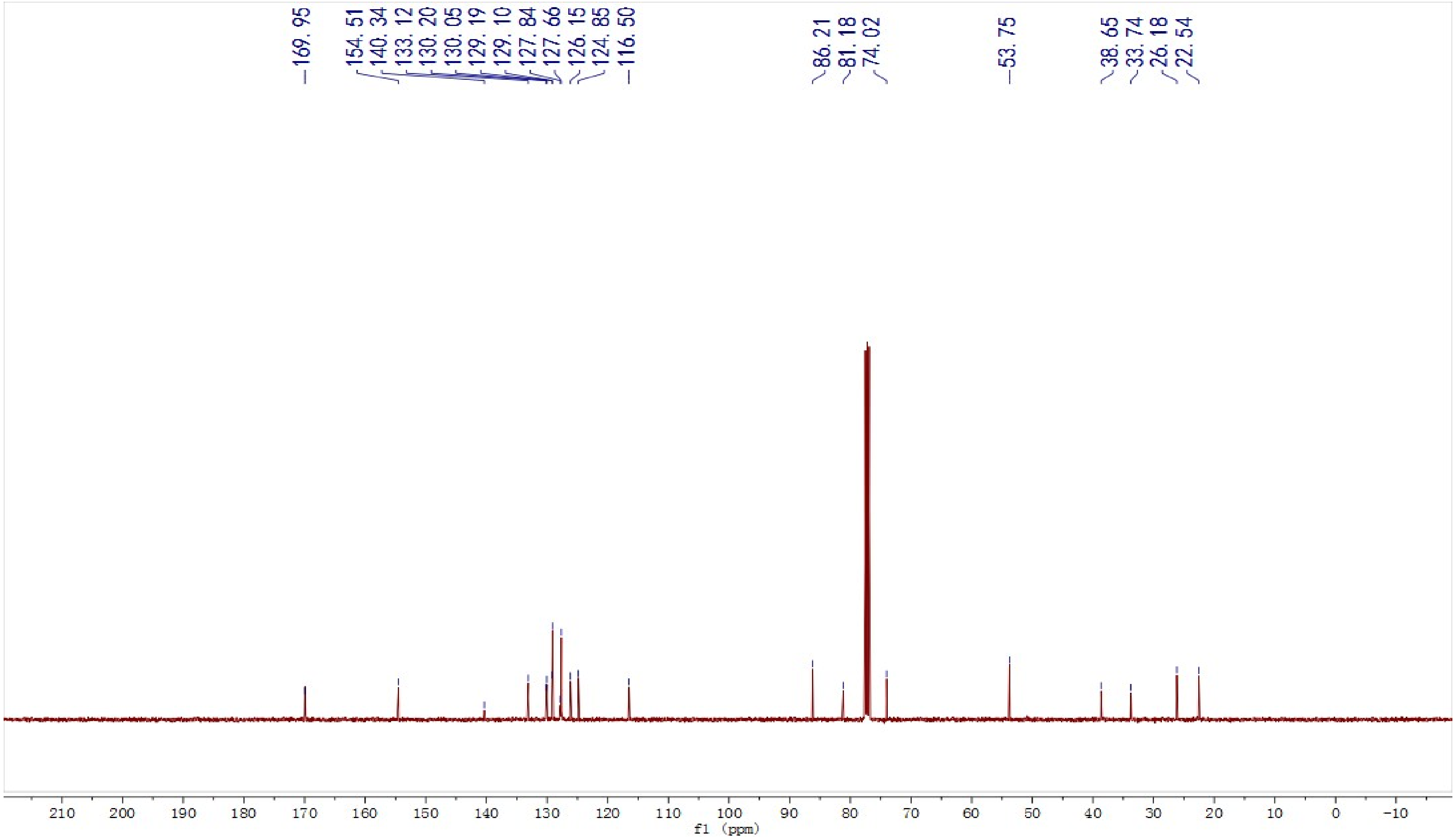

Figure S2. ^{13}C NMR spectrum of compound **1** (Recorded in $CDCl_3$)

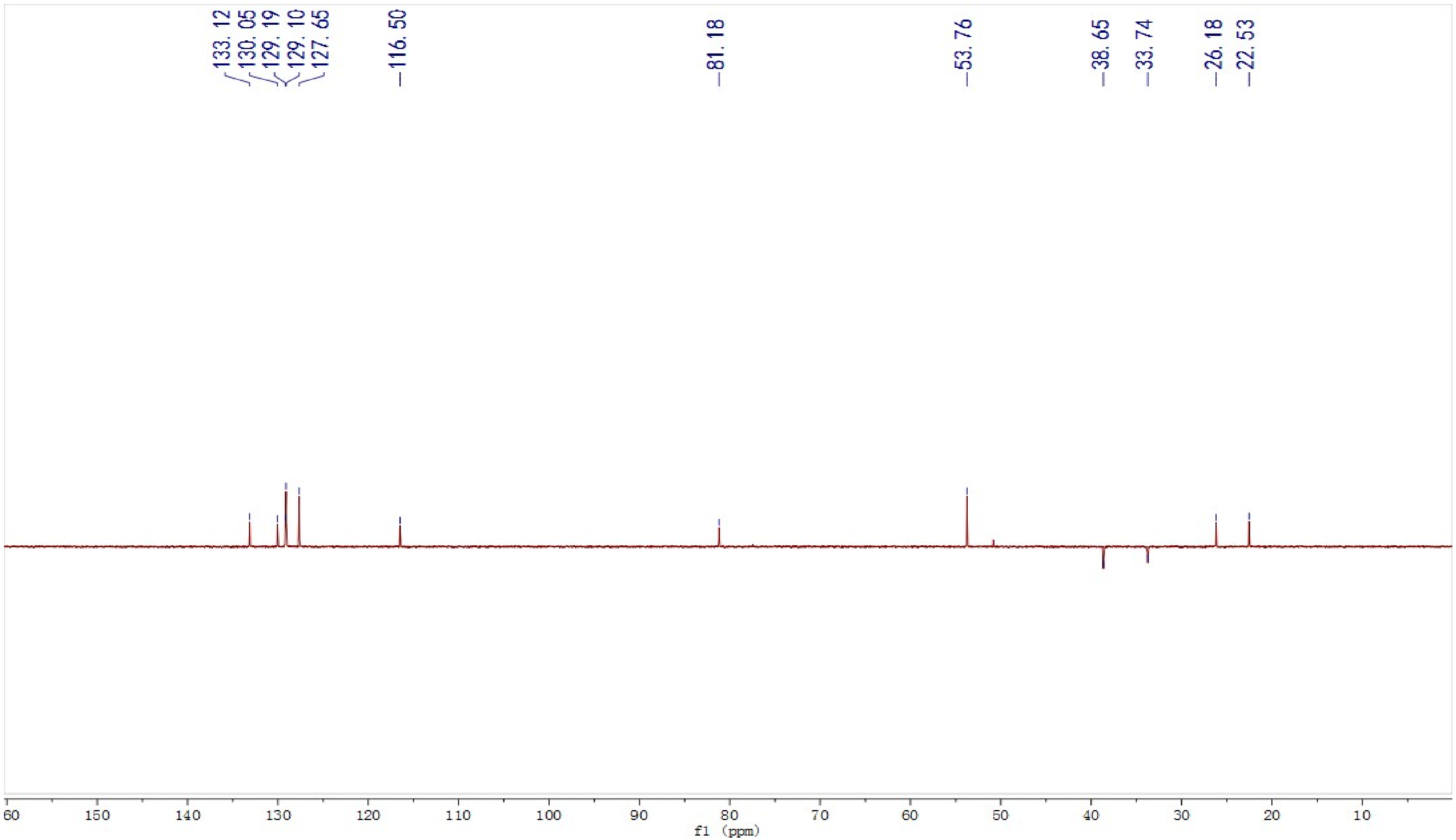

Figure S3. DEPT spectrum of compound **1** (Recorded in $CDCl_3$)

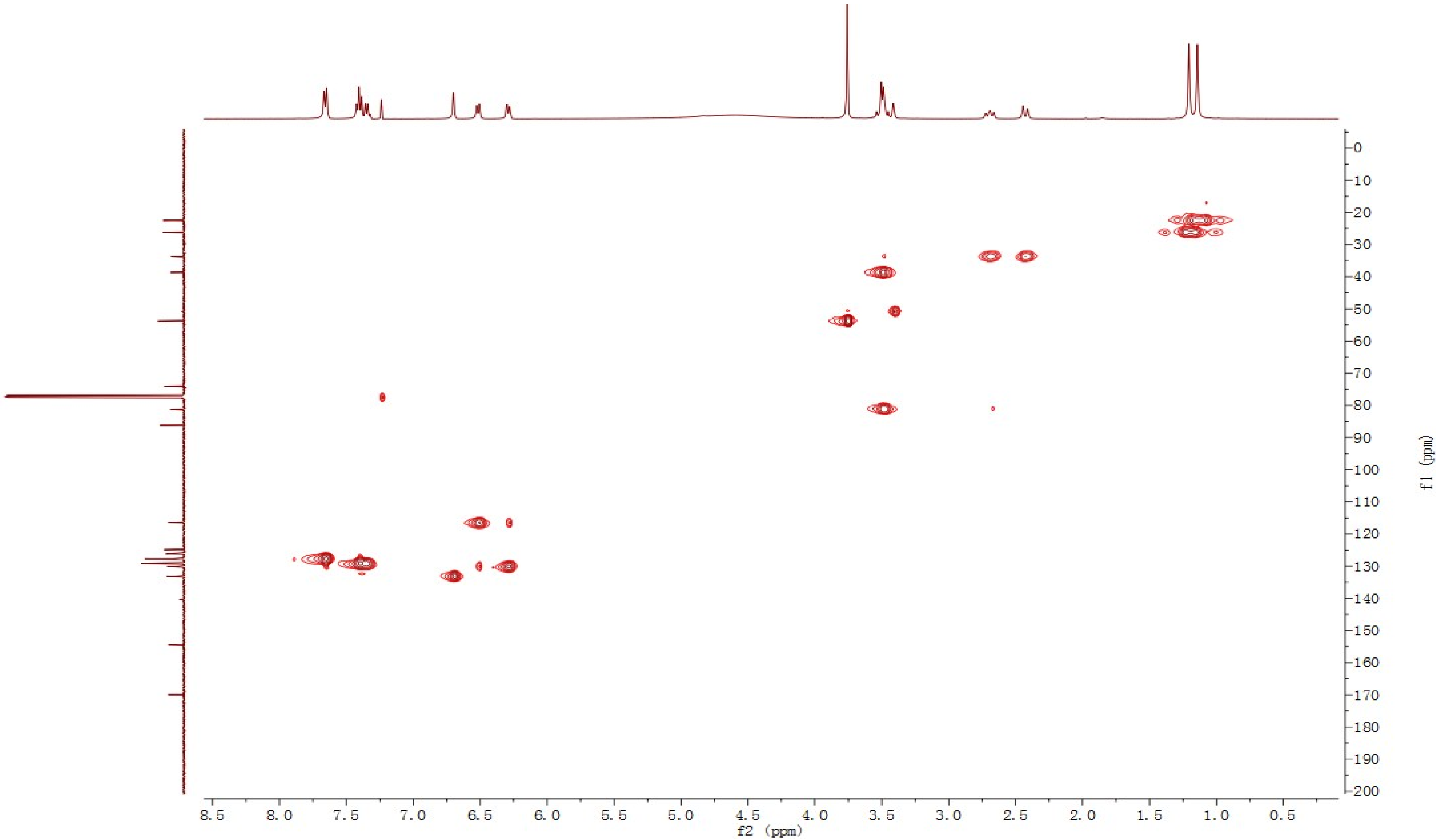

Figure S4. HSQC spectrum of compound **1** (Recorded in $CDCl_3$)

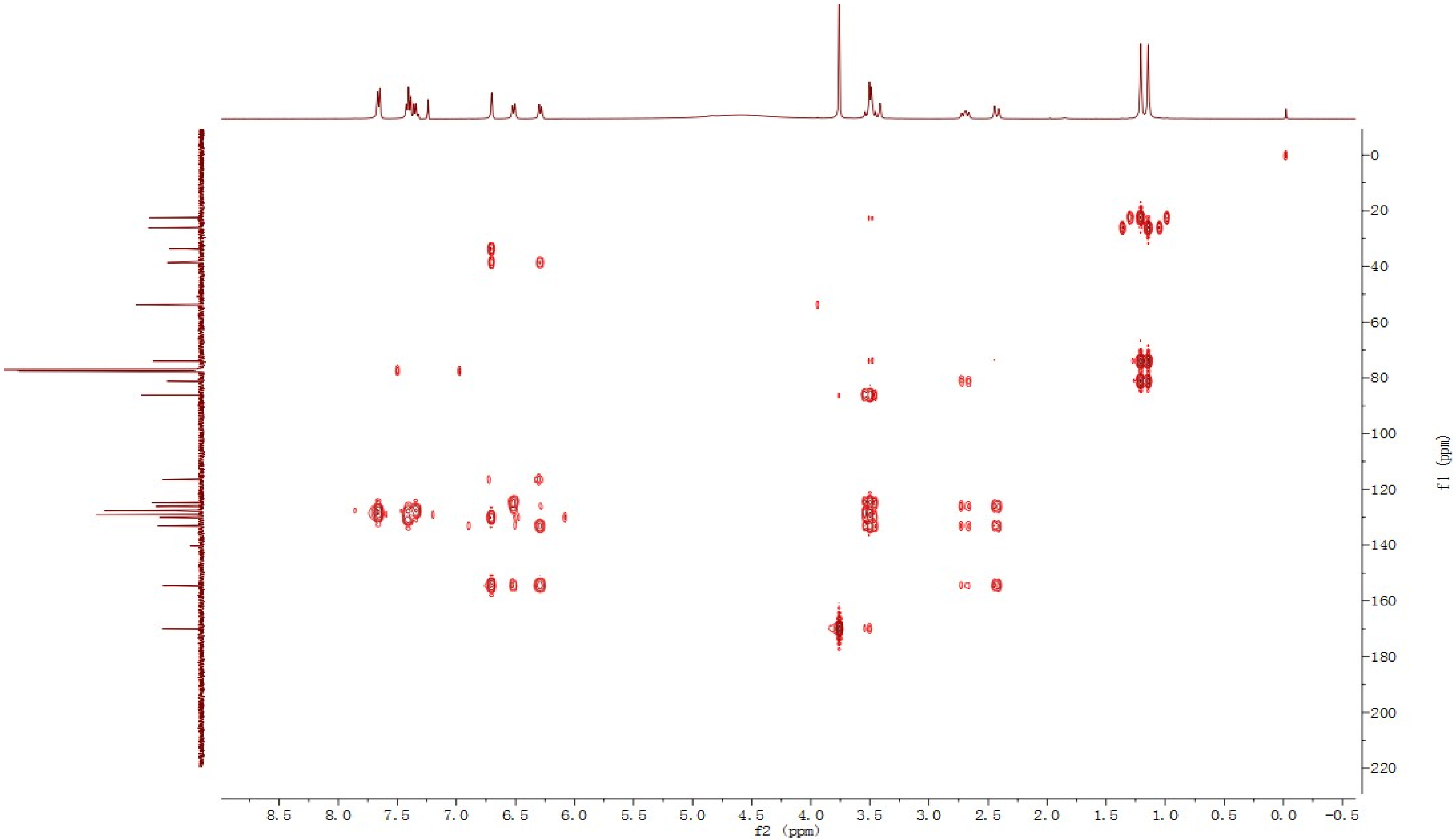

Figure S5. HMBC spectrum of compound **1** (Recorded in $CDCl_3$)

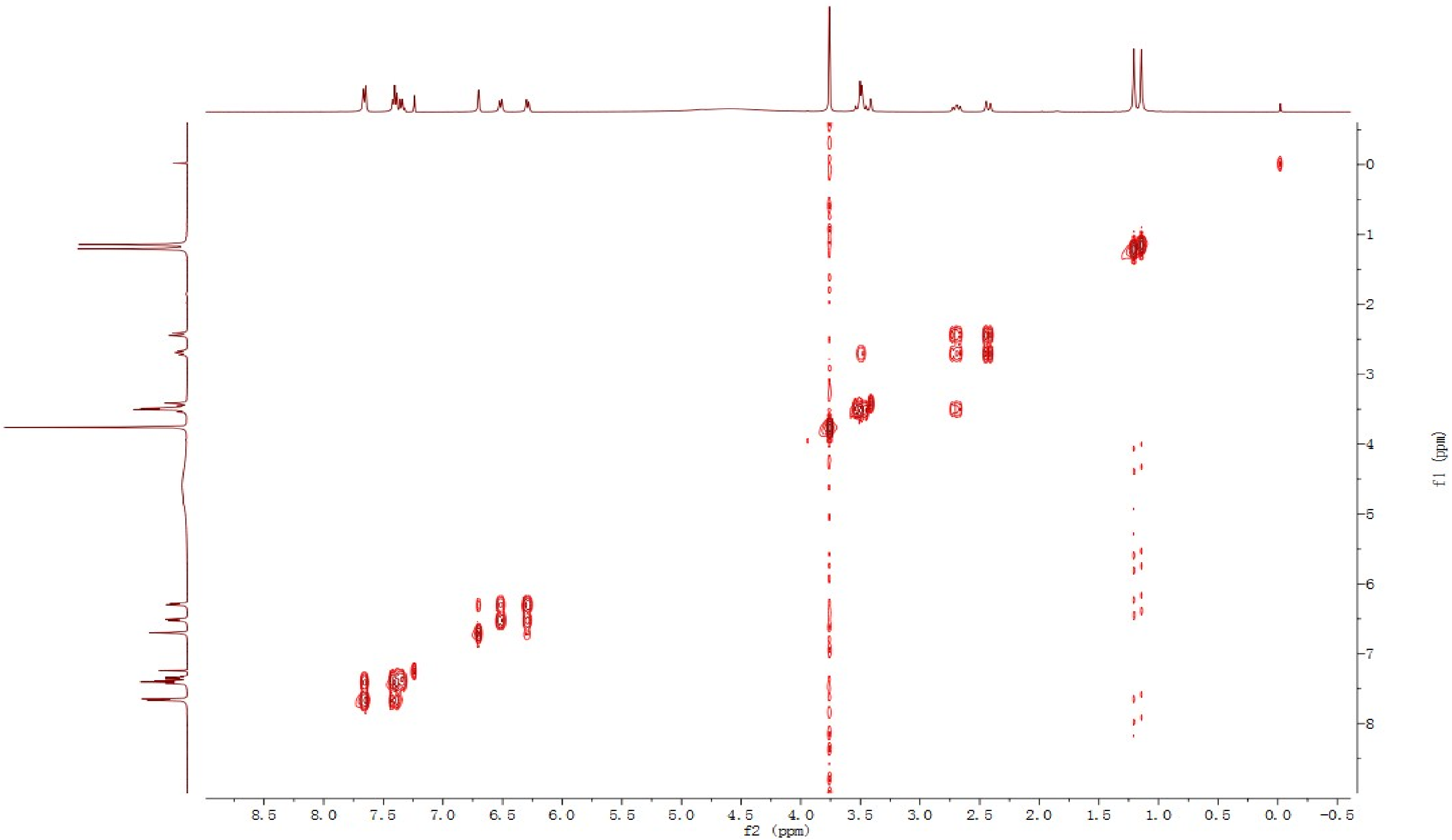

Figure S6. ^{1}H–^{1}H COSY spectrum of compound **1** (Recorded in $CDCl_3$)

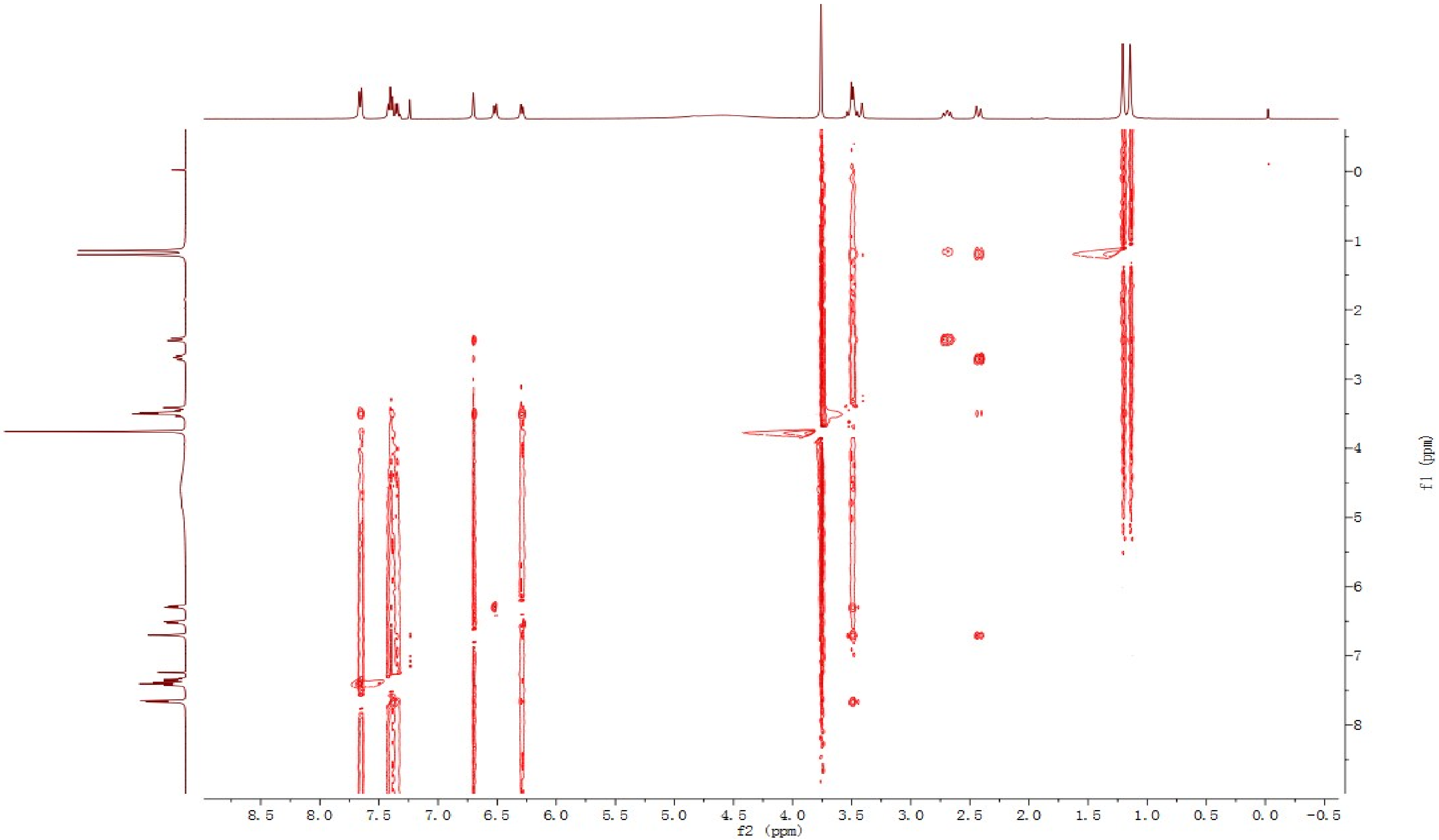

Figure S7. NOESY spectrum of compound **1** (Recorded in $CDCl_3$)

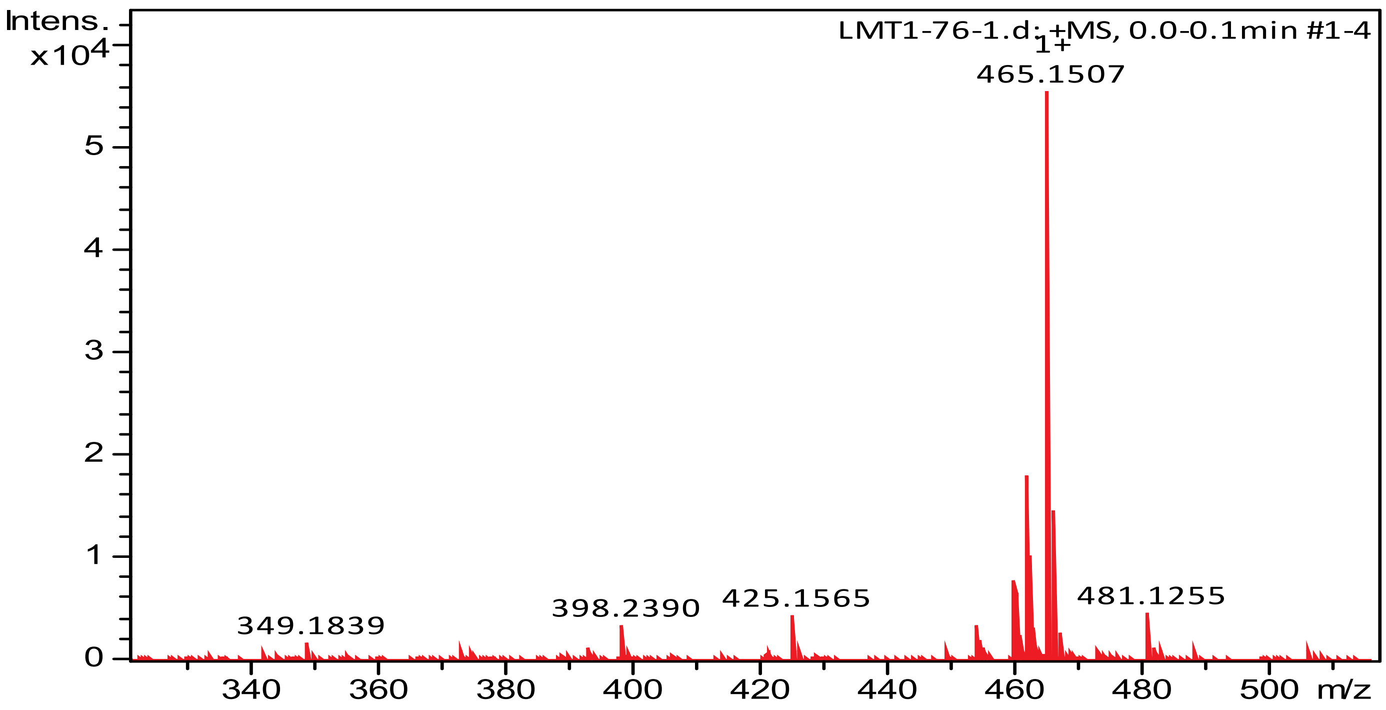

Figure S8. HRESIMS spectrum of compound **1**

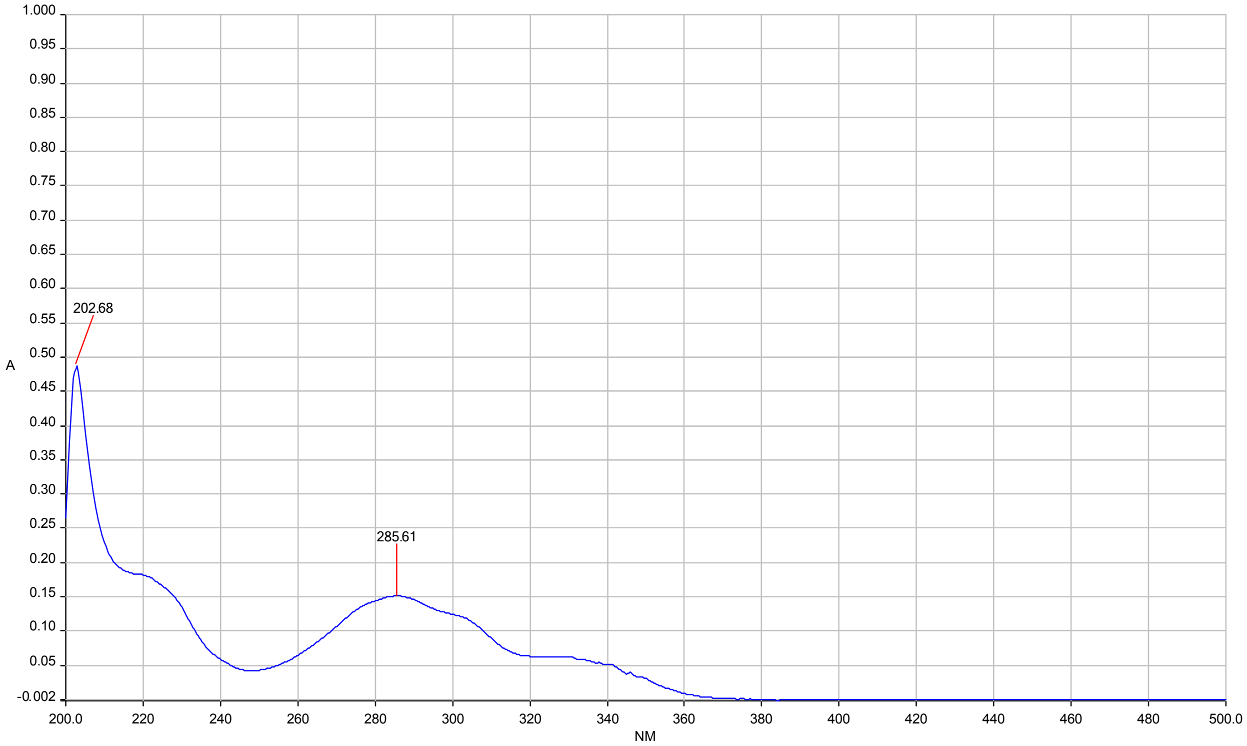

Figure S9. UV spectrum of compound **1**

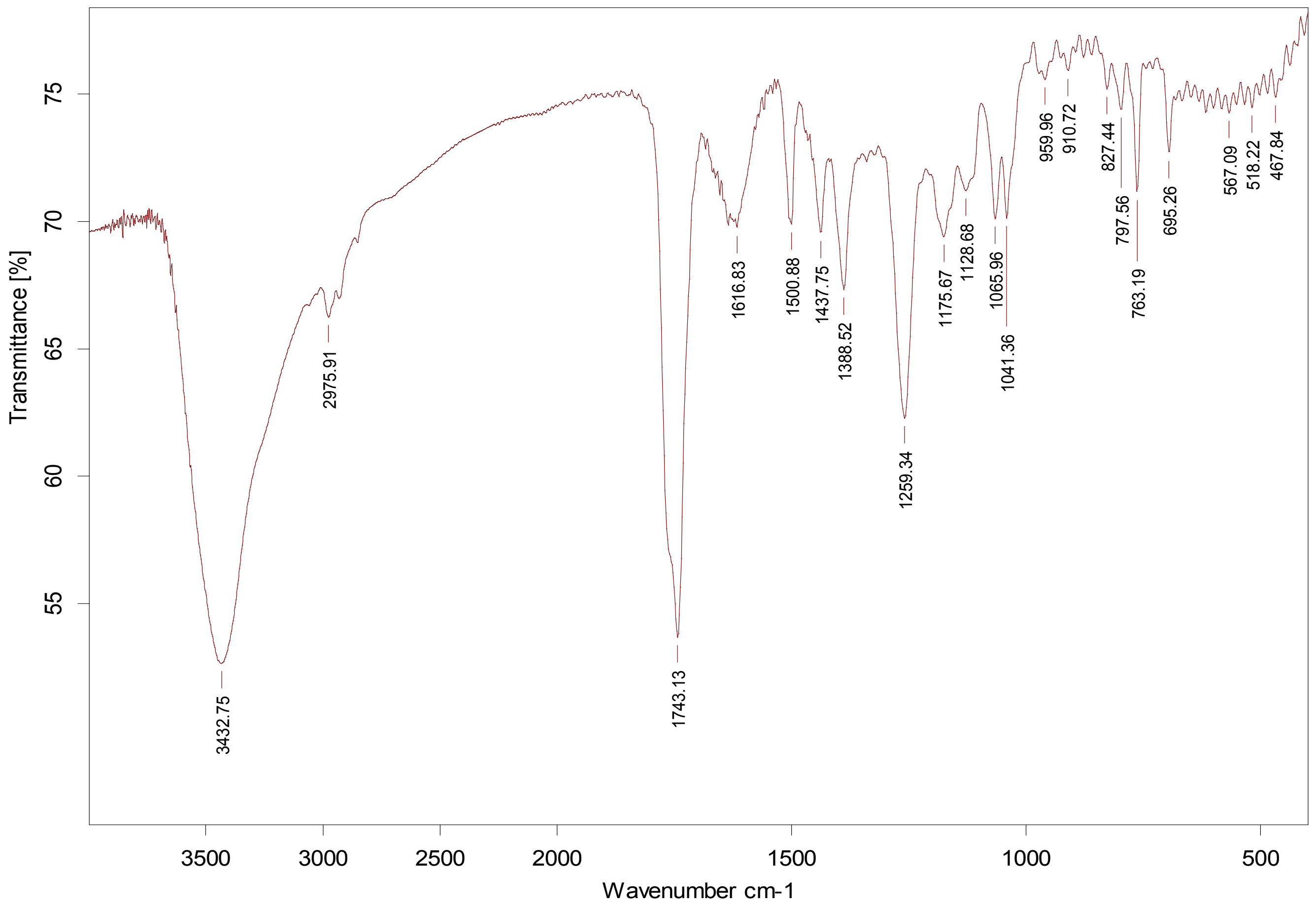

Figure S10. IR spectrum of compound **1**

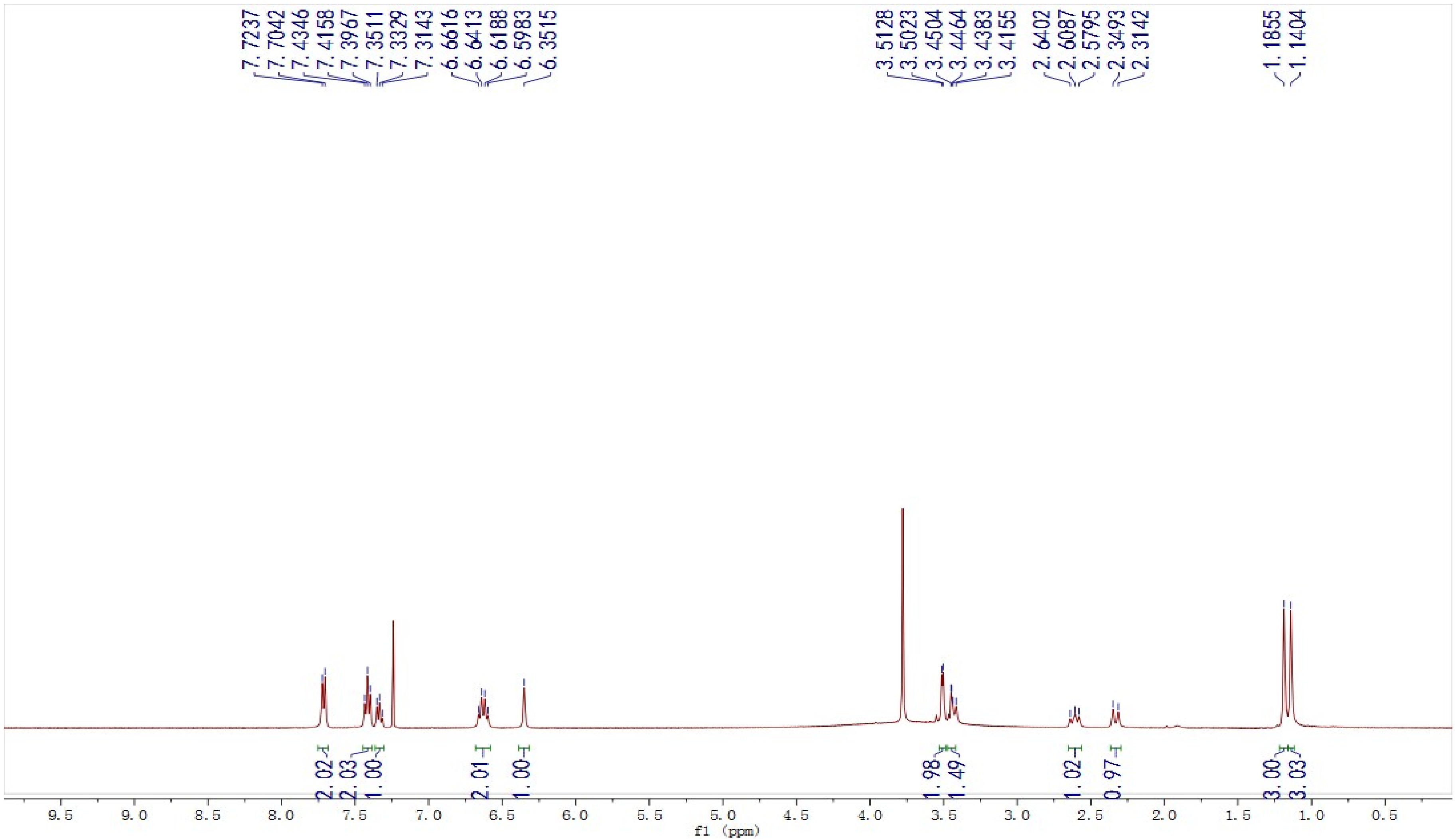

Figure S11. ^{1}H NMR spectrum of compound **2** (Recorded in $CDCl_3$)

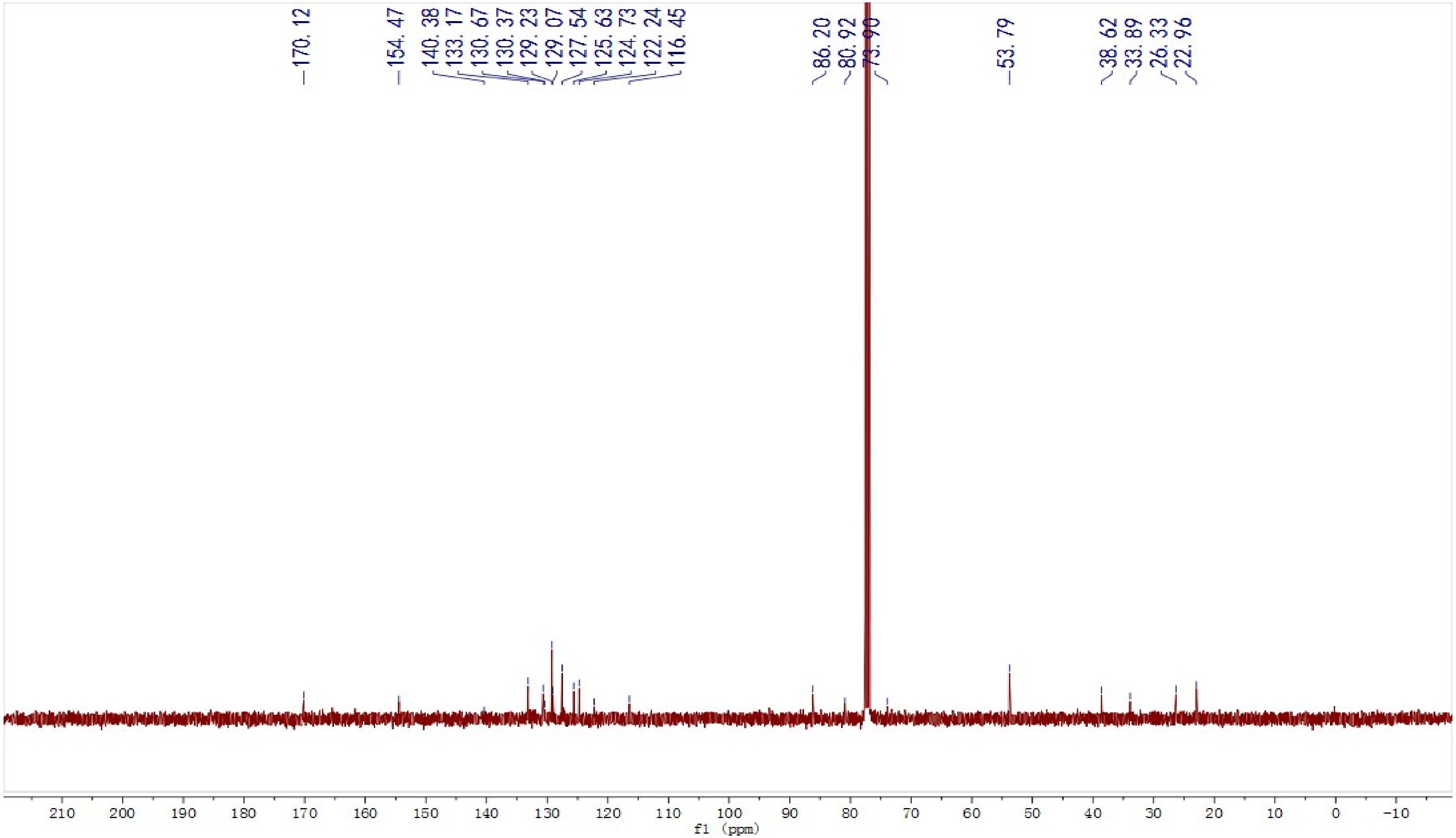

Figure S12. ^{13}C NMR spectrum of compound **2** (Recorded in $CDCl_3$)

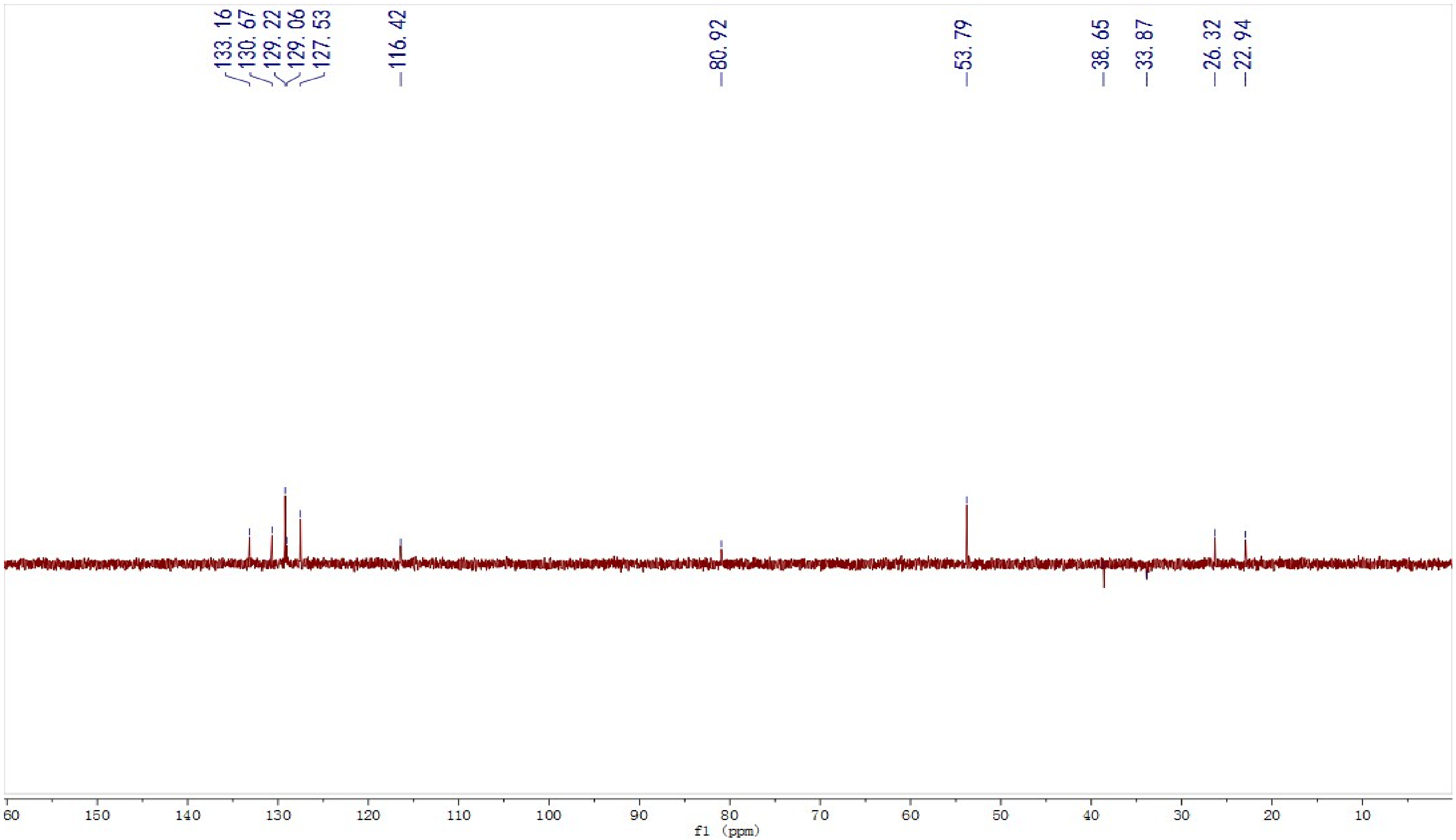

Figure S13. DEPT spectrum of compound **2** (Recorded in $CDCl_3$)

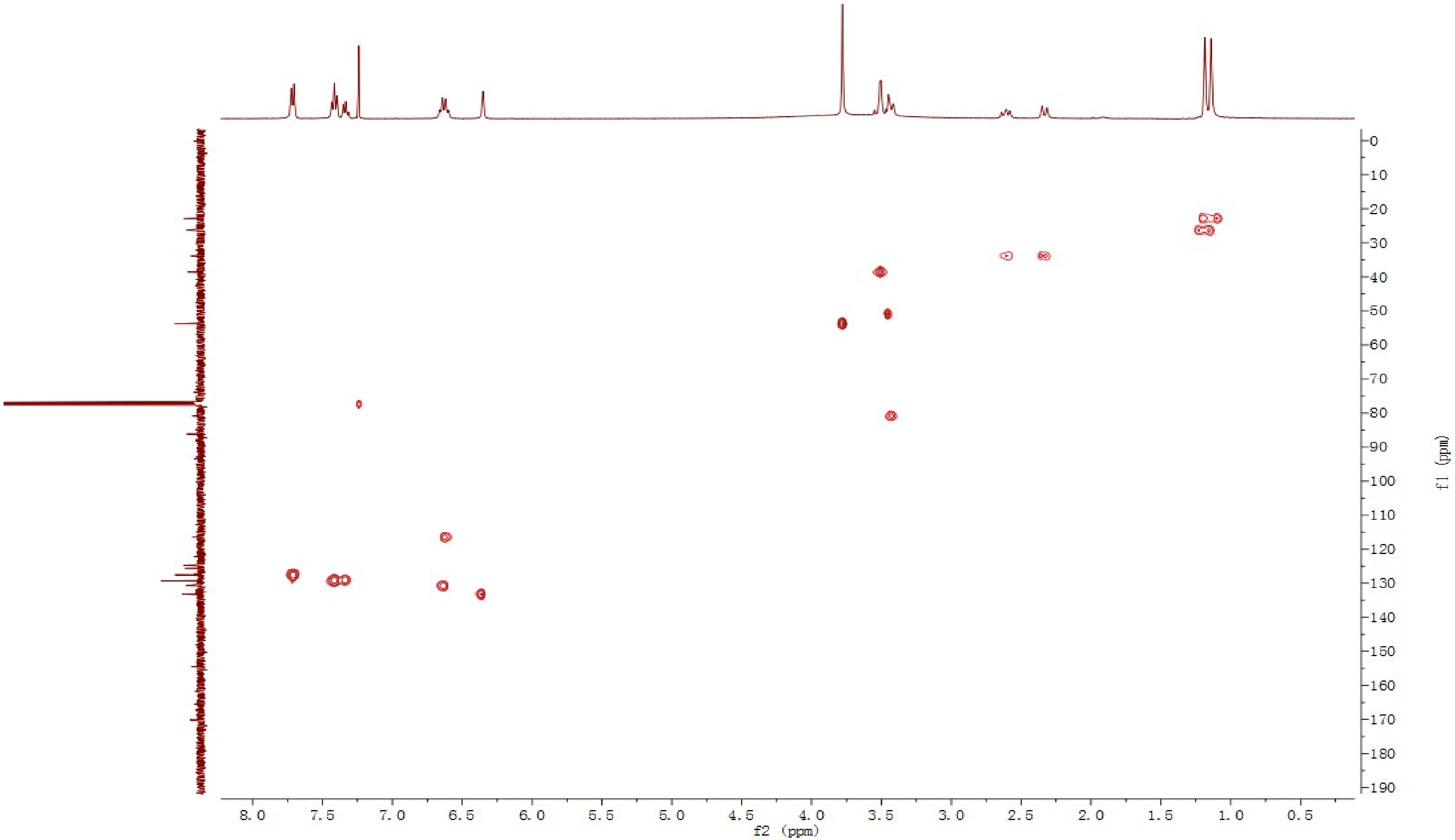

Figure S14. HSQC spectrum of compound **2** (Recorded in $CDCl_3$)

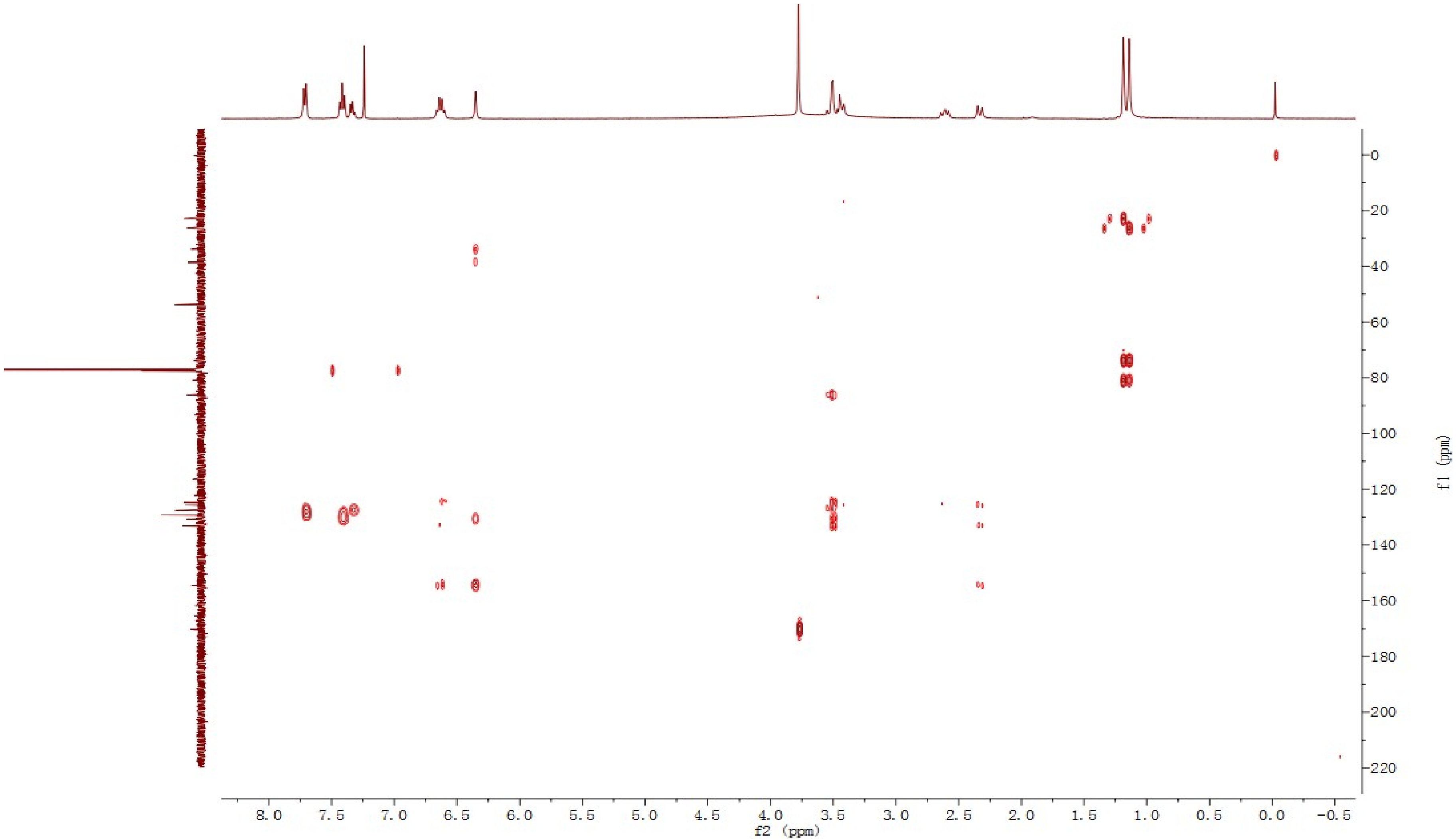

Figure S15. HMBC spectrum of compound **2** (Recorded in $CDCl_3$)

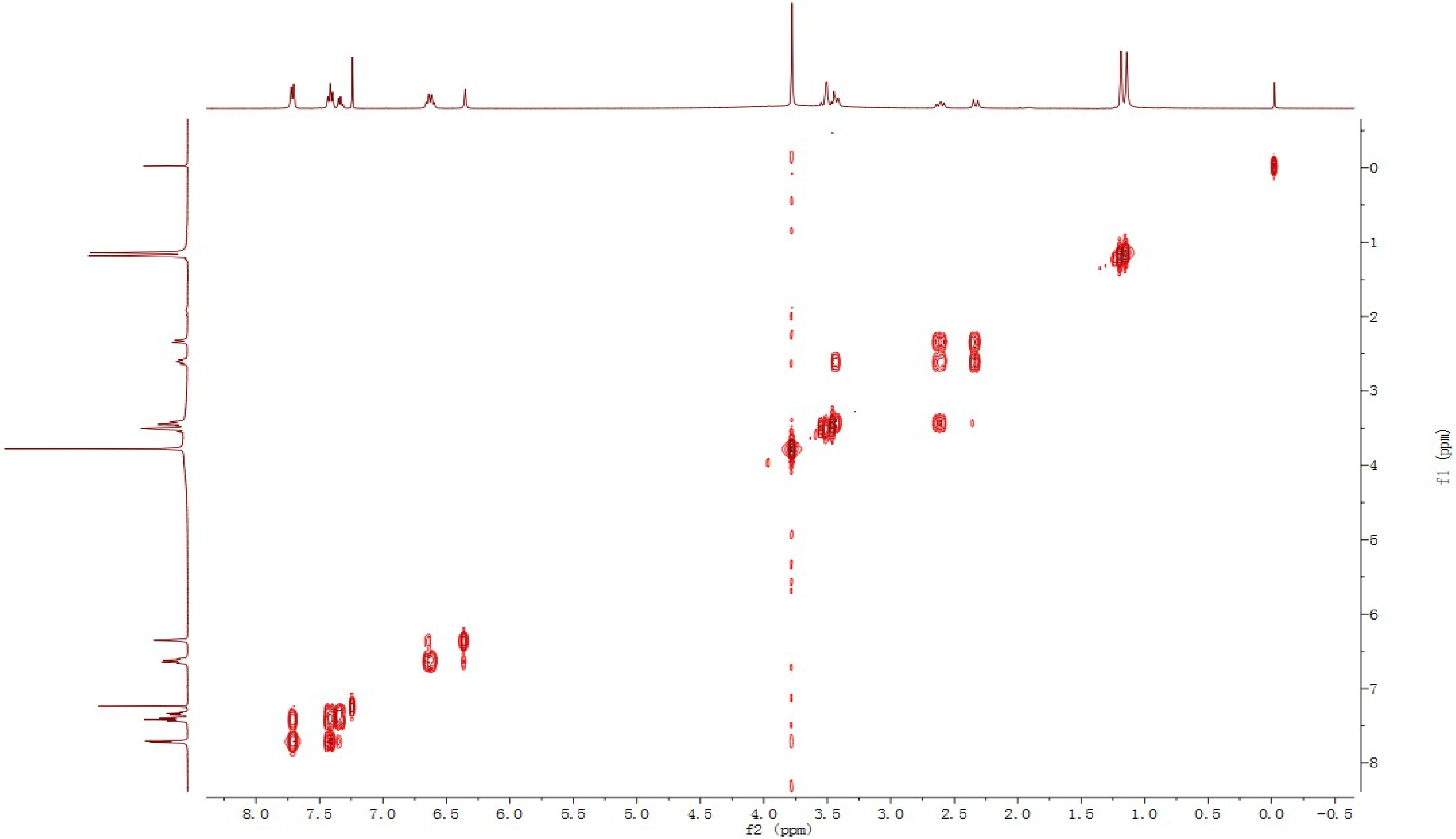

Figure S16. ^{1}H–^{1}H COSY spectrum of compound **2** (Recorded in $CDCl_3$)

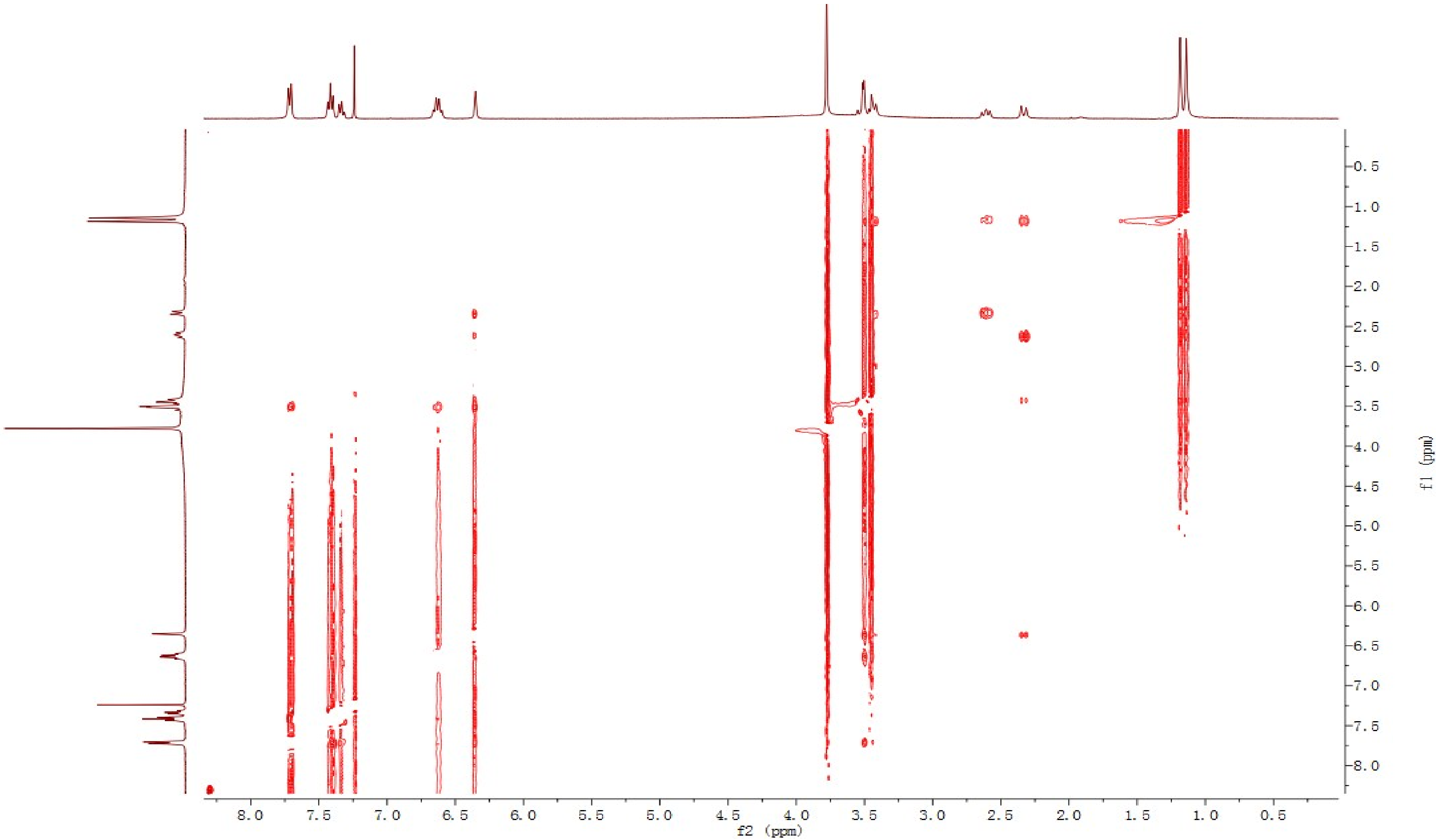

Figure S17. NOESY spectrum of compound **2** (Recorded in $CDCl_3$)

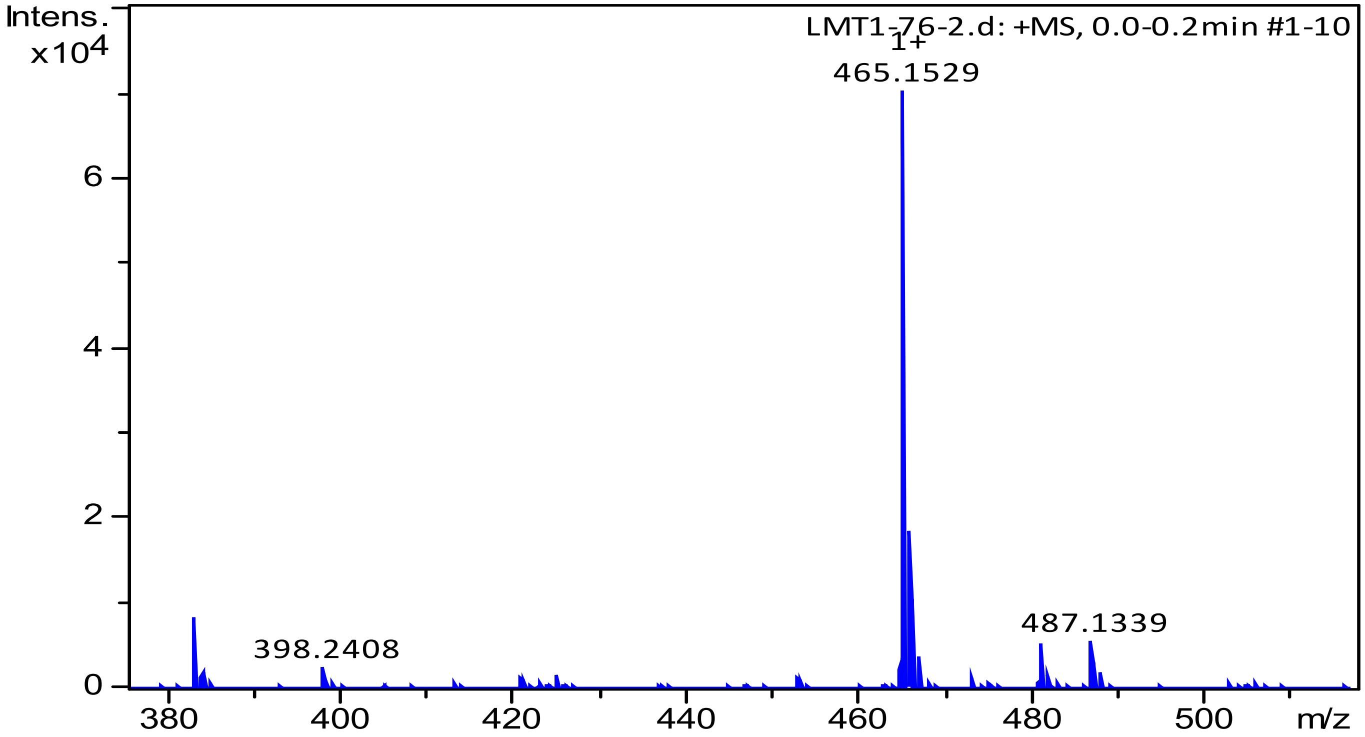

Figure S18. HRESIMS spectrum of compound **2**

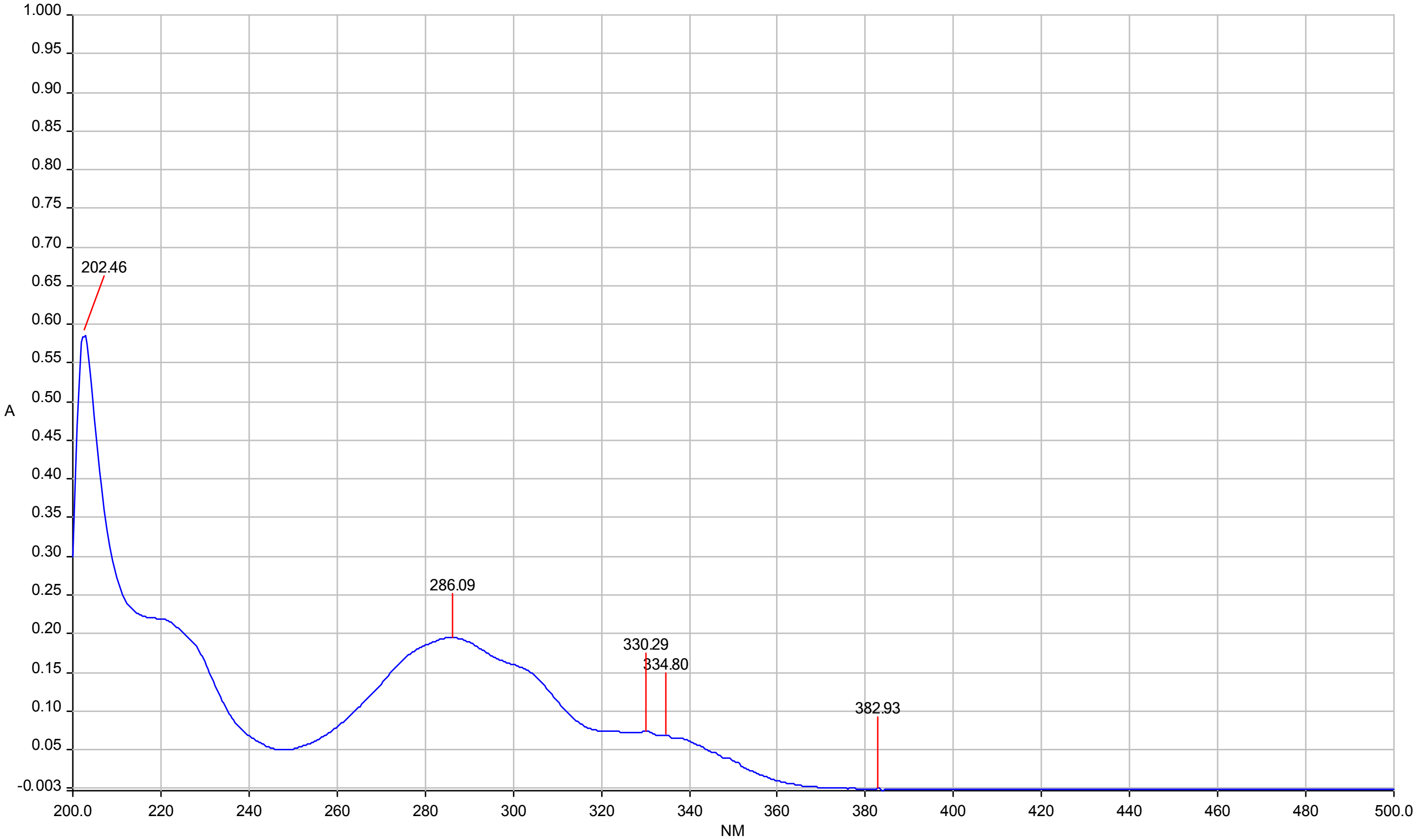

Figure S19. UV spectrum of compound **2**

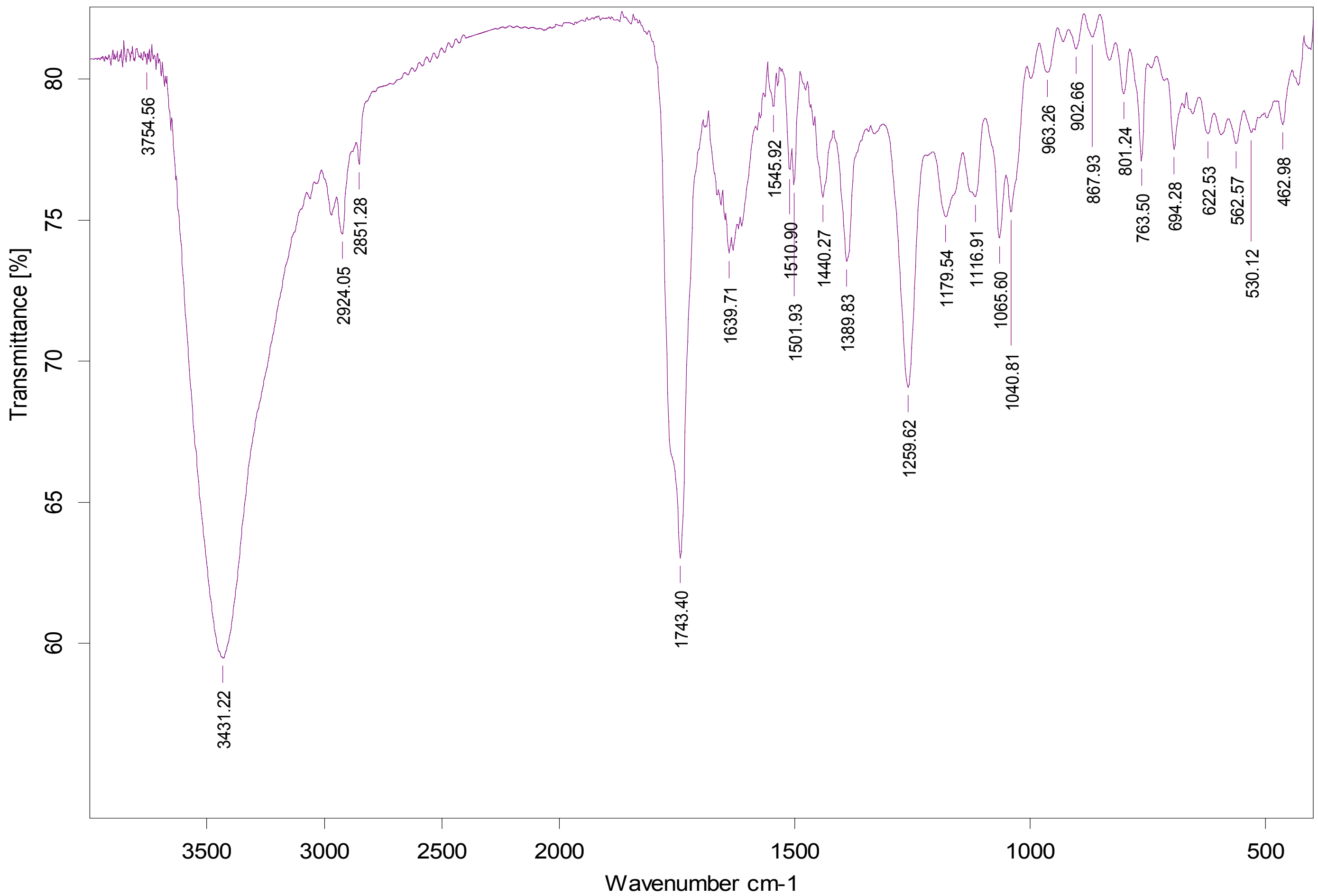

Figure S20. IR spectrum of compound **2**

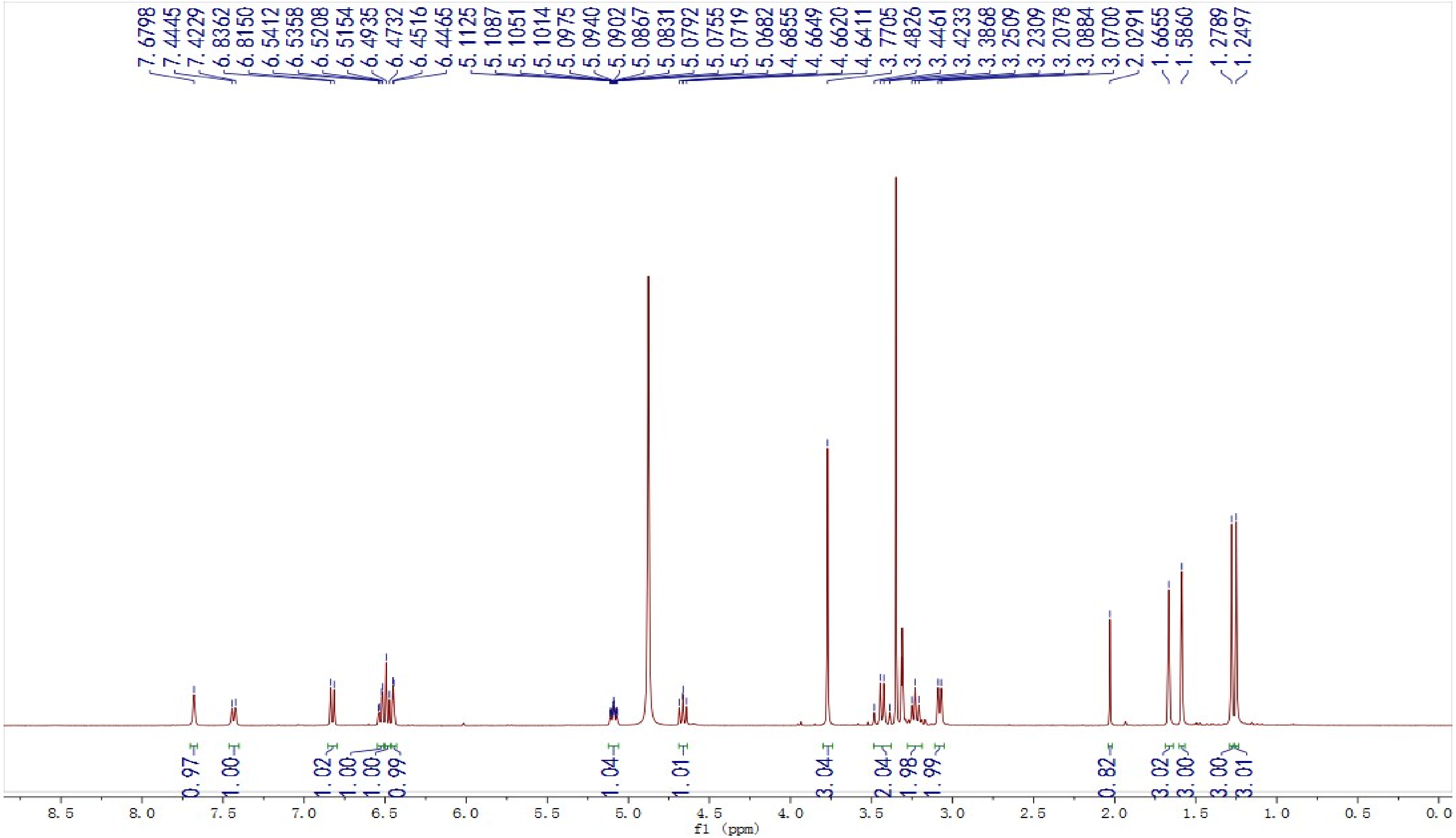

Figure S21. ^{1}H NMR spectrum of compound **3** (Recorded in methanol-d_4)

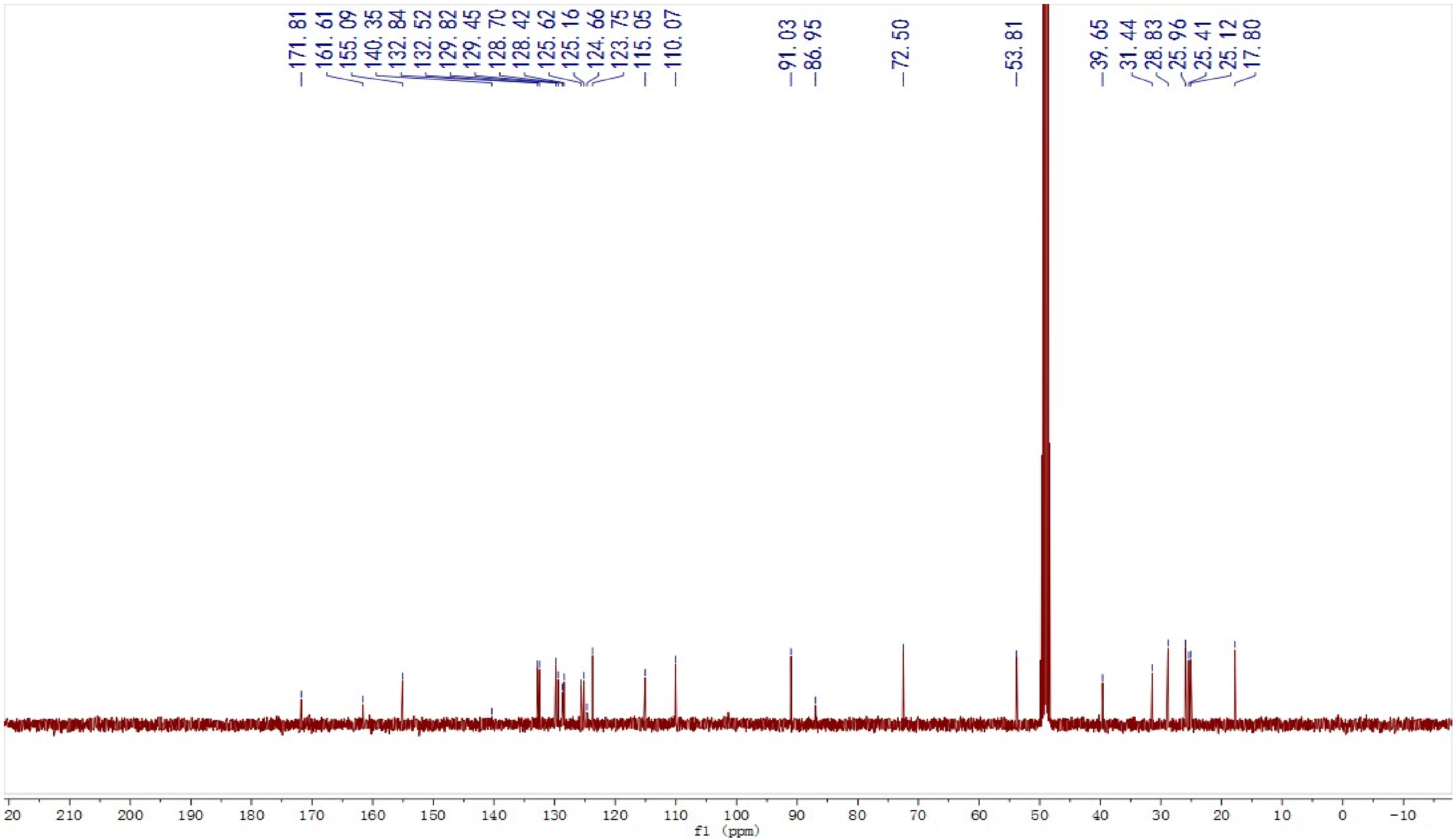

Figure S22. ^{13}C NMR spectrum of compound **3** (Recorded in methanol-d_4)

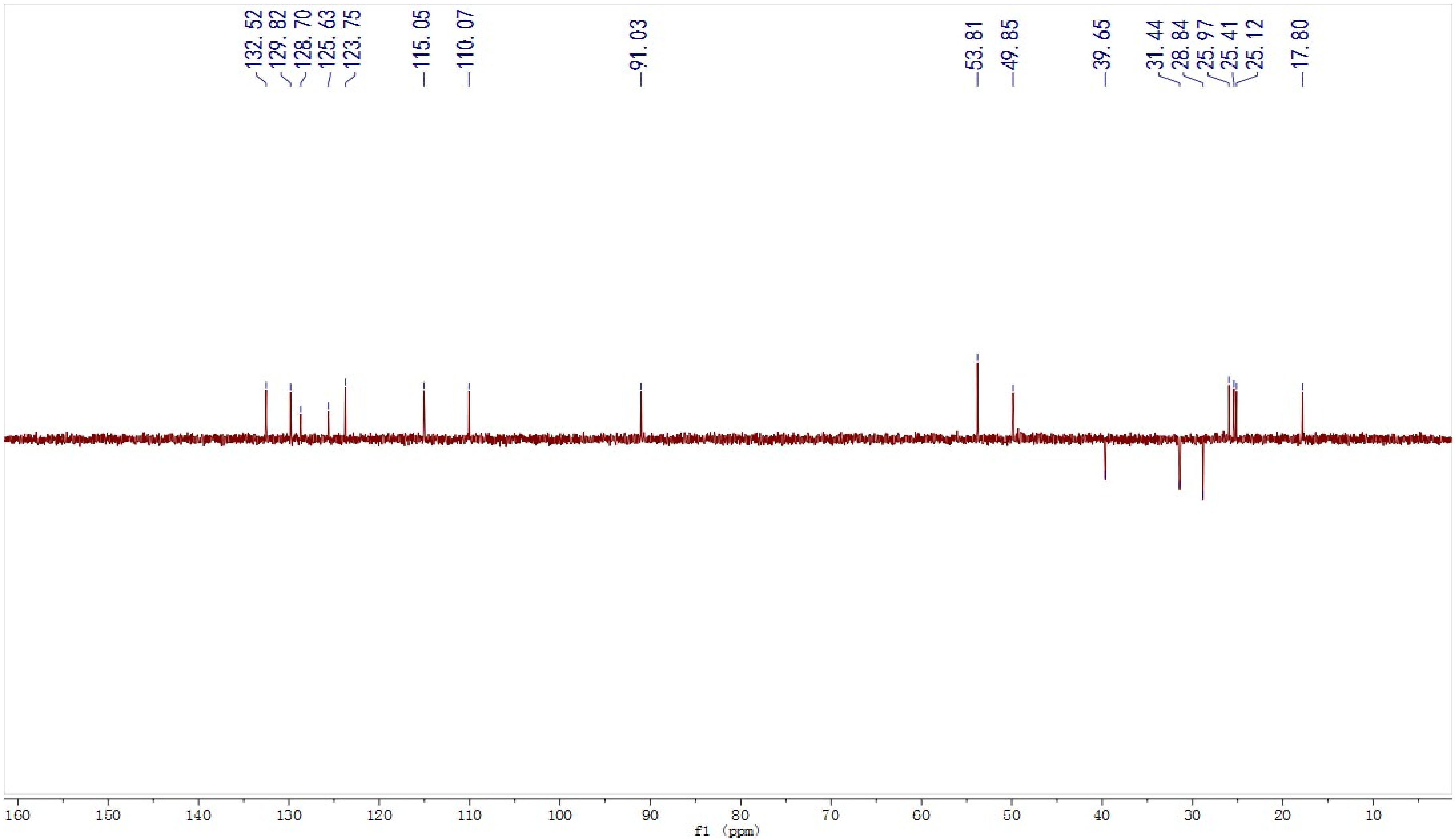

Figure S23. DEPT spectrum of compound **3** (Recorded in methanol-d_4)

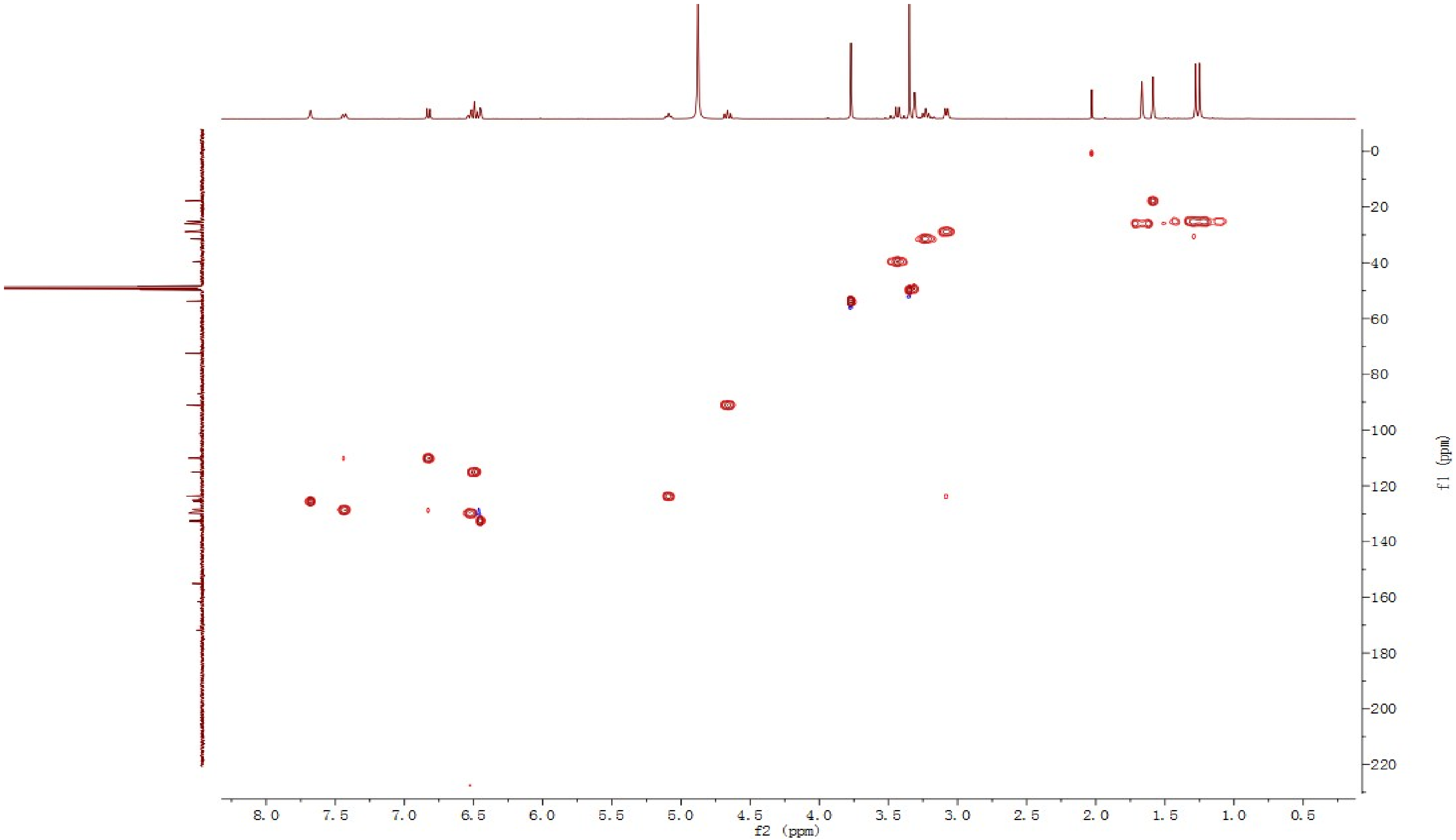

Figure S24. HSQC spectrum of compound **3** (Recorded in methanol-d_4)

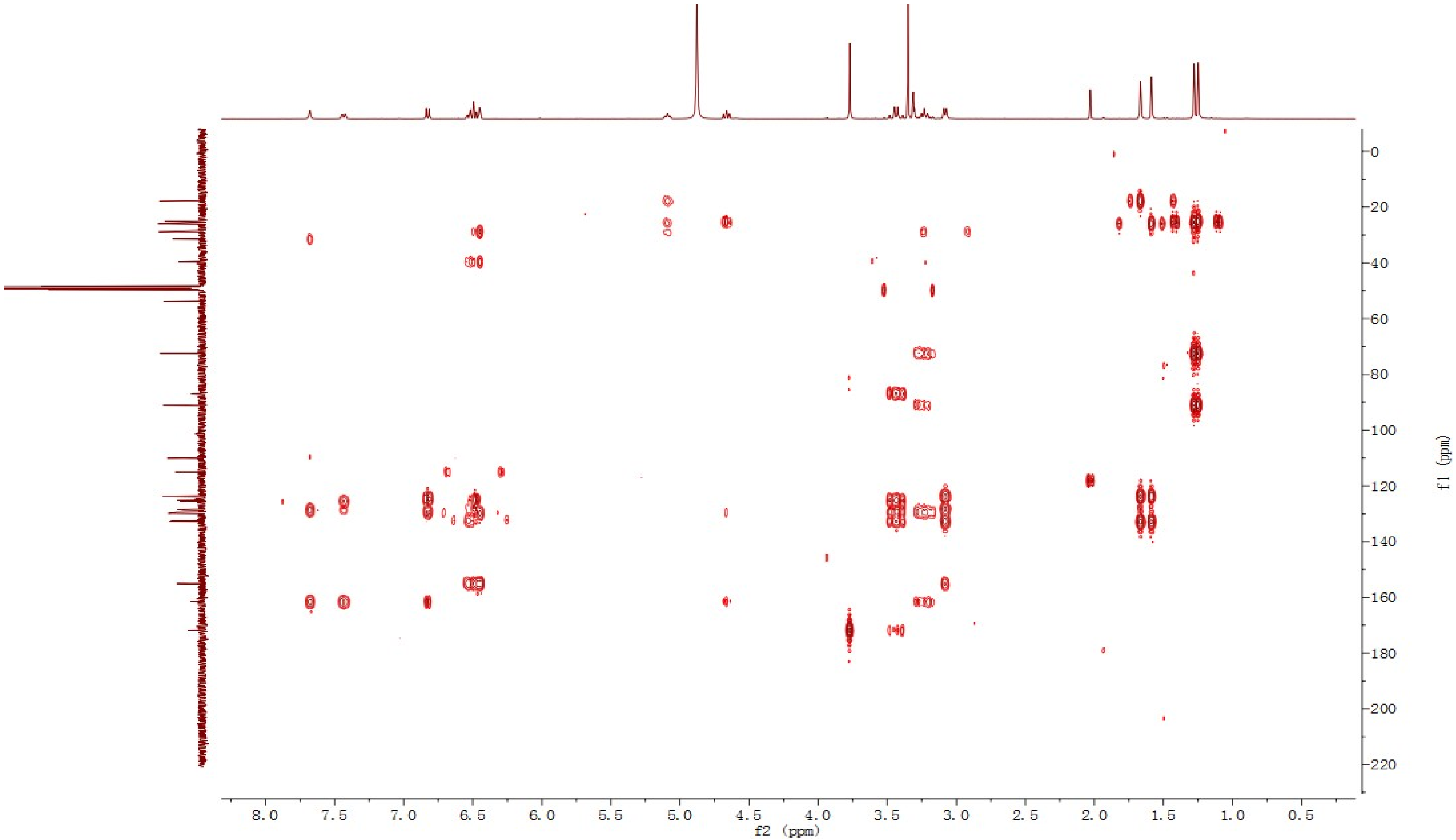

Figure S25. HMBC spectrum of compound **3** (Recorded in methanol-d_4)

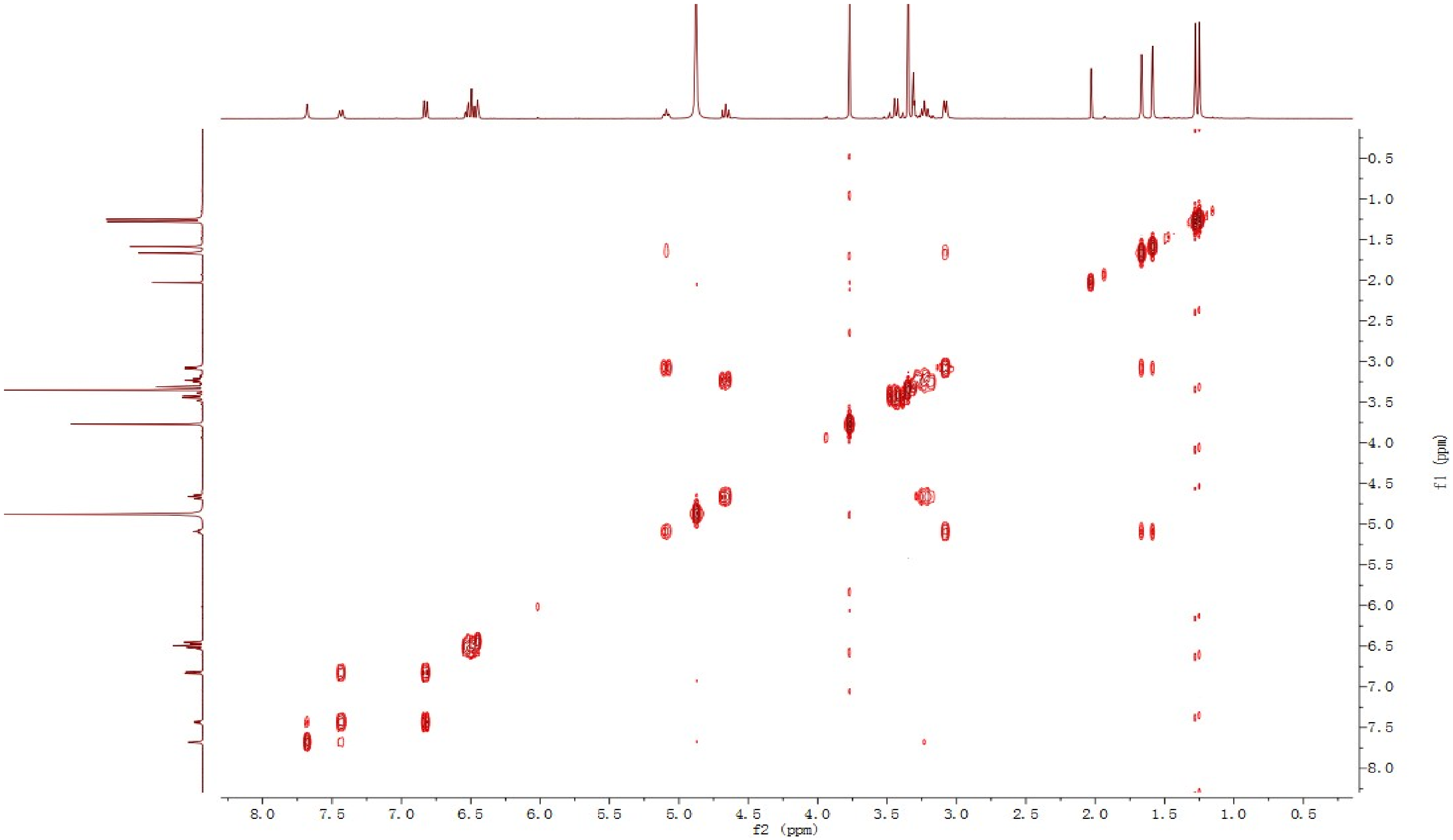

Figure S26. ^{1}H–^{1}H COSY spectrum of compound **3** (Recorded in methanol-d_4)

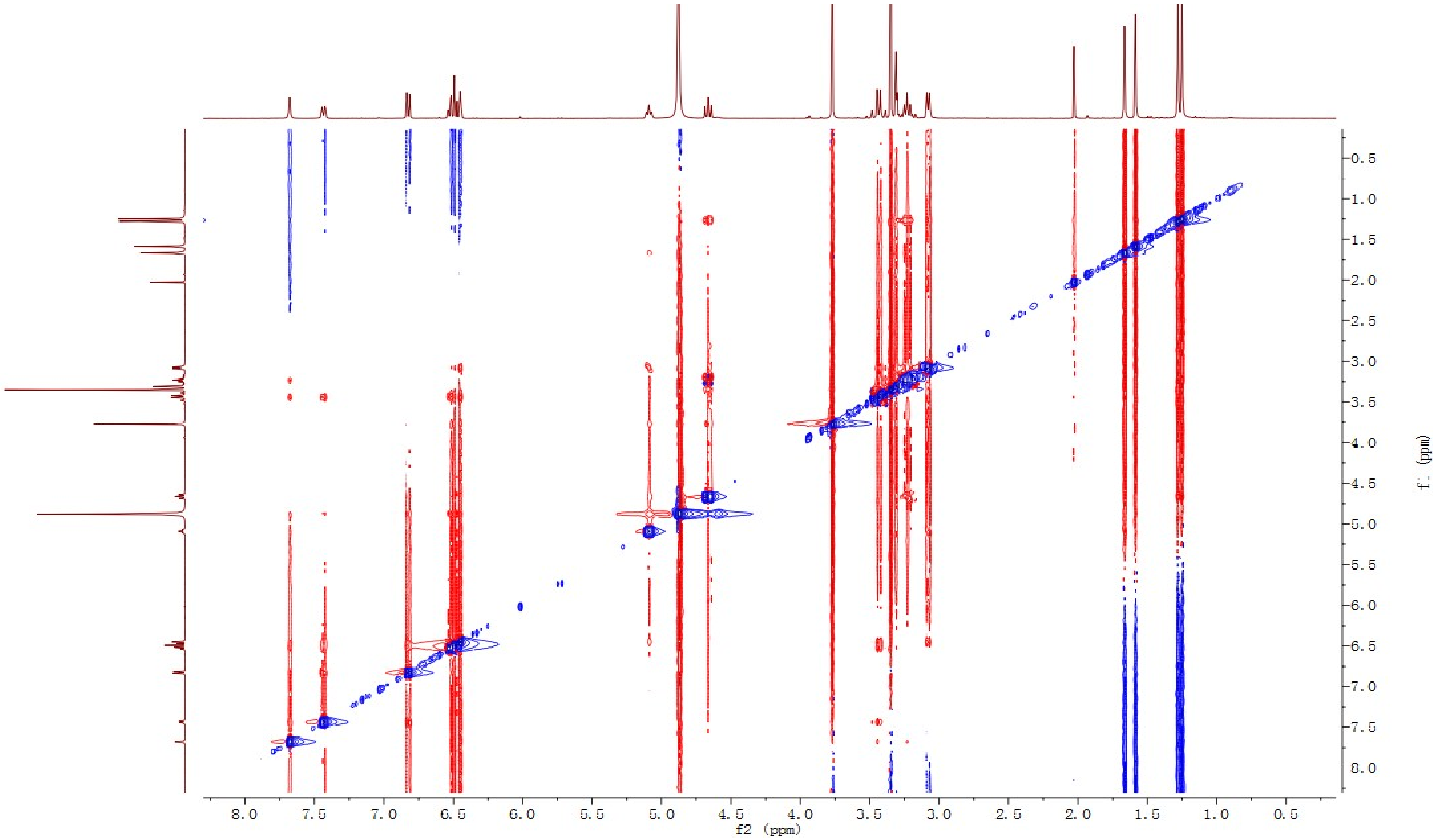

Figure S27. NOESY spectrum of compound **3** (Recorded in methanol-d_4)

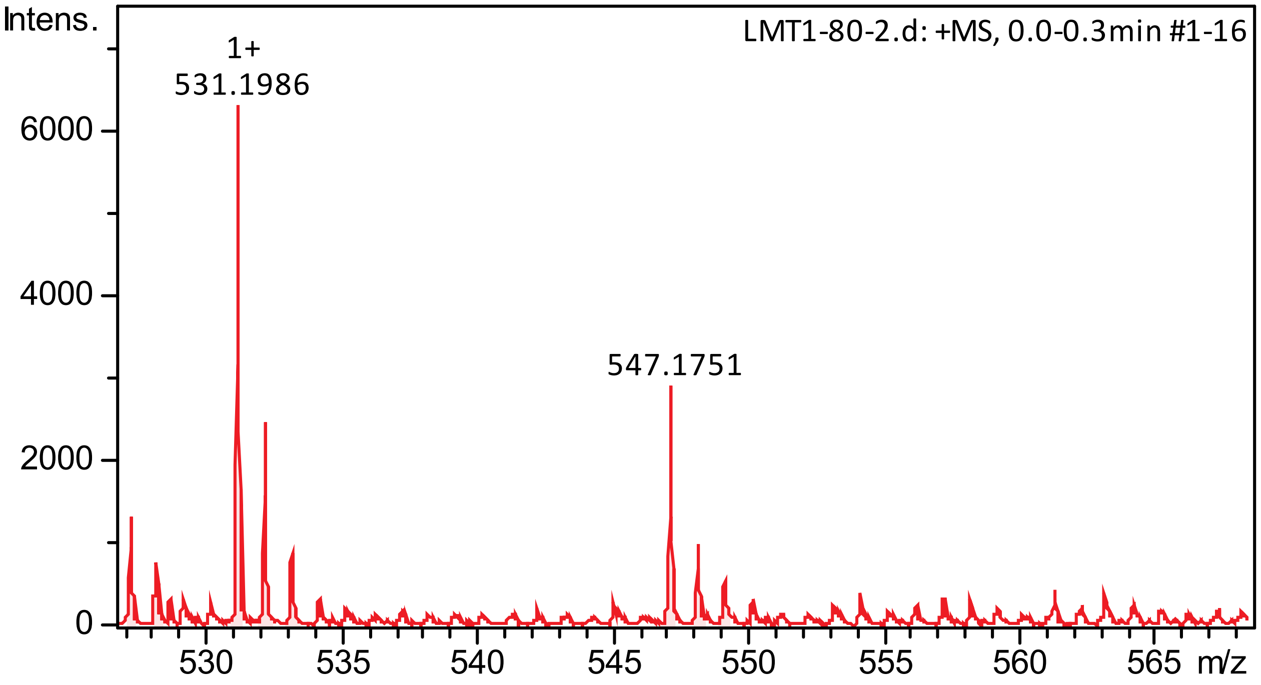

Figure S28. HRESIMS spectrum of compound **3**

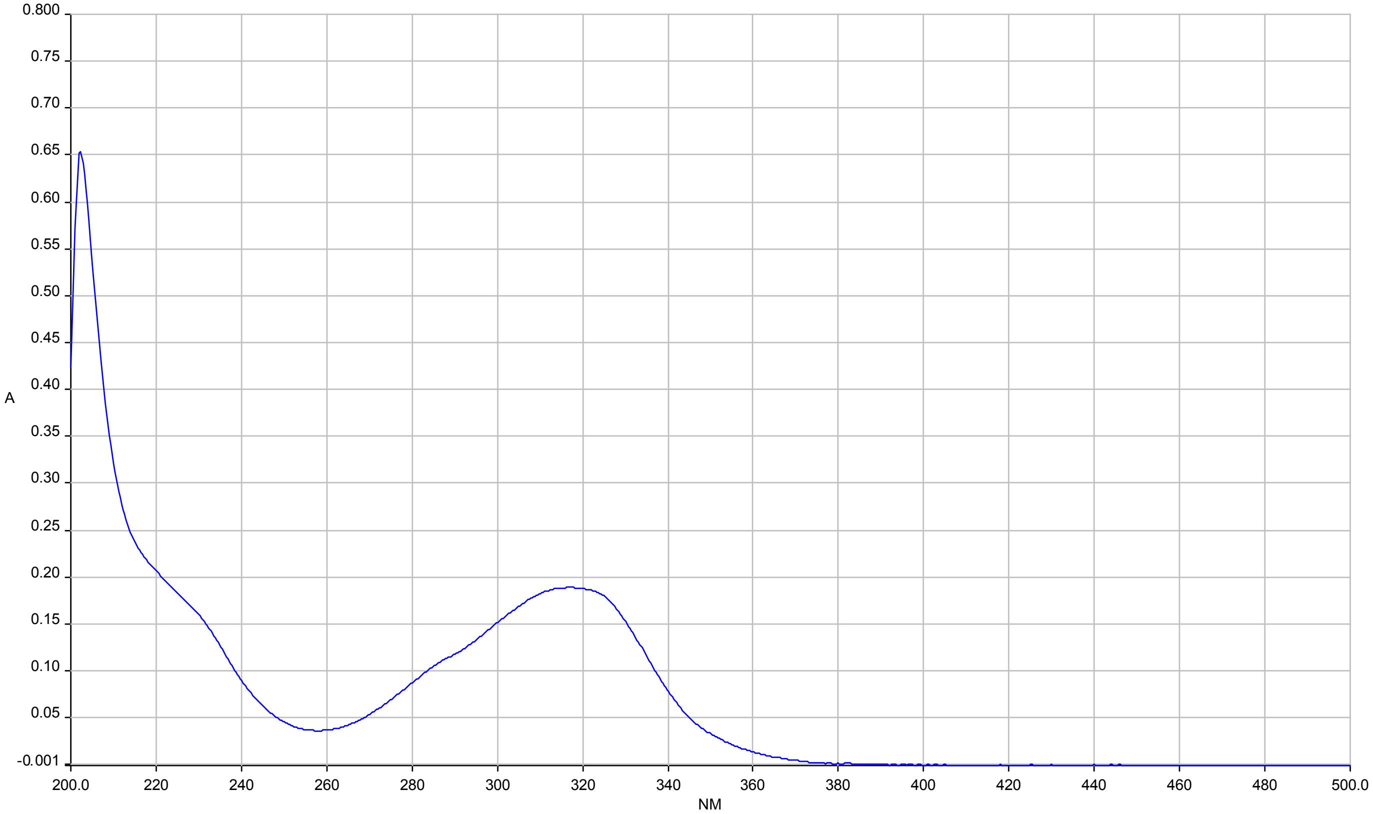

Figure S29. UV spectrum of compound **3**

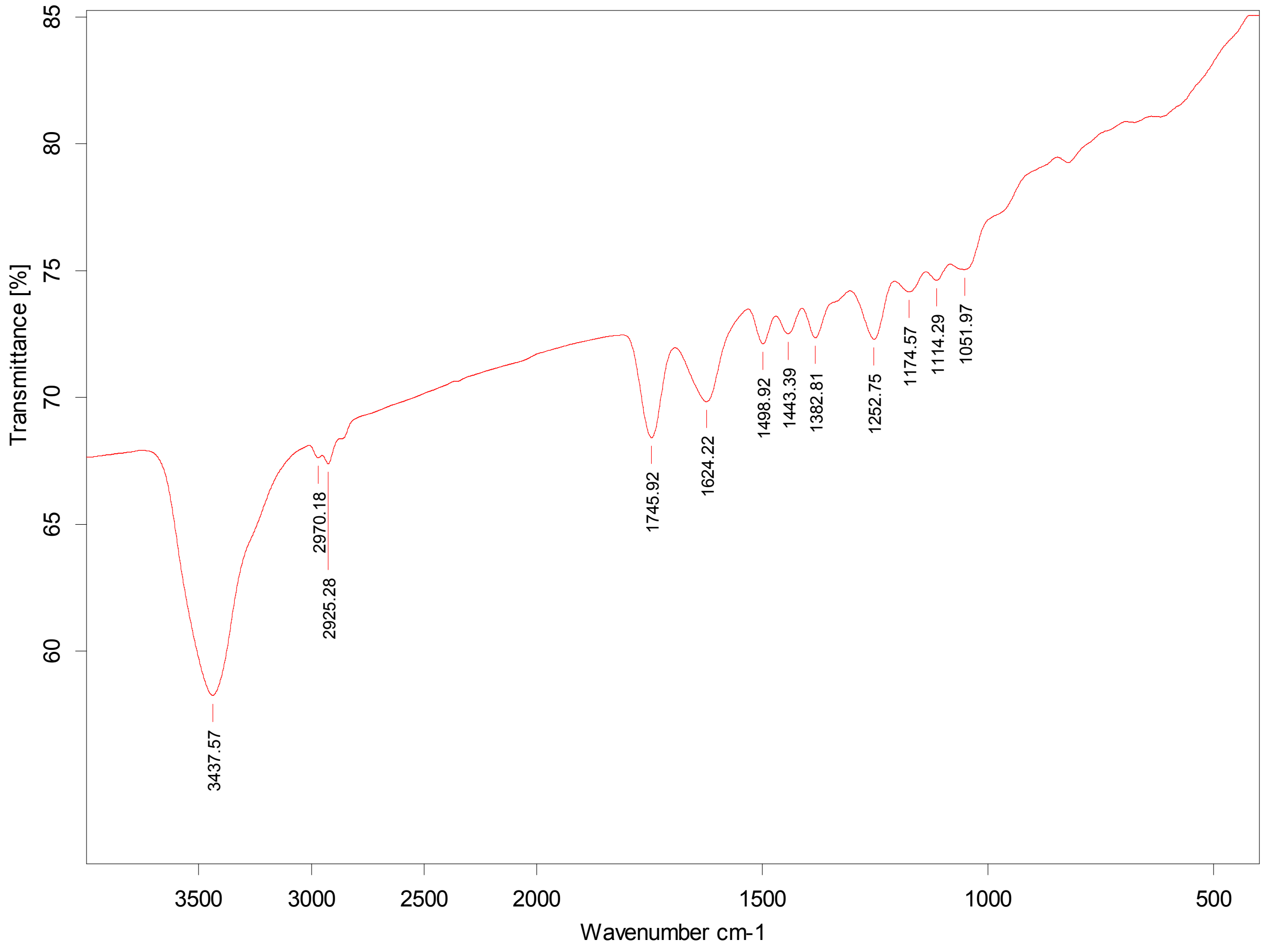

Figure S30. IR spectrum of compound **3**

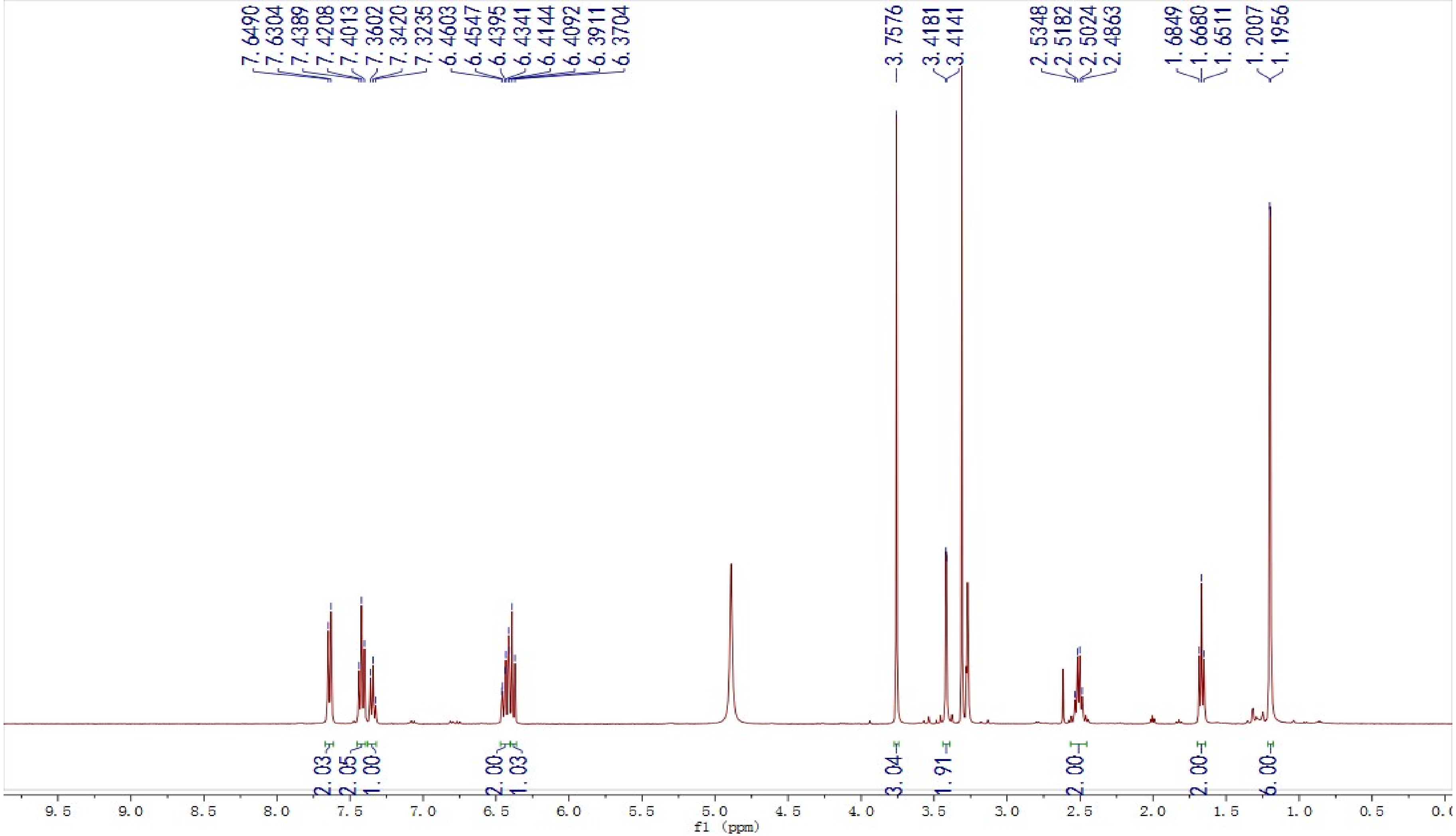

Figure S31. ^{1}H NMR spectrum of compound **4** (Recorded in methanol-d_4)

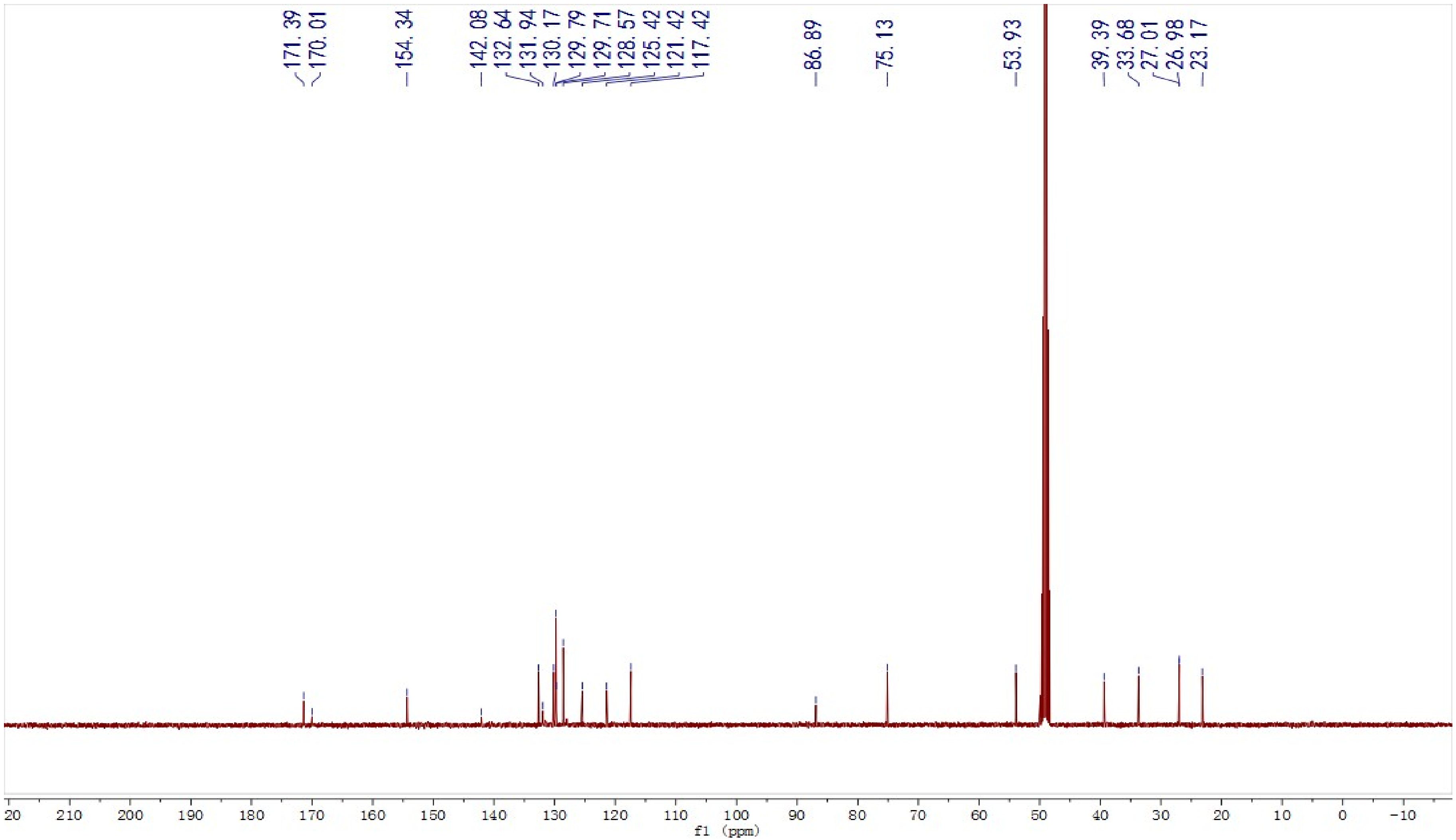

Figure S32. ^{13}C NMR spectrum of compound **4** (Recorded in methanol-d_4)

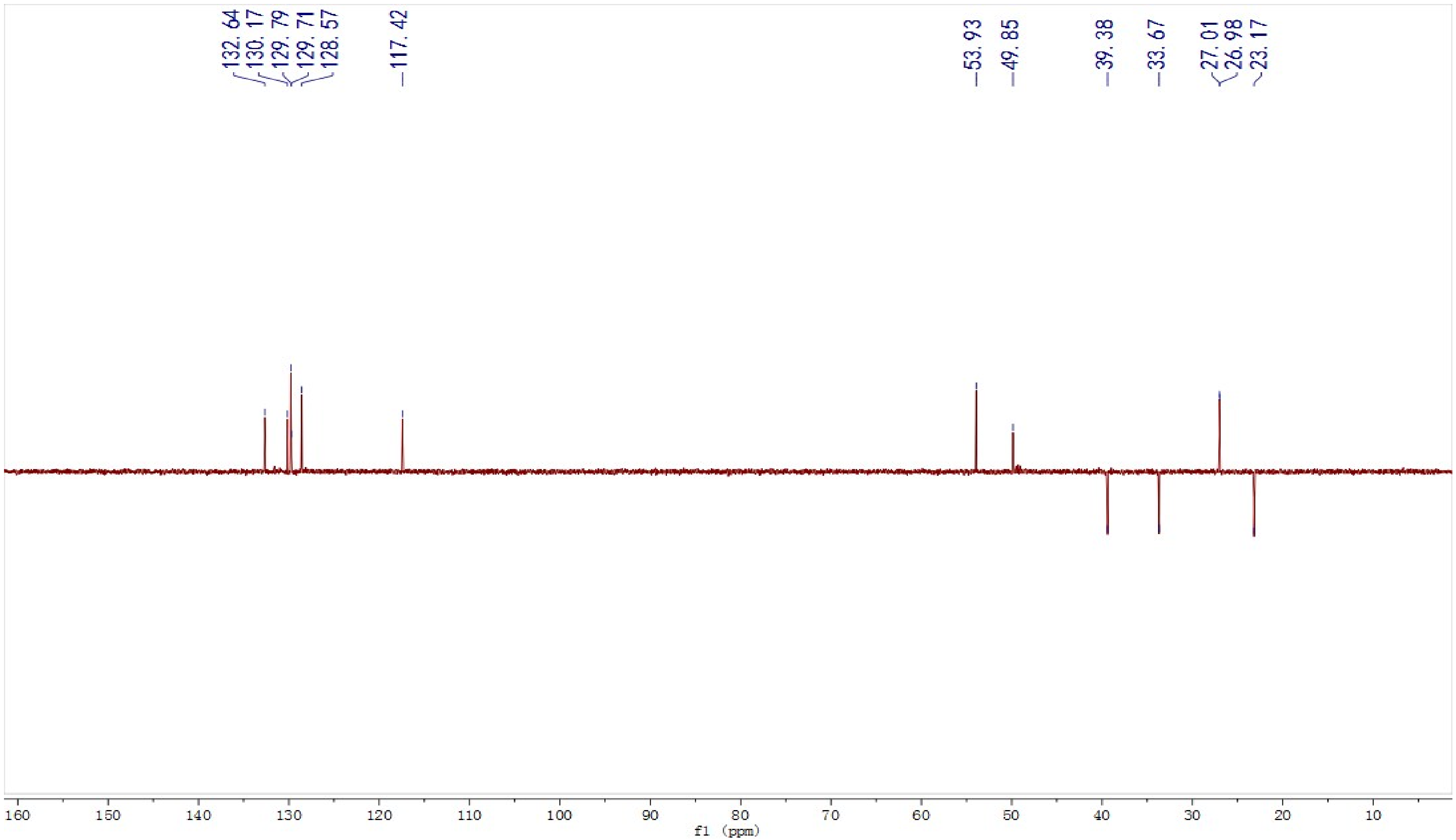

Figure S33. DEPT spectrum of compound **4** (Recorded in methanol-d_4)

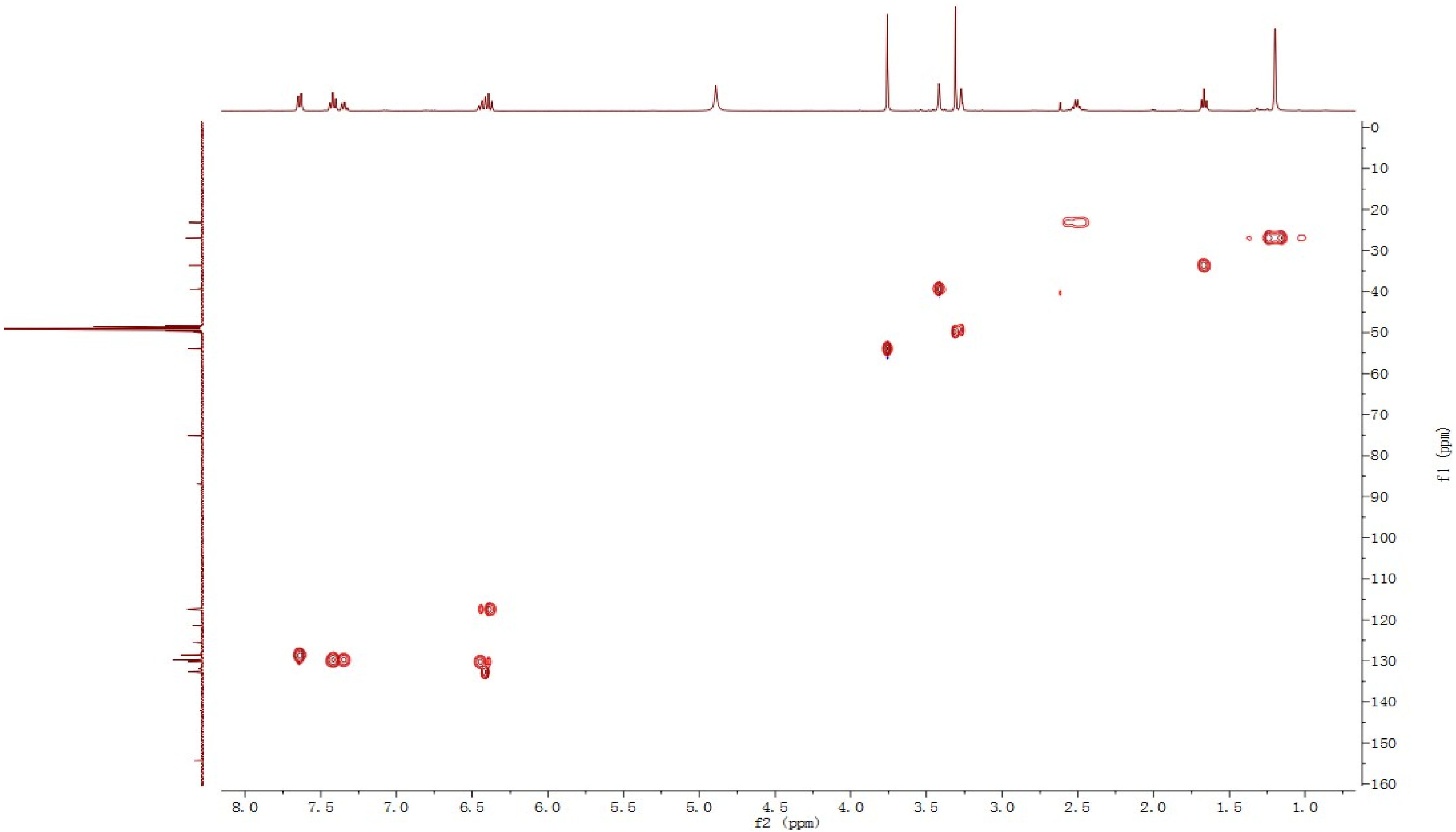

Figure S34. HSQC spectrum of compound **4** (Recorded in methanol-d_4)

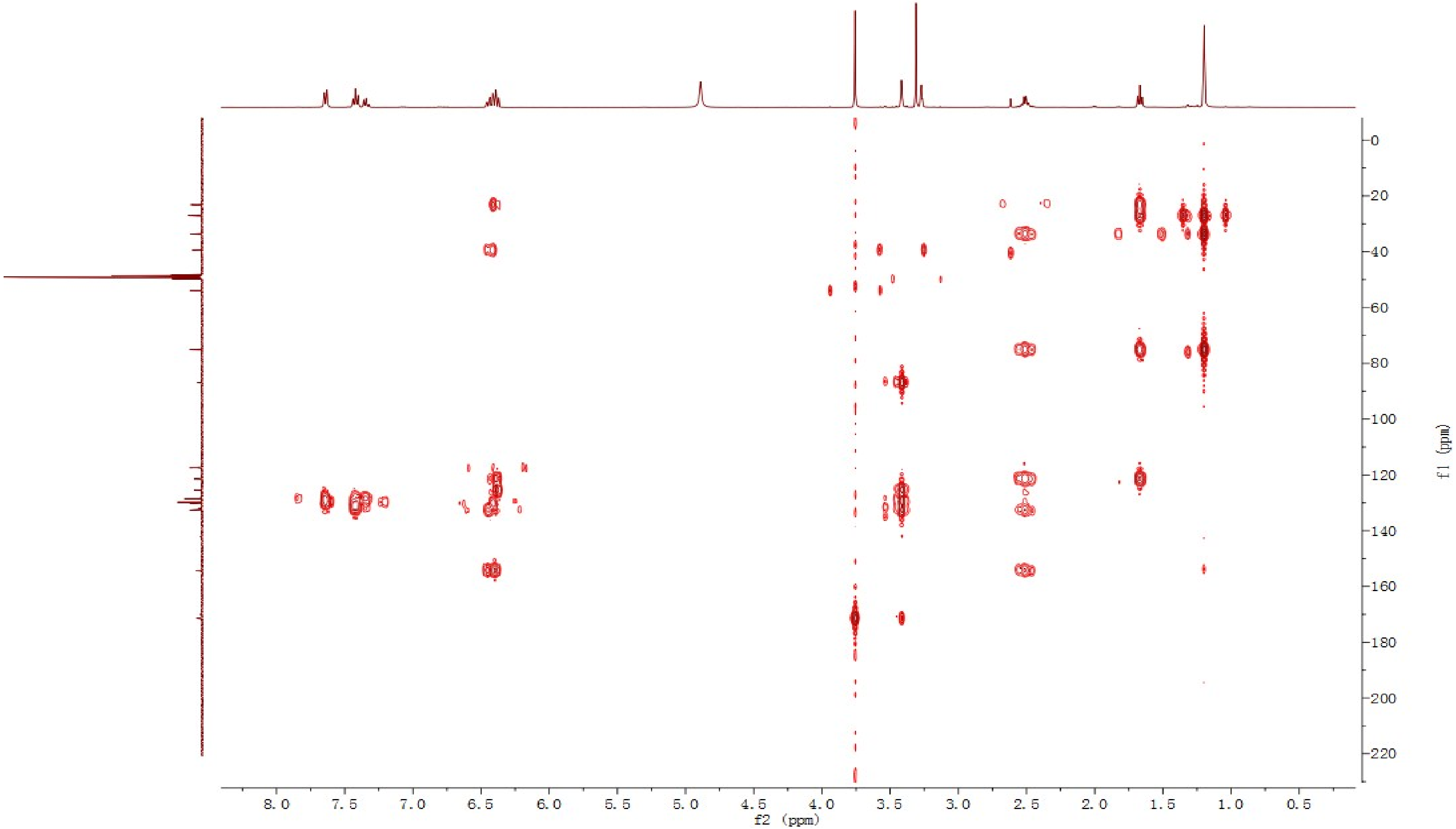

Figure S35. HMBC spectrum of compound **4** (Recorded in methanol-d_4)

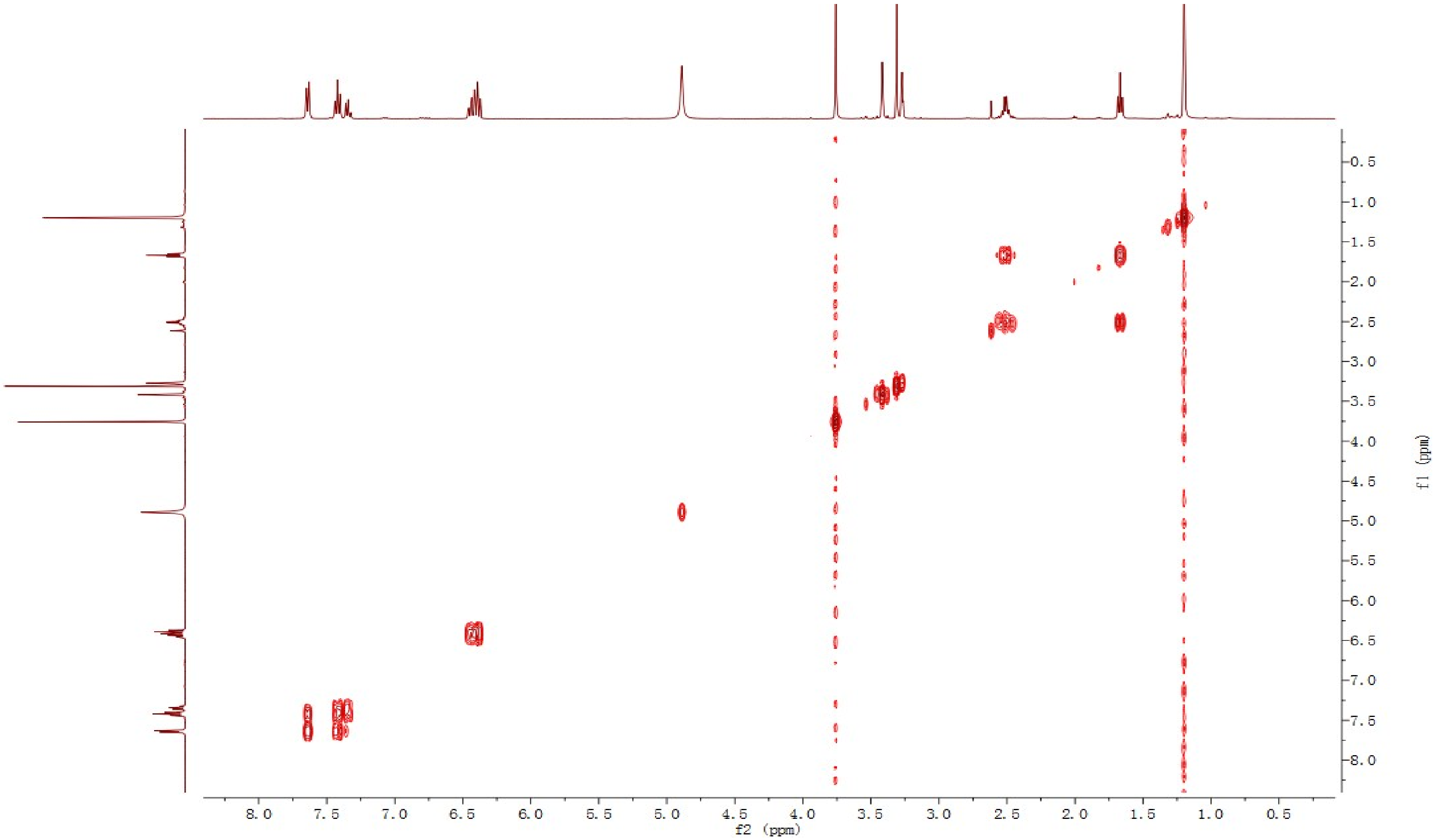

Figure S36. ^{1}H–^{1}H COSY spectrum of compound **4** (Recorded in methanol-d_4)

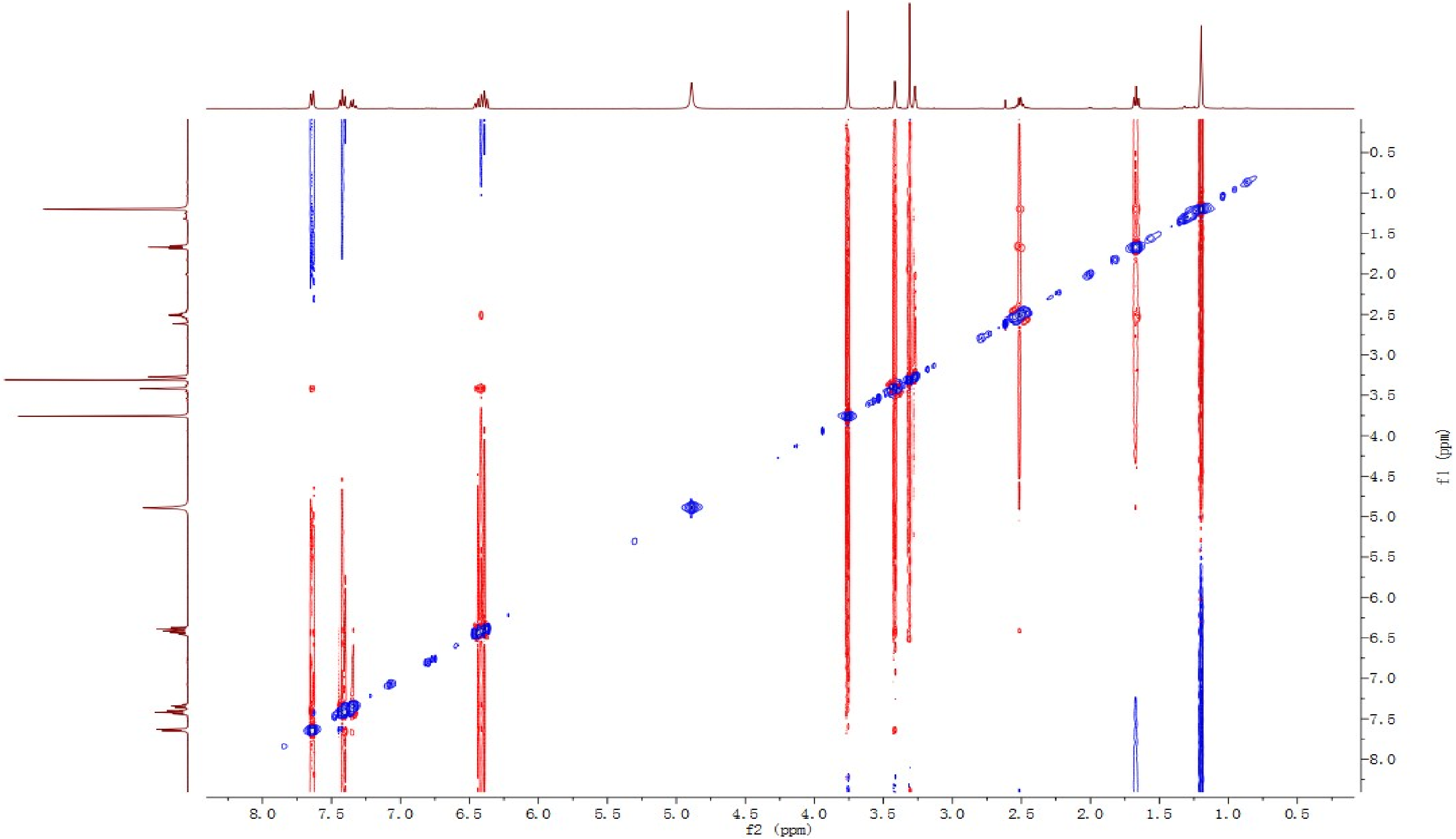

Figure S37. NOESY spectrum of compound **4** (Recorded in methanol-d_4)

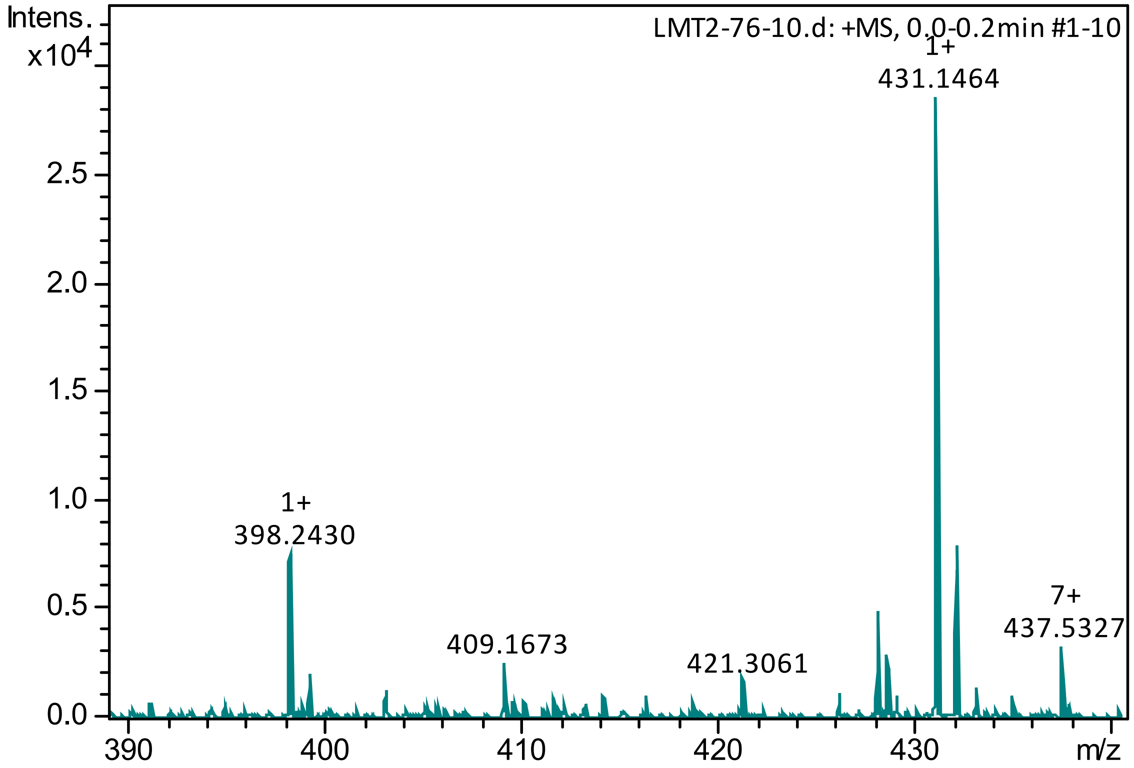

Figure S38. HRESIMS spectrum of compound **4**

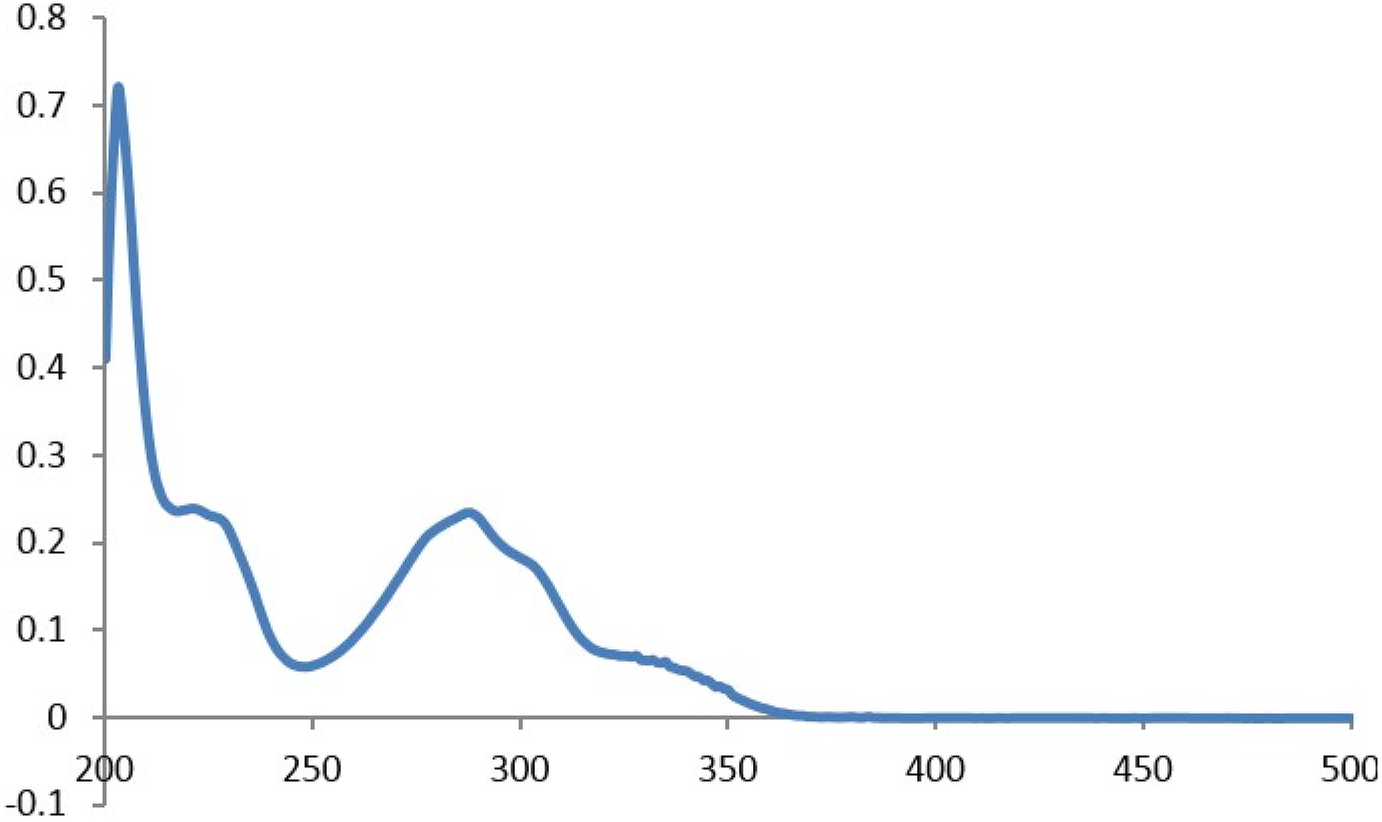

Figure S39. UV spectrum of compound **4**

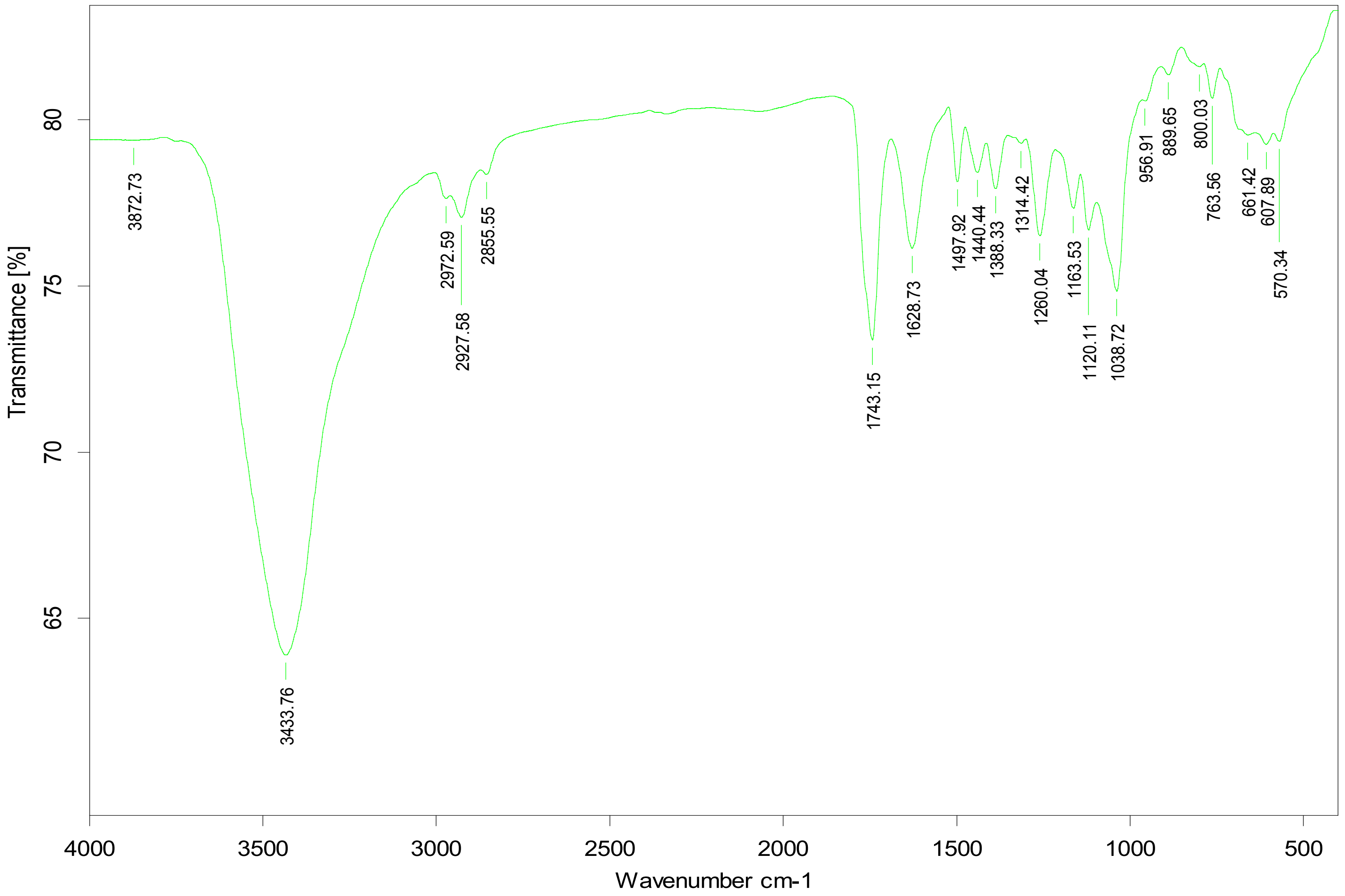

Figure S40. IR spectrum of compound **4**

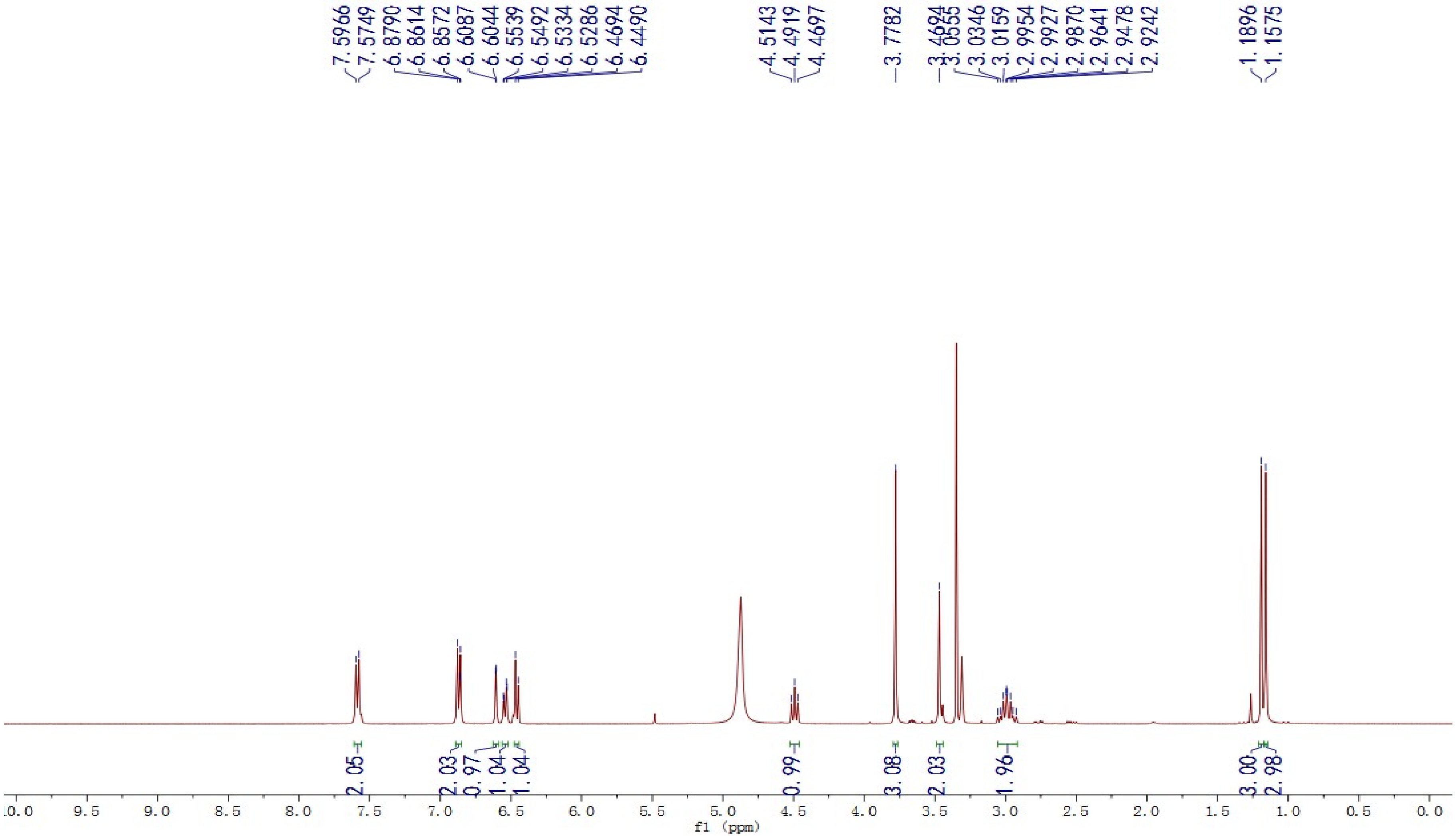

Figure S41. ^{1}H NMR spectrum of compound **5** (Recorded in methanol-d_4)

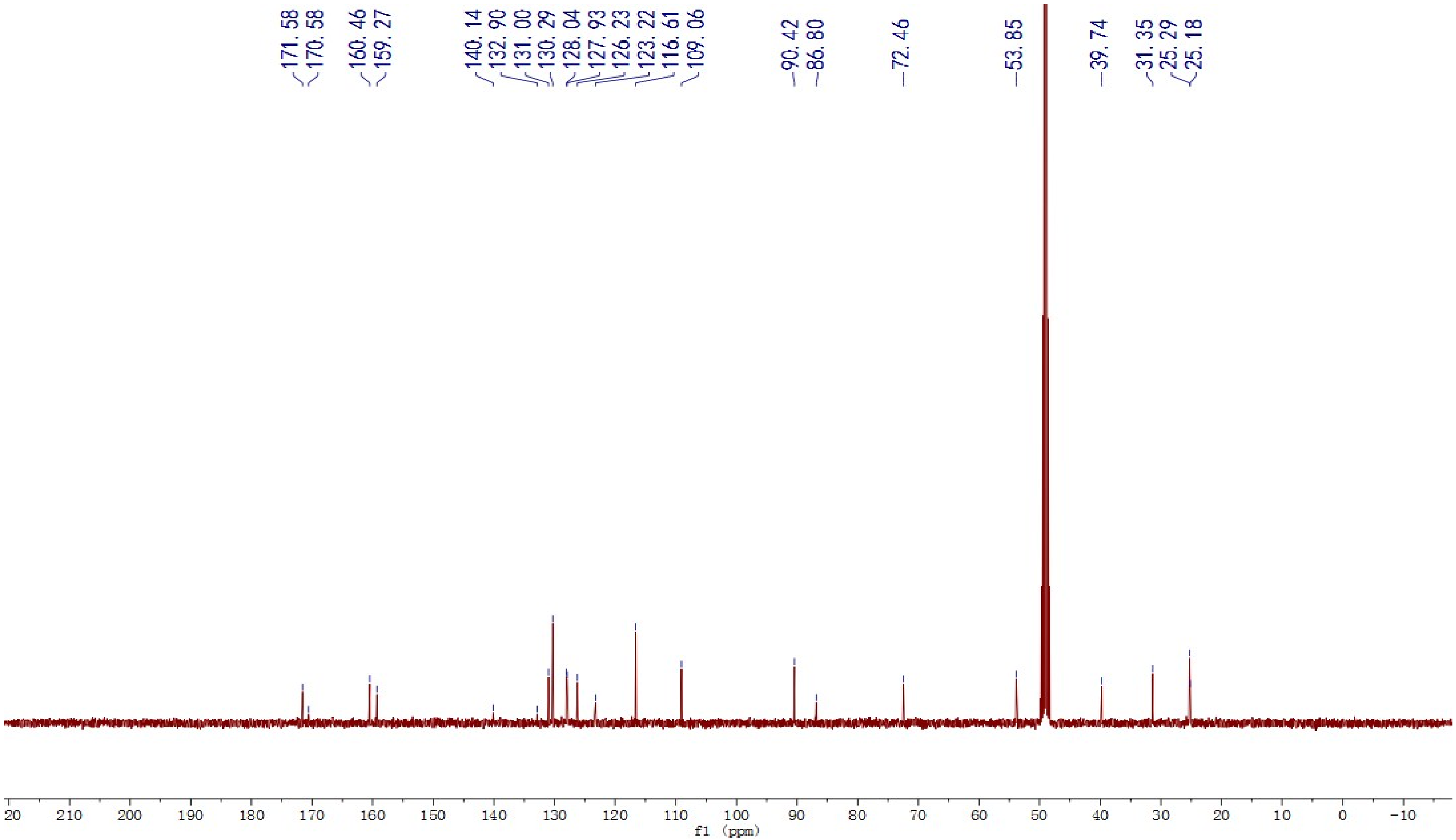

Figure S42. ^{13}C NMR spectrum of compound **5** (Recorded in methanol-d_4)

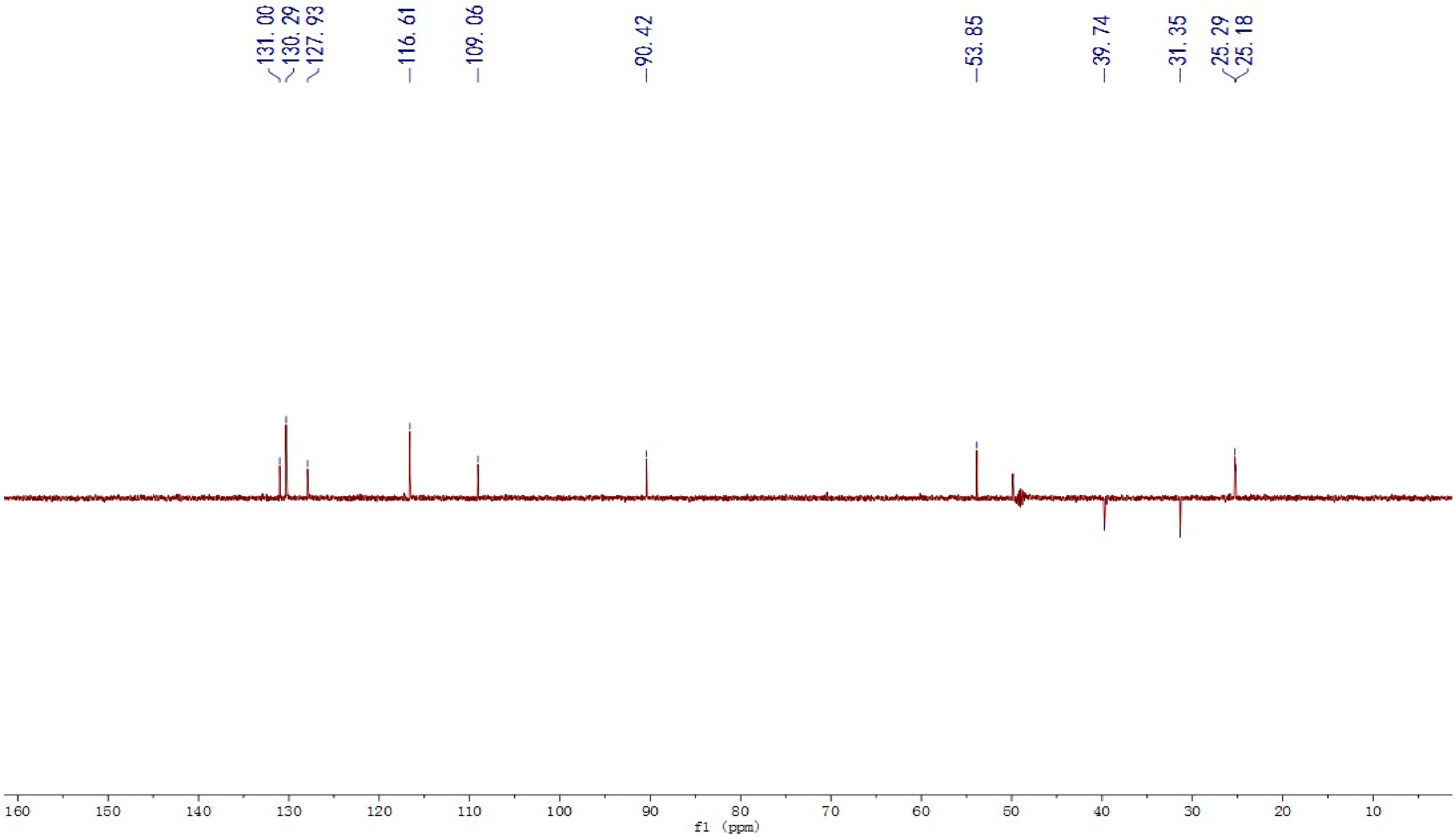

Figure S43. DEPT spectrum of compound **5** (Recorded in methanol-d_4)

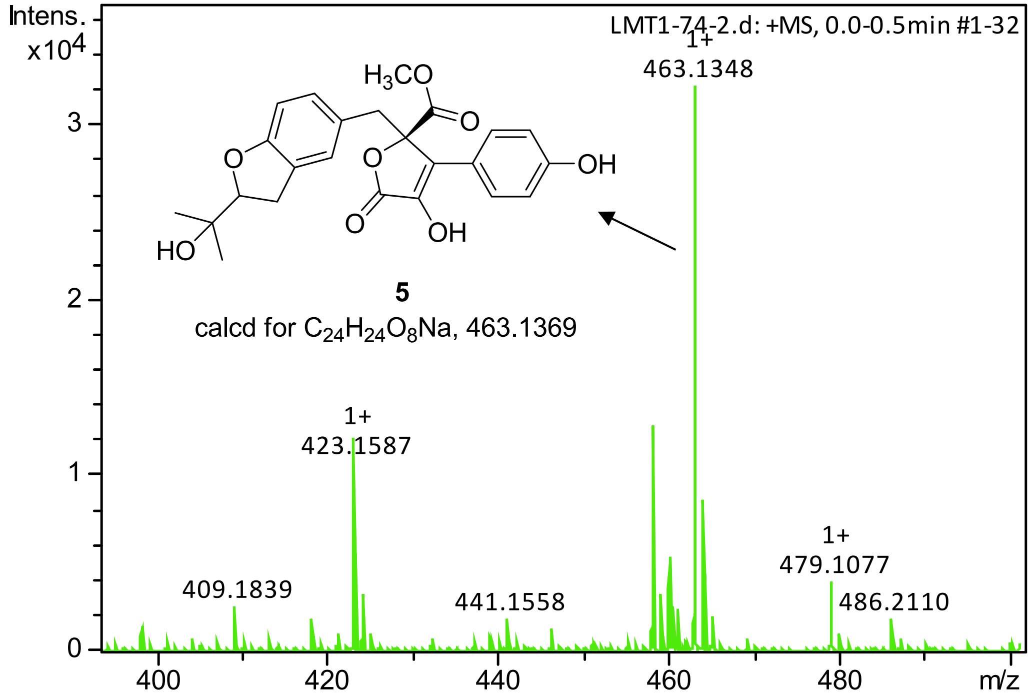

Figure S44. HRESIMS spectrum of compound **5**

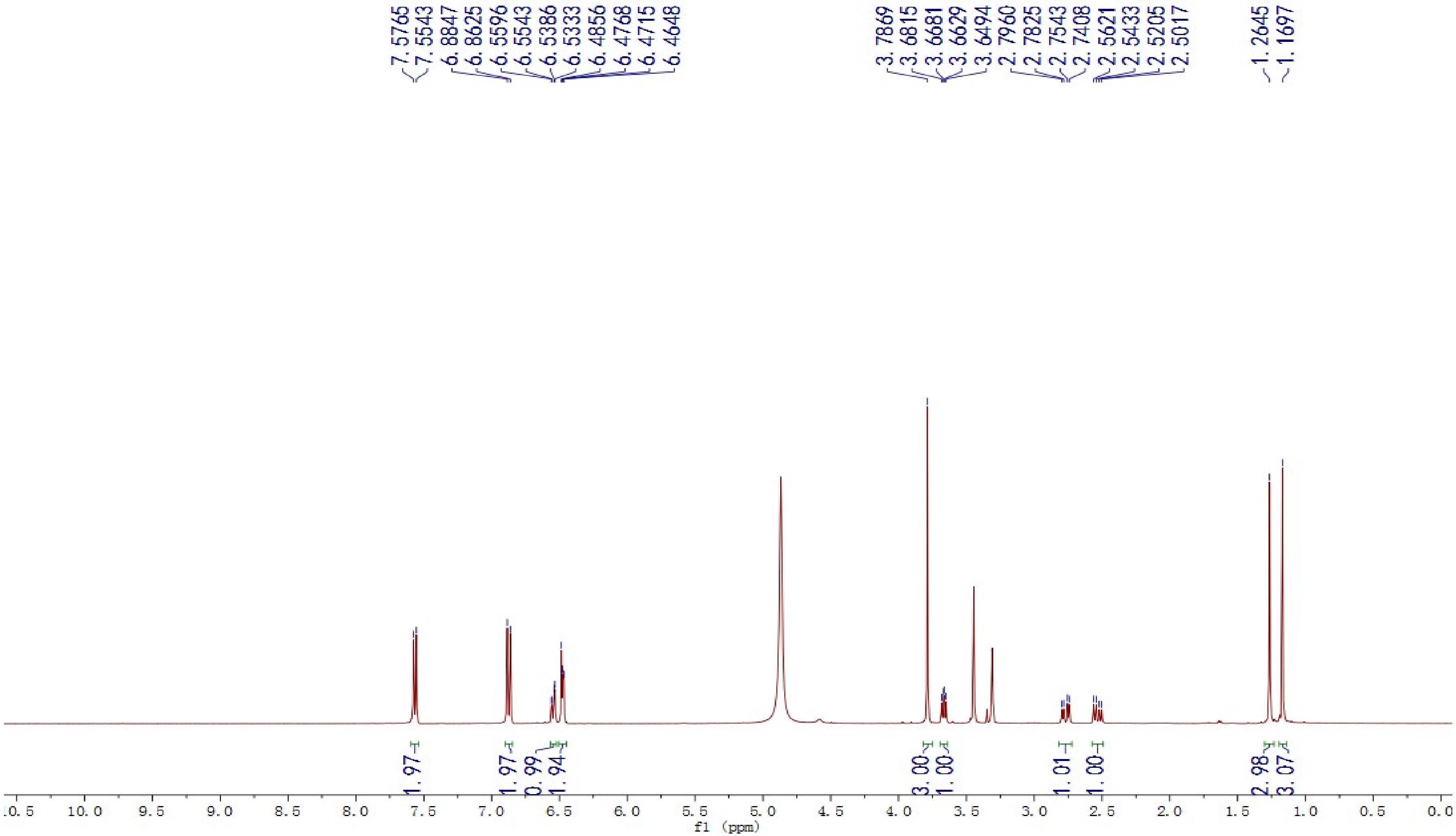

Figure S45. ^{1}H NMR spectrum of compound **7** (Recorded in methanol-d_4)

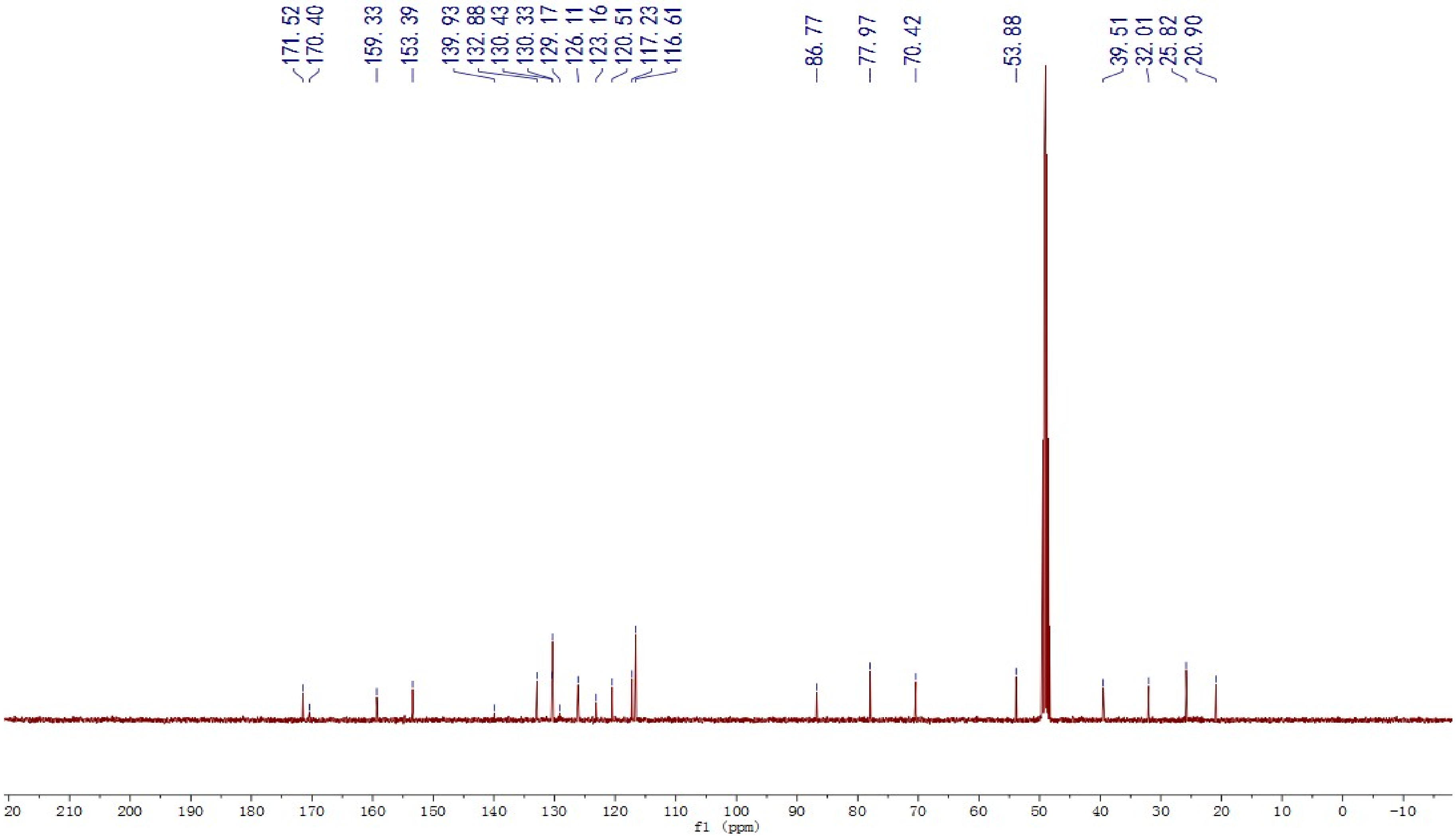

Figure S46. ^{13}C NMR spectrum of compound **7** (Recorded in methanol-d_4)

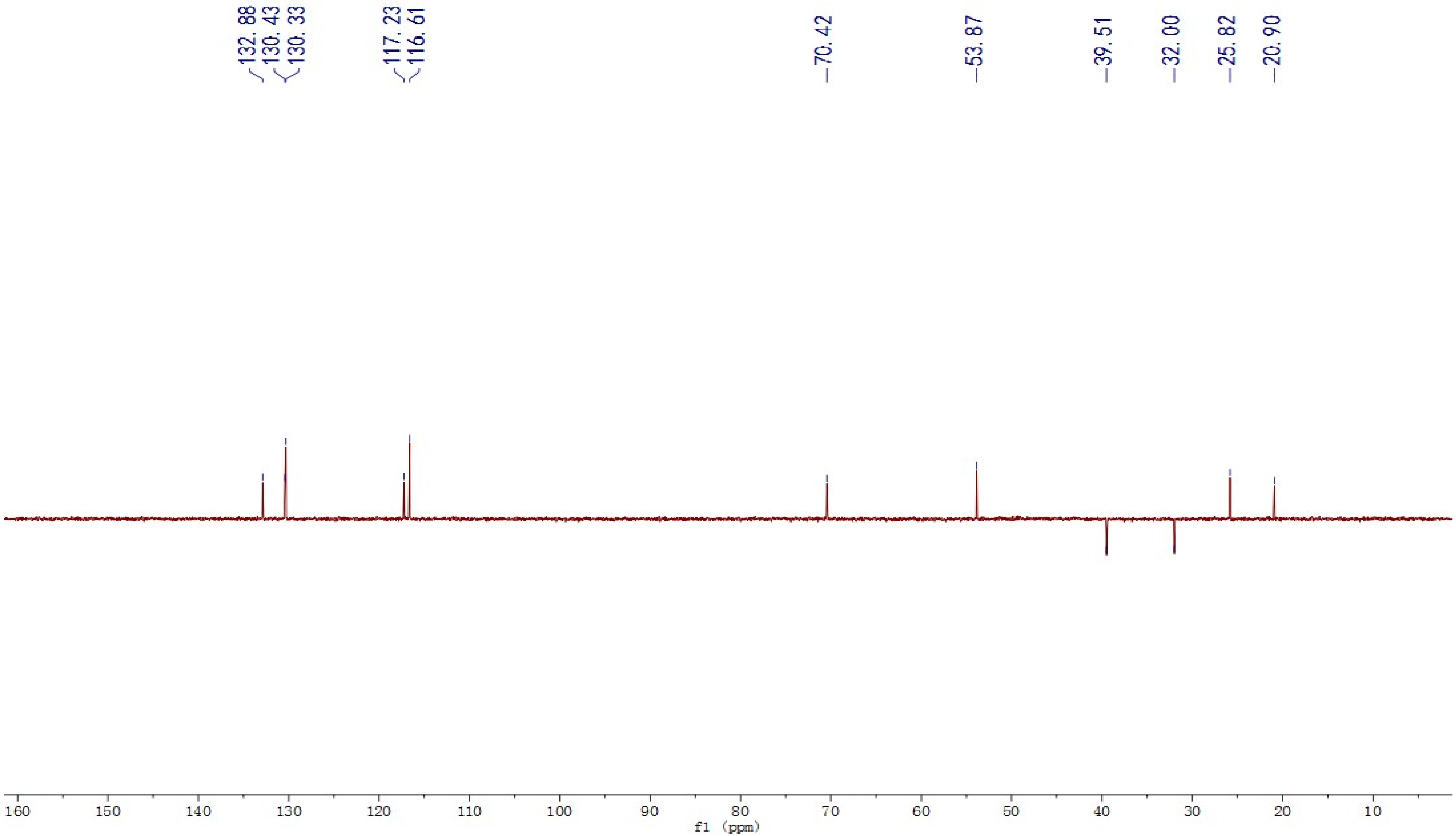

Figure S47. DEPT spectrum of compound **7** (Recorded in methanol-d_4)

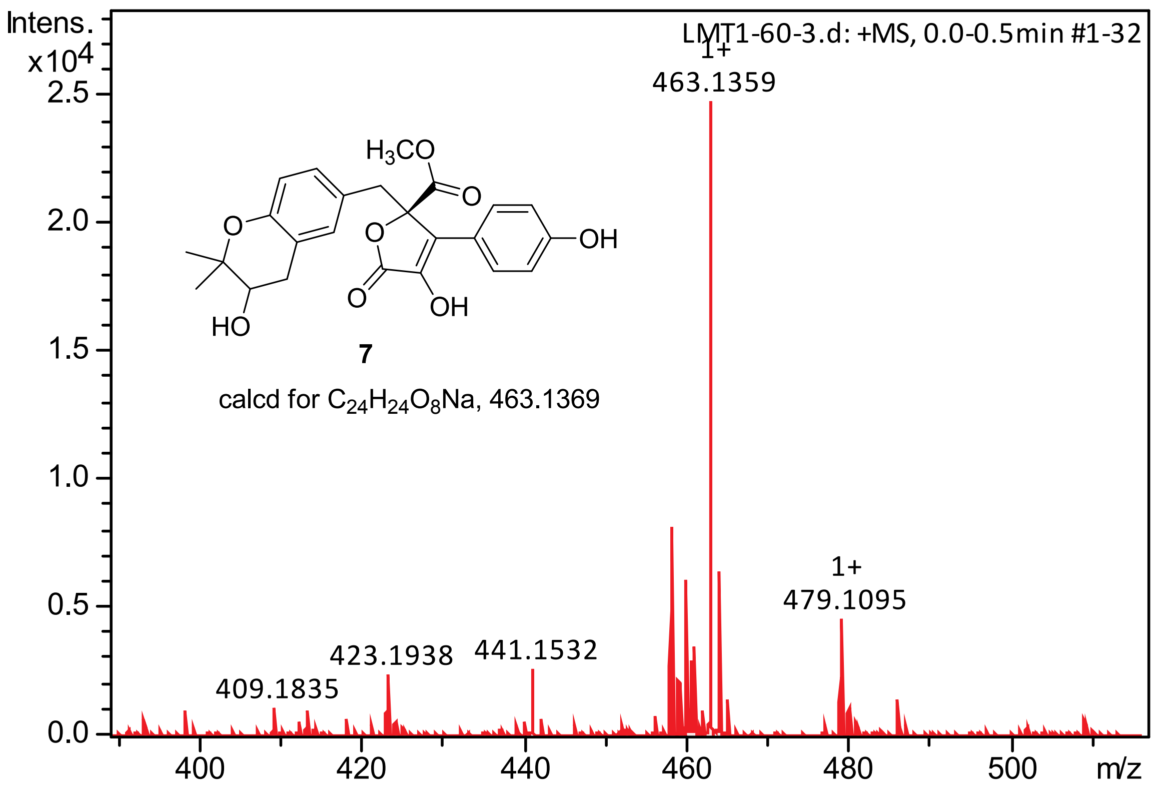

Figure S48. HRESIMS spectrum of compound **7**

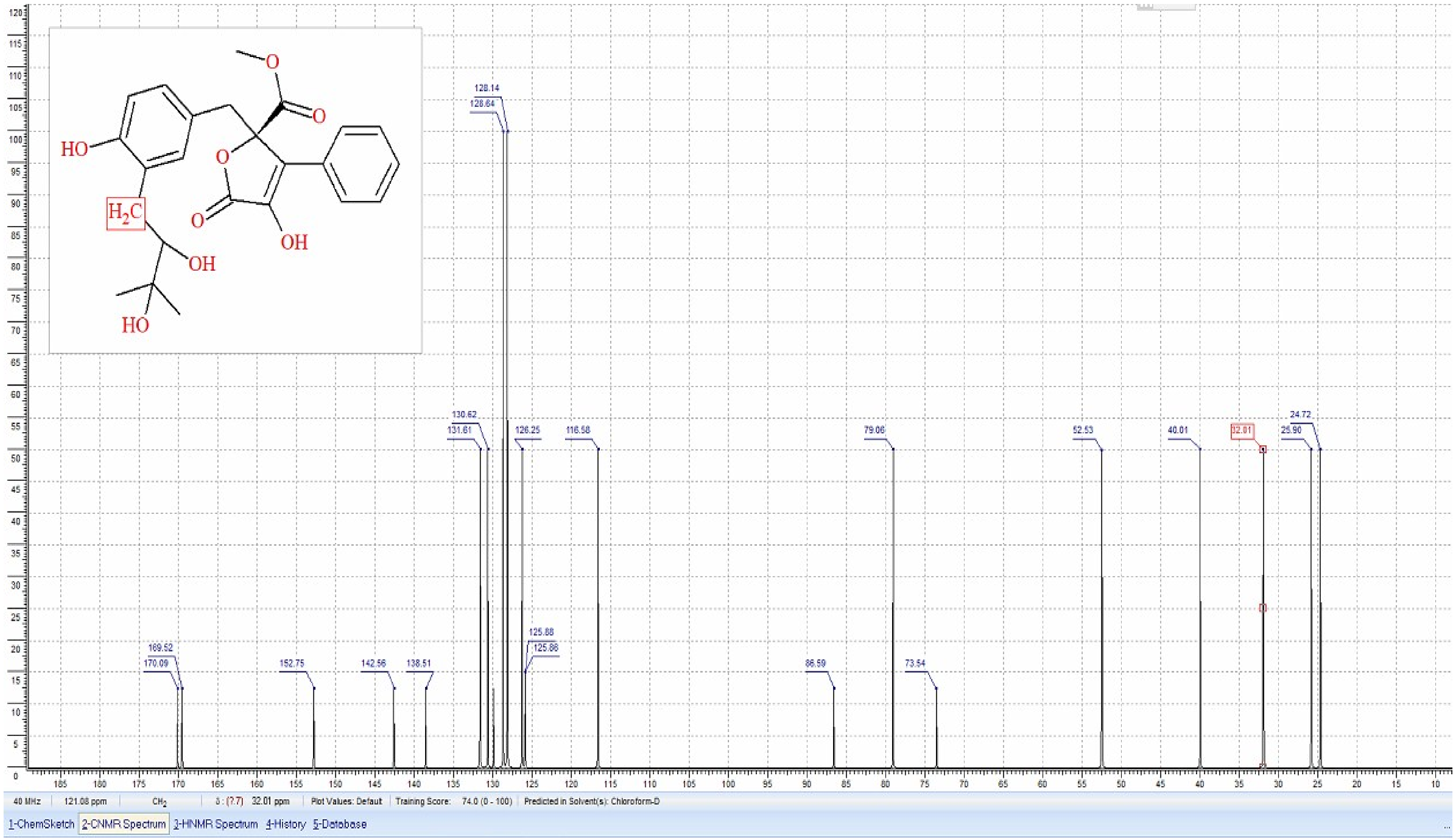

Figure S49. ^{13}C NMR prediction at C-7″ for compound **1** by ACD/Labs software

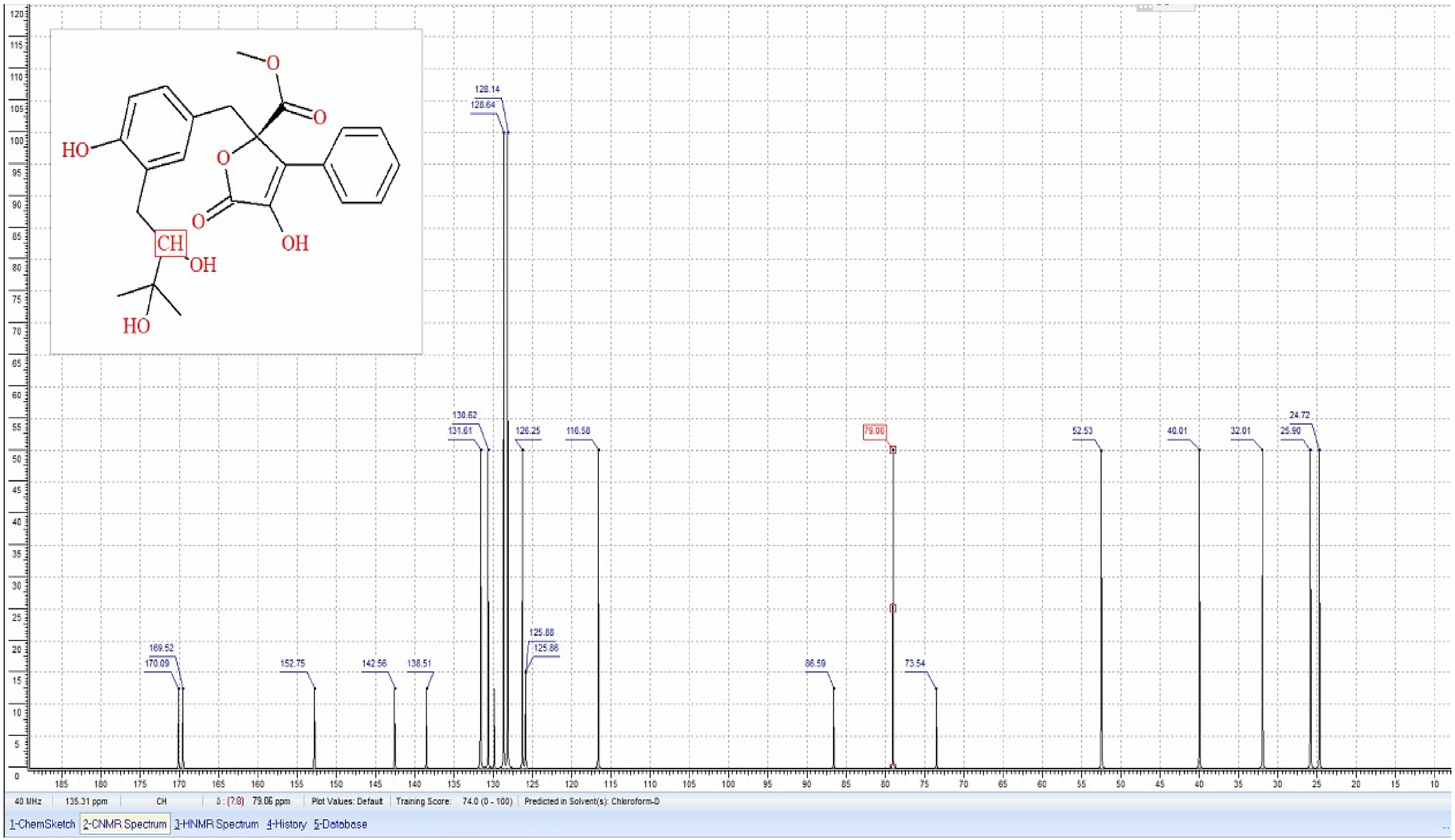

Figure S50. ^{13}C NMR prediction at C-8″ for compound **1** by ACD/Labs software

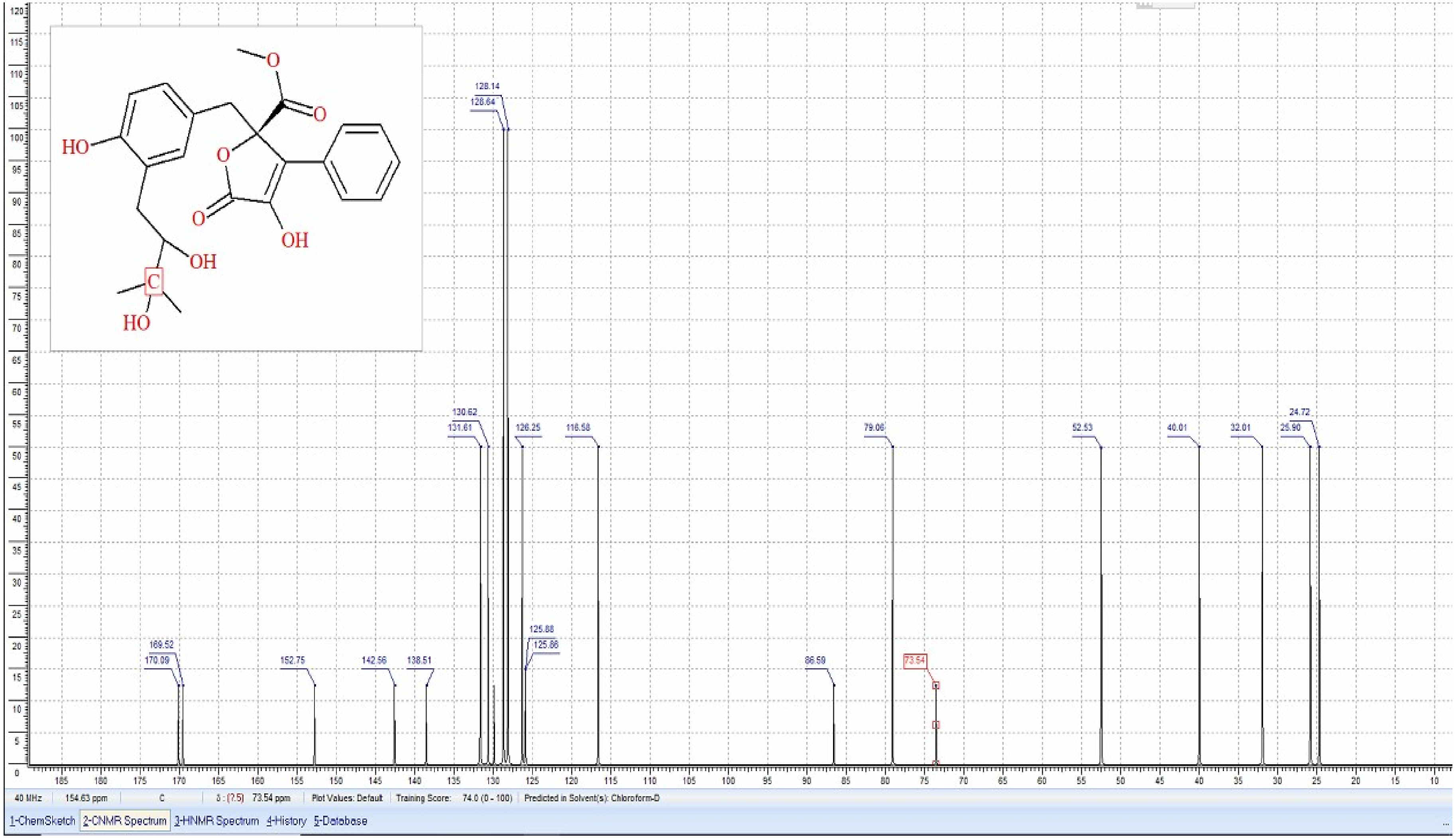

Figure S51. ^{13}C NMR prediction at C-9″ for compound **1** by ACD/Labs software

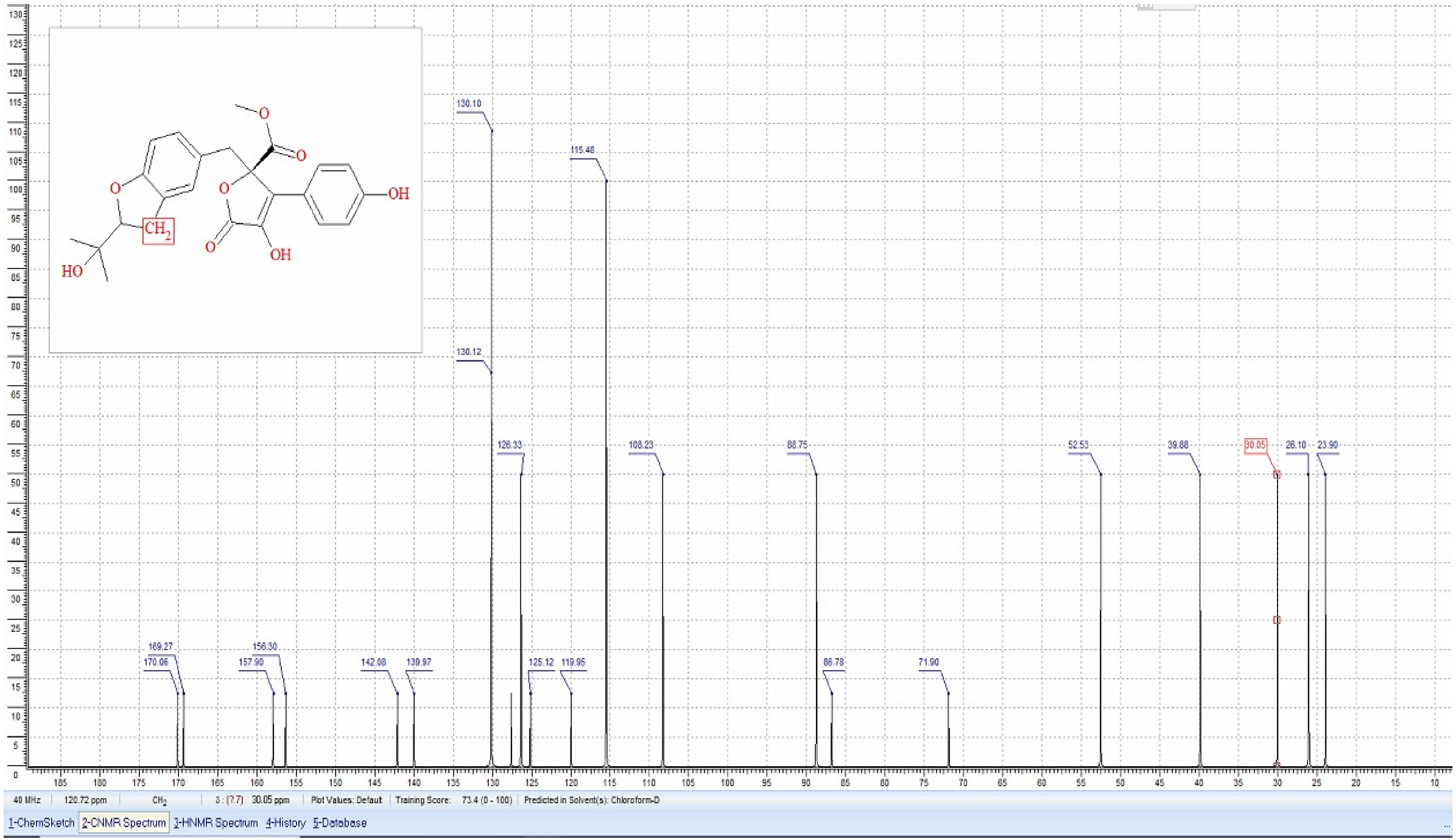

Figure S52. ^{13}C NMR prediction at C-7″ for compound **5** by ACD/Labs software

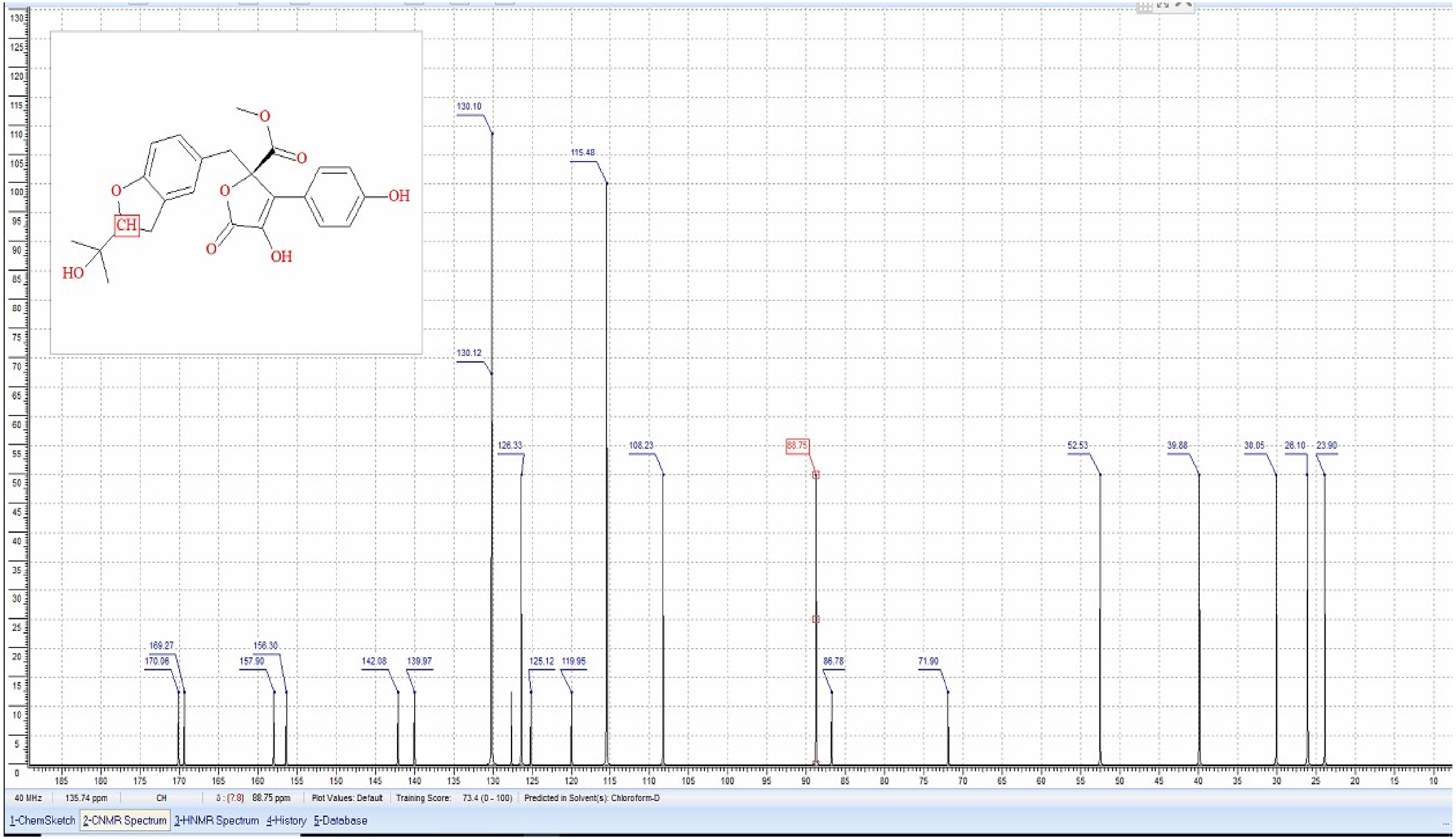

Figure S53. ^{13}C NMR prediction at C-8″ for compound **5** by ACD/Labs software

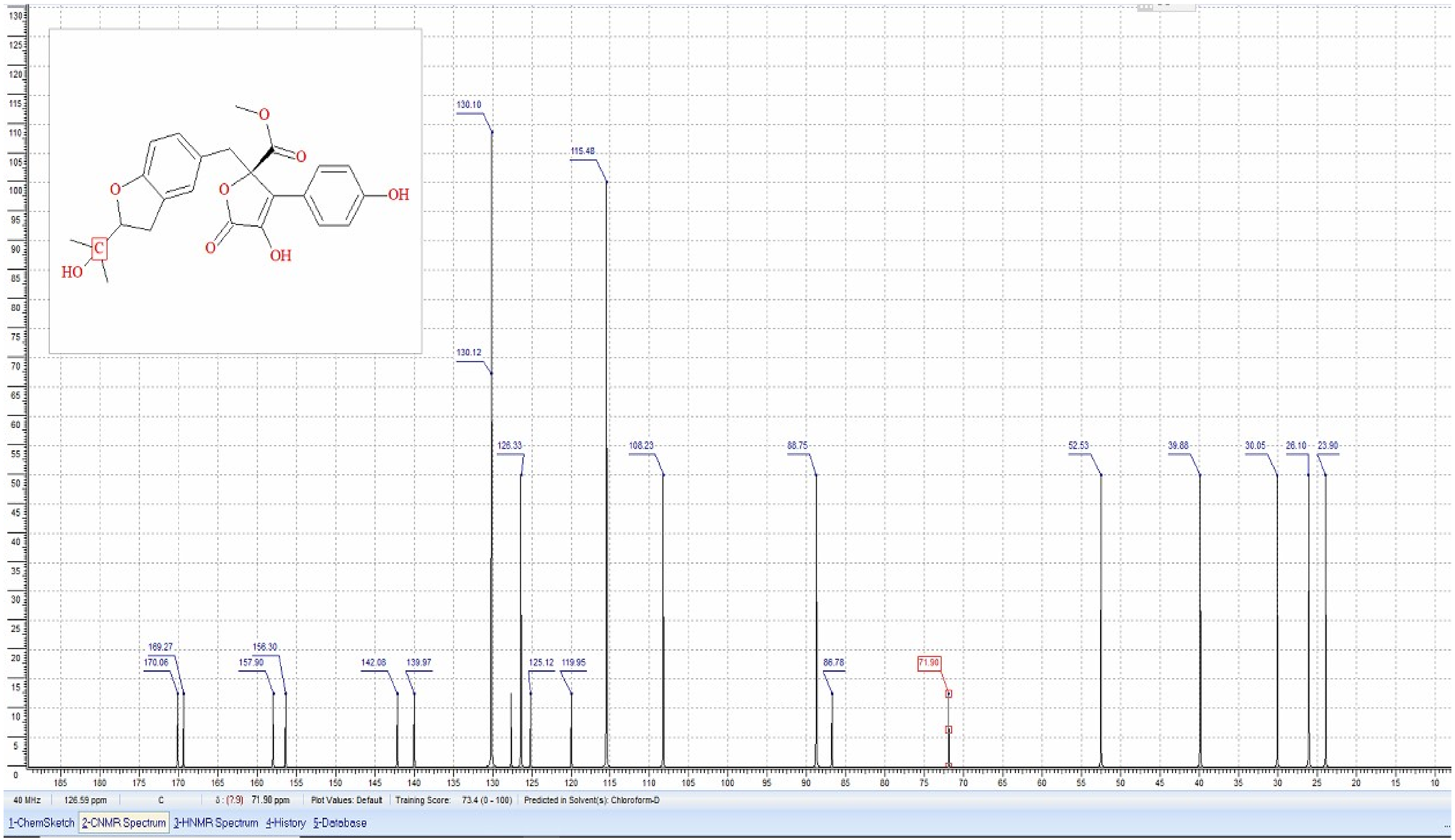

Figure S54. ^{13}C NMR prediction at C-9″ for compound **5** by ACD/Labs software

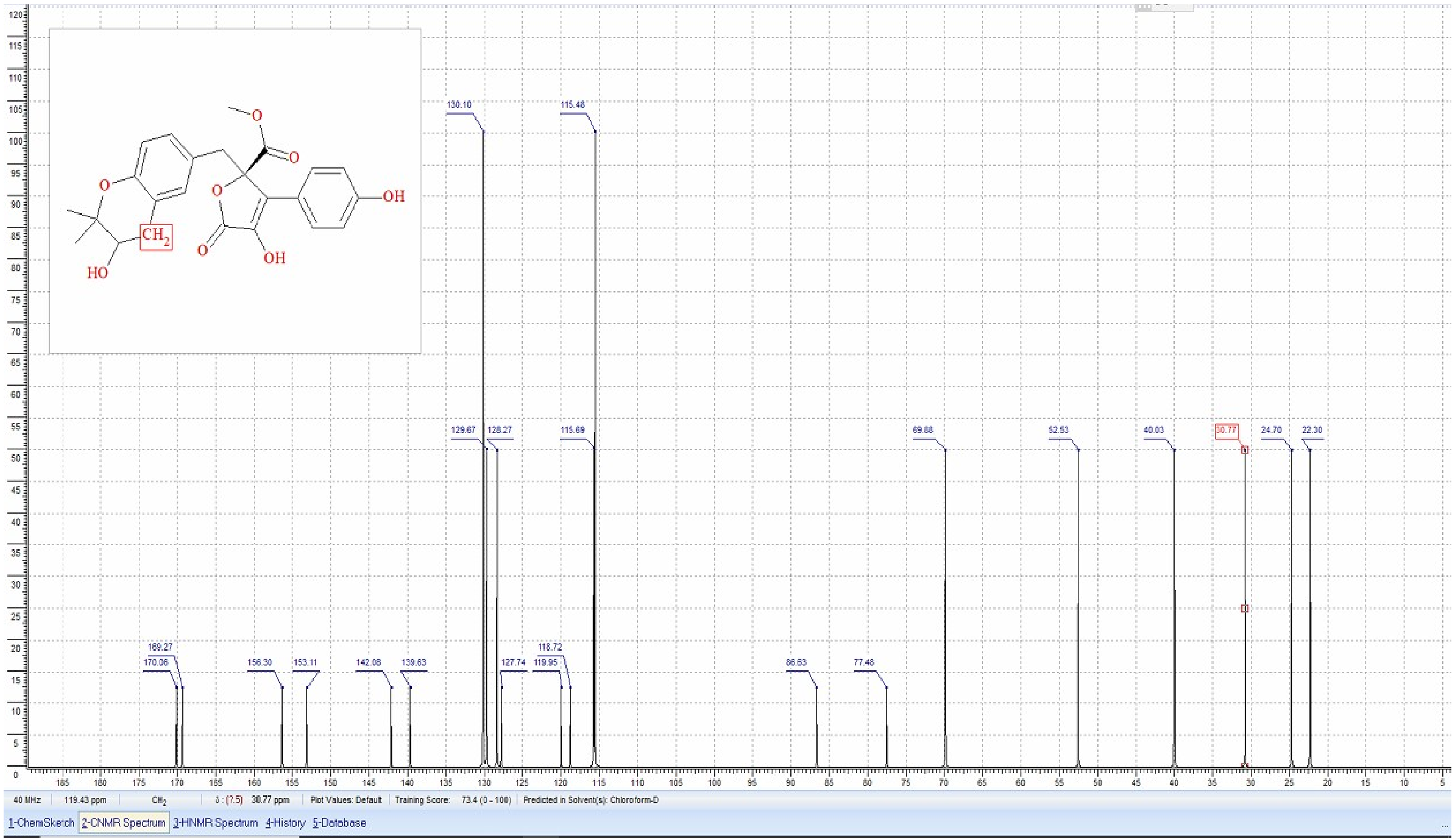

Figure S55. ^{13}C NMR prediction at C-7″ for compound **7** by ACD/Labs software

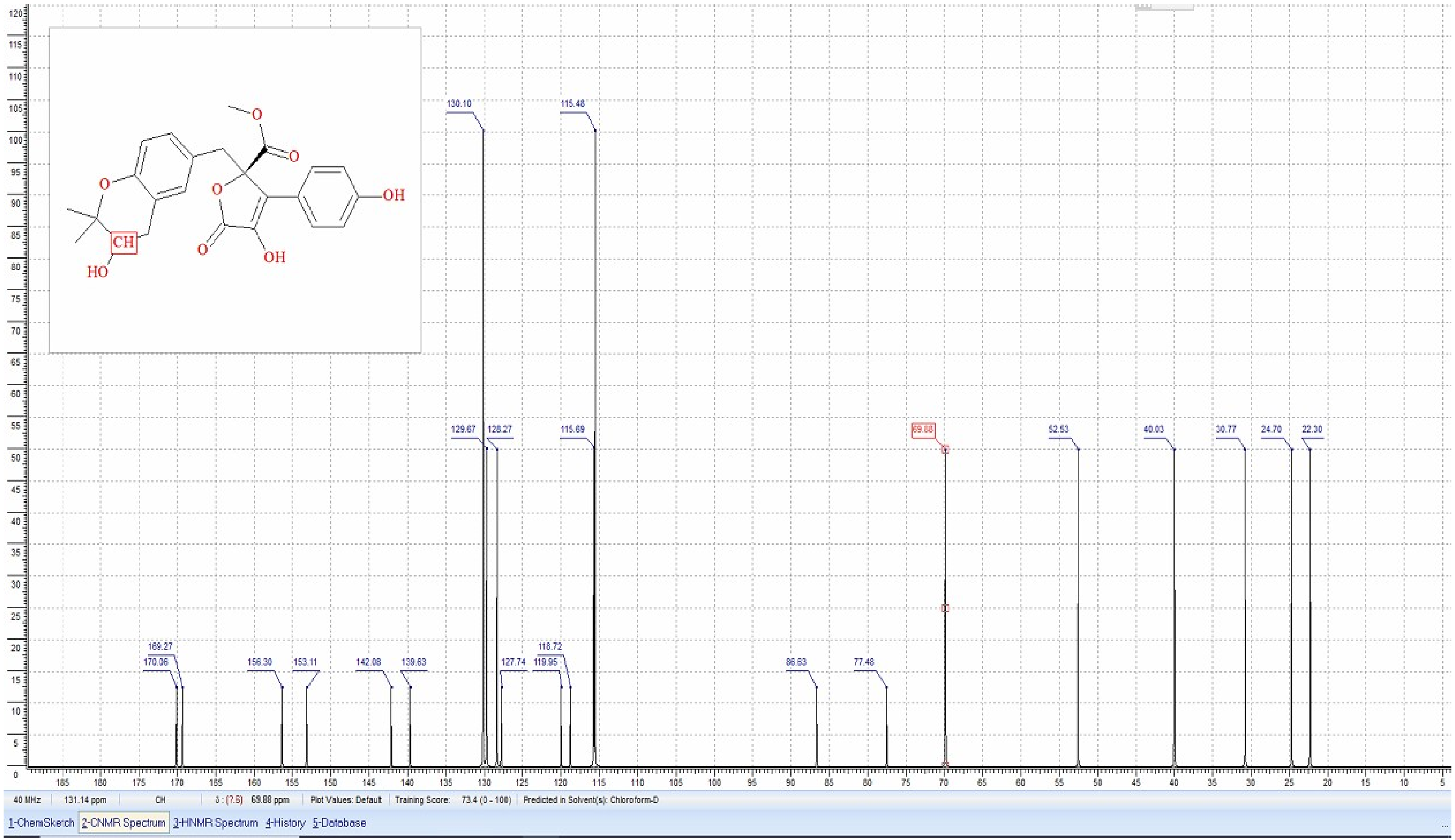

Figure S56. ^{13}C NMR prediction at C-8″ for compound **7** by ACD/Labs software

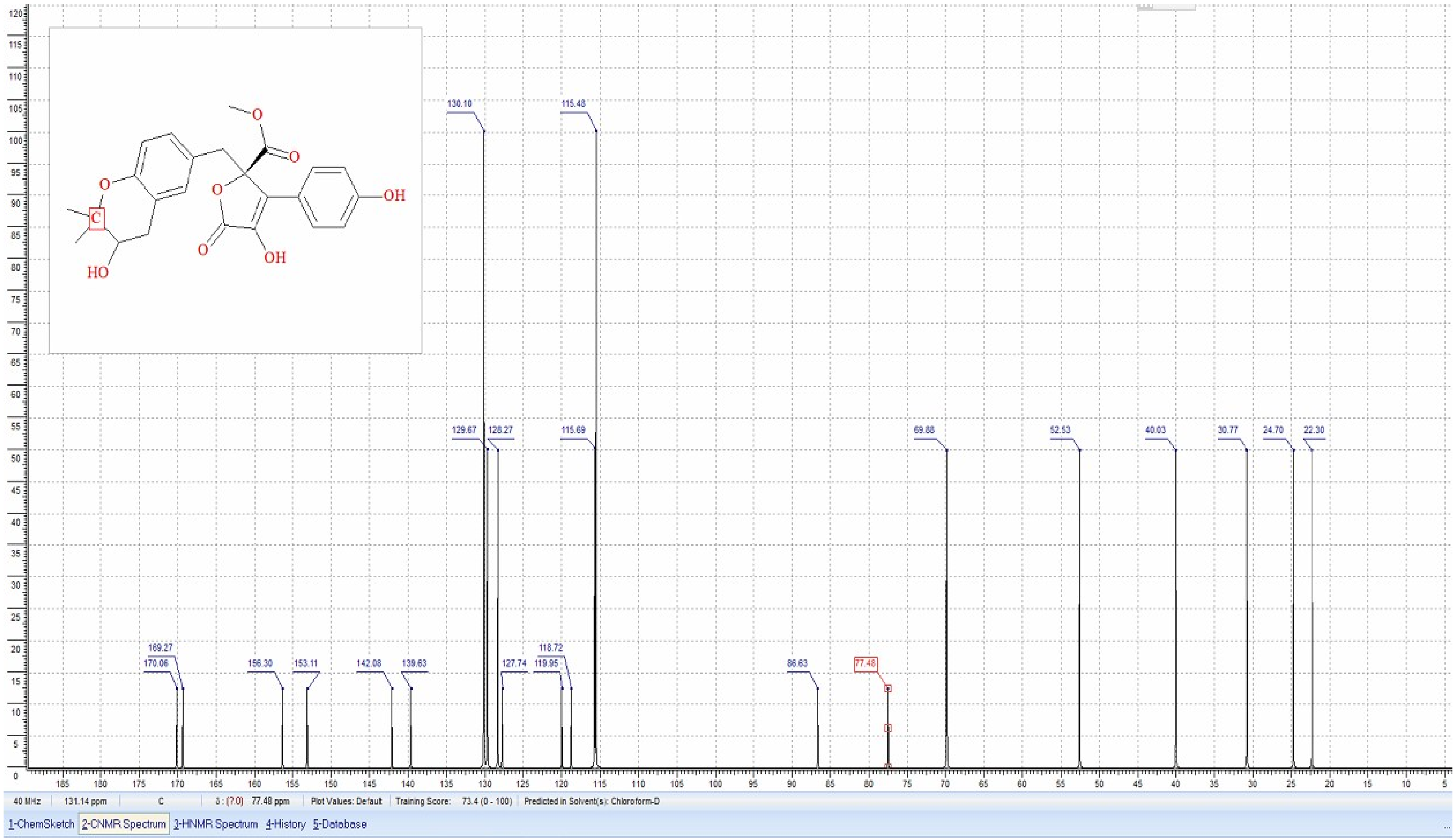

Figure S57. ^{13}C NMR prediction at C-9″ for compound **7** by ACD/Labs software

^{13}C NMR calculation data

Table S1. Important thermodynamic parameters (a.u.) and Boltzmann distributions of the optimized compound **5** at B3LYP/6-31G(d) level in chloroform.

Conformation	Energy	%
**OME107**	0	45.38%
**OME147**	0.0628	40.82%
**BAL81**	1.255	5.46%
**BAL31**	1.2613	5.40%
**BAL90**	2.2151	1.08%
**BAL64**	2.4347	0.74%
**BAL74**	2.5289	0.64%
**BAL46**	2.8238	0.39%
**BAL60**	3.6898	0.09%
**BAL82**	4.7502	0.01%

Table S2. Optimized coordinates (Å) of compound **5** in the chloroform solution at B3LYP/6-311++G** level.

**OME107**				**OME147**			
C1	-2.648	2.087	1.316	C1	-5.093	0.171	-1.284
C2	-3.629	0.949	-0.57	C2	-3.919	-1.461	0.05
C3	0.403	0.935	-1.516	C3	1.181	-1.691	0.672

C4	1.633	1.597	-1.452	C4	2.524	-1.866	1.025
C5	-3.424	3.206	1.051	C5	-6.117	-0.737	-1.506
C6	-4.412	2.067	-0.841	C6	-4.946	-2.375	-0.167
C7	1.476	-1.21	-1.197	C7	1.738	0.204	-0.726
C8	-2.727	0.926	0.512	C8	-3.962	-0.165	-0.503
C9	2.705	-0.568	-1.131	C9	3.073	0.046	-0.383
C10	0.301	-0.458	-1.387	C10	0.768	-0.665	-0.19
C11	2.769	0.82	-1.256	C11	3.45	-0.981	0.484
C12	-4.313	3.205	-0.032	C12	-6.05	-2.021	-0.949
C13	-1.898	-0.239	0.818	C13	-2.894	0.819	-0.309
C14	-1.161	-0.489	1.925	C14	-2.899	2.138	-0.622
C15	-0.486	-1.784	1.818	C15	-1.604	2.75	-0.325
C16	-3.011	-2.278	-0.189	C16	-1.564	0.161	1.753
C17	4.108	-1.095	-0.944	C17	4.322	0.772	-0.822
C18	4.961	0.205	-0.957	C18	5.41	0.138	0.089
C19	-1.699	-1.451	-0.101	C19	-1.487	0.533	0.253
C20	4.9	0.614	1.57	C20	7.608	-1.093	0.325
C21	6.643	1.737	0.131	C21	7.43	0.94	-1.167
C22	-4.746	-3.062	-1.583	C22	-1.814	0.987	3.944
C23	-1.047	-1.145	-1.472	C23	-0.692	-0.508	-0.571
C24	5.782	0.481	0.322	C24	6.7	-0.3	-0.626
O1	0.239	-2.299	2.645	O1	-1.292	3.911	-0.497

O2	-3.509	-2.819	0.771	O2	-1.569	-0.982	2.163
O3	4.04	1.317	-1.176	O3	4.791	-1.018	0.732
O4	-0.795	-2.334	0.618	O4	-0.778	1.795	0.163
O5	-5.05	4.331	-0.245	O5	-7.082	-2.873	-1.202
O6	-0.985	0.246	3.041	O6	-3.873	2.908	-1.15
O7	6.624	-0.681	0.421	O7	6.282	-1.139	-1.714
O8	-3.516	-2.317	-1.431	O8	-1.677	1.24	2.528
H1	-1.965	2.11	2.157	H1	-5.164	1.156	-1.728
H2	-3.744	0.082	-1.211	H2	-3.09	-1.762	0.677
H3	-0.5	1.521	-1.672	H3	0.436	-2.364	1.086
H4	1.703	2.676	-1.555	H4	2.835	-2.663	1.693
H5	-3.352	4.093	1.673	H5	-6.977	-0.469	-2.112
H6	-5.102	2.055	-1.681	H6	-4.888	-3.367	0.277
H7	1.415	-2.291	-1.102	H7	1.436	0.997	-1.405
H8	4.235	-1.651	-0.011	H8	4.541	0.58	-1.878
H9	4.409	-1.767	-1.755	H9	4.262	1.856	-0.681
H10	5.656	0.205	-1.803	H10	5.687	0.827	0.897
H11	5.53	0.772	2.455	H11	8.524	-1.395	-0.197
H12	4.305	-0.288	1.74	H12	7.094	-1.989	0.679
H13	4.224	1.47	1.485	H13	7.901	-0.49	1.192
H14	7.261	1.915	1.02	H14	8.346	0.635	-1.686
H15	7.309	1.614	-0.73	H15	6.804	1.488	-1.878

H16	6.02	2.621	-0.032	H16	7.718	1.621	-0.358
H17	-5.003	-2.981	-2.638	H17	-1.887	1.97	4.405
H18	-5.528	-2.628	-0.957	H18	-0.939	0.449	4.315
H19	-4.588	-4.105	-1.302	H19	-2.715	0.4	4.136
H20	-1.743	-0.535	-2.052	H20	-0.776	-0.188	-1.616
H21	-0.953	-2.099	-2.001	H21	-1.209	-1.467	-0.491
H22	-5.624	4.201	-1.018	H22	-6.912	-3.725	-0.768
H23	-0.394	-0.271	3.624	H23	-3.498	3.809	-1.229
H24	7.173	-0.573	1.214	H24	7.082	-1.421	-2.185
**BAL81**				**OME31**			
C1	-3.947	-1.276	-1.283	C1	-4.964	-0.478	0.652
C2	-5.215	0.253	0.083	C2	-3.806	-1.358	-1.271
C3	1.196	-1.746	0.441	C3	1.437	1.44	-1.637
C4	2.528	-1.938	0.83	C4	2.828	1.577	-1.594
C5	-5.064	-2.091	-1.413	C5	-5.863	-1.538	0.609
C6	-6.342	-0.556	-0.044	C6	-4.7	-2.423	-1.321
C7	1.798	0.21	-0.855	C7	1.567	-0.773	-0.663
C8	-3.993	-0.087	-0.53	C8	-3.913	-0.365	-0.282
C9	3.121	0.033	-0.478	C9	2.949	-0.653	-0.615
C10	0.813	-0.682	-0.389	C10	0.791	0.281	-1.182
C11	3.467	-1.031	0.358	C11	3.559	0.513	-1.078
C12	-6.27	-1.735	-0.793	C12	-5.732	-2.52	-0.379

C13	-2.845	0.827	-0.391	C13	-3.011	0.801	-0.212
C14	-2.873	2.163	-0.575	C14	-3.395	2.092	-0.146
C15	-1.559	2.757	-0.256	C15	-2.217	2.973	0.002
C16	-1.57	-0.085	1.534	C16	-1.146	0.046	1.239
C17	4.39	0.763	-0.85	C17	4.031	-1.563	-0.082
C18	5.412	0.165	0.148	C18	5.316	-0.882	-0.613
C19	-1.437	0.476	0.103	C19	-1.486	0.781	-0.074
C20	7.467	1.044	-1.027	C20	6.921	-2.174	0.845
C21	7.685	-0.814	0.665	C21	7.675	-0.04	-0.268
C22	-1.629	0.407	3.833	C22	-0.807	0.241	3.559
C23	-0.638	-0.503	-0.792	C23	-0.719	0.156	-1.264
C24	6.798	-0.193	-0.426	C24	6.494	-0.782	0.376
O1	-1.199	3.908	-0.308	O1	-2.159	4.176	0.081
O2	-1.824	-1.252	1.749	O2	-1.043	-1.162	1.297
O3	4.807	-1.079	0.639	O3	4.924	0.486	-0.971
O4	-0.738	1.735	0.128	O4	-1.113	2.169	0.027
O5	-7.329	-2.575	-0.957	O5	-6.634	-3.54	-0.376
O6	-3.854	2.984	-0.999	O6	-4.612	2.665	-0.21
O7	6.628	-1.124	-1.503	O7	6.057	-0.079	1.548
O8	-1.447	0.855	2.473	O8	-1.055	0.871	2.284
H1	-3.034	-1.561	-1.793	H1	-5.058	0.263	1.442
H2	-5.277	1.149	0.695	H2	-3.031	-1.293	-2.027

H3	0.438	-2.436	0.8	H3	0.842	2.254	-2.04
H4	2.817	-2.762	1.474	H4	3.321	2.473	-1.956
H5	-5.023	-3.004	-1.998	H5	-6.662	-1.623	1.339
H6	-7.271	-0.276	0.448	H6	-4.601	-3.178	-2.098
H7	1.516	1.033	-1.506	H7	1.078	-1.673	-0.3
H8	4.683	0.534	-1.882	H8	4.028	-1.573	1.015
H9	4.317	1.85	-0.75	H9	3.947	-2.597	-0.431
H10	5.531	0.822	1.018	H10	5.653	-1.364	-1.54
H11	7.612	1.819	-0.266	H11	7.255	-2.786	0.001
H12	8.448	0.771	-1.429	H12	7.752	-2.083	1.552
H13	6.87	1.461	-1.843	H13	6.101	-2.692	1.351
H14	7.217	-1.709	1.087	H14	7.378	0.965	-0.585
H15	7.863	-0.107	1.484	H15	8.05	-0.576	-1.148
H16	8.651	-1.096	0.235	H16	8.491	0.051	0.456
H17	-1.494	1.295	4.449	H17	-0.76	1.057	4.279
H18	-2.632	-0.006	3.964	H18	-1.618	-0.447	3.806
H19	-0.886	-0.353	4.082	H19	0.139	-0.304	3.532
H20	-0.705	-0.12	-1.816	H20	-1.093	0.646	-2.169
H21	-1.151	-1.466	-0.759	H21	-1	-0.898	-1.313
H22	-8.098	-2.226	-0.476	H22	-6.425	-4.161	-1.093
H23	-4.633	2.447	-1.233	H23	-5.278	1.97	-0.36
H24	6.124	-1.87	-1.134	H24	5.709	0.774	1.234

OME90							
C1	-5.111	0.165	0.252	O5	-7.19	-2.473	-1.143
C2	-3.862	-0.936	-1.492	O6	-3.957	3.158	-0.556
C3	1.222	-1.709	0.111	O7	7.456	-1.233	0.079
C4	2.533	-2.013	0.498	O8	-1.762	-1.268	1.513
C5	-6.191	-0.681	0.026	H1	-5.178	0.91	1.04
C6	-4.937	-1.789	-1.724	H2	-2.977	-1.032	-2.112
C7	1.86	0.548	-0.487	H3	0.459	-2.478	0.191
C8	-3.921	0.054	-0.496	H4	2.8	-2.996	0.871
C9	3.162	0.264	-0.104	H5	-7.1	-0.596	0.613
C10	0.866	-0.444	-0.38	H6	-4.871	-2.545	-2.503
C11	3.483	-1.006	0.38	H7	1.6	1.533	-0.866
C12	-6.107	-1.667	-0.964	H8	4.652	1.51	-1.088
C13	-2.819	1.004	-0.245	H9	4.326	1.966	0.579
C14	-2.924	2.348	-0.256	H10	5.94	0.444	1.35
C15	-1.632	2.969	0.109	H11	7.032	-1.036	-2.521
C16	-1.458	0.035	1.604	H12	5.533	-1.633	-1.785
C17	4.415	1.109	-0.097	H13	5.595	0.002	-2.472
C18	5.497	0.113	0.407	H14	7.934	1.335	0.251
C19	-1.391	0.671	0.197	H15	7.054	1.818	-1.218
C20	6.166	-0.749	-1.915	H16	8.438	0.715	-1.332
C21	7.571	0.99	-0.723	H17	-2.185	-2.986	2.501

C22	-1.92	-1.964	2.768	H18	-0.984	-1.942	3.331
C23	-0.562	-0.165	-0.807	H19	-2.714	-1.501	3.359
C24	6.656	-0.224	-0.557	H20	-0.582	0.386	-1.755
O1	-1.33	4.135	0.182	H21	-1.085	-1.11	-0.966
O2	-1.313	0.639	2.642	H22	-7.004	-3.115	-1.848
O3	4.801	-1.143	0.714	H23	-4.719	2.609	-0.816
O4	-0.751	1.953	0.349	H24	6.843	-1.952	0.308

Table S3. Important thermodynamic parameters (a.u.) and Boltzmann distributions of the optimized compound **7** at B3LYP/6-31G(d) level in chloroform.

Conformation	Energy	%
**OME39**	0	38.70%
**OME24**	0.0251	37.09%
**OME12**	0.9977	7.18%
**OME28**	1.2801	4.46%
**OME17**	1.3052	4.27%
**OME11**	1.3303	4.10%
**OME19**	1.4558	3.32%
**OME3**	2.5853	0.49%
**OME54**	2.9116	0.28%
**BAL58**	3.7776	0.07%
**BAL33**	4.129	0.04%

Table S4. Optimized coordinates (Å) of compound **7** in the chloroform solution at B3LYP/6-311++G** level.

**OME39**				**OME24**			
C1	-2.768	1.974	1.316	C1	-2.674	1.74	-0.851
C2	-3.44	1.149	-0.849	C2	-1.746	2.197	1.329
C3	0.694	1.132	-1.14	C3	1.147	-2.32	-0.935
C4	1.912	1.703	-0.787	C4	2.482	-2.277	-0.549
C5	-3.476	3.145	1.097	C5	-2.853	3.101	-1.065

C6	-4.155	2.322	-1.075	C6	-1.92	3.559	1.119
C7	1.671	-1.056	-0.963	C7	1.11	0.079	-1.061
C8	-2.726	0.942	0.349	C8	-2.116	1.249	0.35
C9	2.909	-0.51	-0.604	C9	2.452	0.154	-0.672
C10	0.55	-0.262	-1.228	C10	0.435	-1.139	-1.197
C11	3.016	0.887	-0.516	C11	3.135	-1.046	-0.417
C12	-4.177	3.328	-0.104	C12	-2.476	4.021	-0.081
C13	-1.97	-0.281	0.612	C13	-1.92	-0.179	0.599
C14	-1.408	-0.701	1.77	C14	-1.596	-0.803	1.755
C15	-0.745	-1.993	1.587	C15	-1.507	-2.25	1.556
C16	-2.912	-2.076	-0.942	C16	-3.469	-1.426	-1.006
C17	4.113	-1.383	-0.321	C17	3.168	1.48	-0.524
C18	5.414	-0.569	-0.356	C18	4.465	1.327	0.284
C19	-1.638	-1.358	-0.428	C19	-2.026	-1.283	-0.458
C20	5.25	0.753	0.43	C20	5.268	0.099	-0.205
C21	6.496	1.63	0.329	C21	6.512	-0.14	0.646
C22	4.868	0.52	1.897	C22	5.636	0.19	-1.691
C23	-4.712	-3.472	-0.336	C23	-5.694	-1.968	-0.45
C24	-0.77	-0.888	-1.62	C24	-1.016	-1.182	-1.626
O1	-0.163	-2.641	2.434	O1	-1.256	-3.089	2.398
O2	-3.316	-2.006	-2.084	O2	-3.781	-1.213	-2.159
O3	4.197	1.527	-0.223	O3	4.439	-1.085	0.013

O4	-0.881	-2.369	0.291	O4	-1.754	-2.522	0.251
O5	-4.857	4.498	-0.263	O5	-2.672	5.343	-0.343
O6	-1.394	-0.13	2.992	O6	-1.376	-0.3	2.987
O7	5.827	-0.316	-1.699	O7	4.191	1.233	1.682
O8	-3.509	-2.763	0.034	O8	-4.317	-1.8	-0.047
H1	-2.231	1.853	2.249	H1	-2.994	1.057	-1.629
H2	-3.462	0.385	-1.618	H2	-1.312	1.862	2.263
H3	-0.154	1.777	-1.35	H3	0.652	-3.281	-1.037
H4	2.03	2.781	-0.721	H4	3.039	-3.187	-0.348
H5	-3.498	3.932	1.845	H5	-3.288	3.464	-1.991
H6	-4.699	2.451	-2.007	H6	-1.622	4.268	1.888
H7	1.587	-2.137	-1.042	H7	0.578	1.008	-1.258
H8	4.007	-1.871	0.657	H8	3.395	1.903	-1.512
H9	4.189	-2.187	-1.061	H9	2.527	2.208	-0.014
H10	6.228	-1.15	0.09	H10	5.081	2.224	0.171
H11	6.306	2.603	0.793	H11	6.991	-1.08	0.354
H12	6.781	1.782	-0.714	H12	6.255	-0.186	1.706
H13	7.332	1.154	0.851	H13	7.23	0.674	0.499
H14	4.823	1.478	2.424	H14	6.248	-0.672	-1.973
H15	5.616	-0.109	2.392	H15	6.215	1.1	-1.886
H16	3.893	0.034	1.995	H16	4.752	0.201	-2.334
H17	-5.047	-3.964	0.575	H17	-6.222	-2.275	0.452

H18	-4.492	-4.206	-1.114	H18	-5.769	-2.737	-1.222
H19	-5.467	-2.769	-0.697	H19	-6.092	-1.025	-0.83
H20	-1.362	-0.187	-2.213	H20	-1.266	-0.294	-2.212
H21	-0.604	-1.766	-2.253	H21	-1.198	-2.044	-2.276
H22	-5.301	4.499	-1.127	H22	-2.378	5.871	0.417
H23	-0.899	-0.739	3.576	H23	-1.181	-1.062	3.568
H24	5.202	0.328	-2.073	H24	3.782	0.364	1.835
**OME12**				**OME28**			
C1	-3.494	0.96	-0.768	C1	-3.801	-1.443	0.179
C2	-2.733	2.08	1.227	C2	-5.027	0.196	-1.098
C3	0.613	0.95	-1.201	C3	1.329	-1.766	0.482
C4	1.828	1.584	-0.965	C4	2.687	-1.936	0.731
C5	-4.234	2.081	-1.122	C5	-4.859	-2.335	0.055
C6	-3.469	3.205	0.879	C6	-6.087	-0.693	-1.227
C7	1.648	-1.181	-0.804	C7	1.831	0.075	-0.976
C8	-2.722	0.926	0.413	C8	-3.853	-0.153	-0.395
C9	2.885	-0.573	-0.564	C9	3.203	-0.071	-0.745
C10	0.499	-0.446	-1.115	C10	0.876	-0.753	-0.377
C11	2.961	0.827	-0.642	C11	3.623	-1.091	0.123
C12	-4.226	3.214	-0.3	C12	-6.01	-1.967	-0.651
C13	-1.939	-0.245	0.807	C13	-2.753	0.808	-0.291
C14	-1.34	-0.505	1.992	C14	-2.749	2.121	-0.629

C15	-0.662	-1.802	1.957	C15	-1.427	2.71	-0.422
C16	-2.931	-2.273	-0.342	C16	-1.311	0.154	1.694
C17	4.122	-1.382	-0.234	C17	4.224	0.836	-1.399
C18	5.399	-0.548	-0.411	C18	5.574	0.765	-0.674
C19	-1.636	-1.451	-0.09	C19	-1.32	0.501	0.187
C20	5.228	0.849	0.23	C20	5.966	-0.707	-0.4
C21	6.446	1.736	-0.016	C21	7.255	-0.806	0.412
C22	4.902	0.776	1.726	C22	6.069	-1.534	-1.687
C23	-4.484	-3.045	-1.942	C23	-1.433	1.018	3.881
C24	-0.823	-1.139	-1.371	C24	-0.601	-0.57	-0.669
O1	-0.043	-2.325	2.861	O1	-1.102	3.861	-0.634
O2	-3.543	-2.817	0.548	O2	-1.294	-0.982	2.123
O3	4.135	1.521	-0.469	O3	4.94	-1.289	0.461
O4	-0.827	-2.342	0.725	O4	-0.592	1.745	0.033
O5	-4.968	4.285	-0.696	O5	-7.015	-2.882	-0.741
O6	-1.296	0.223	3.126	O6	-3.738	2.902	-1.112
O7	5.753	-0.435	-1.79	O7	5.55	1.522	0.536
O8	-3.281	-2.305	-1.635	O8	-1.377	1.246	2.456
H1	-3.535	0.097	-1.422	H1	-2.935	-1.753	0.75
H2	-2.153	2.096	2.142	H2	-5.109	1.175	-1.554
H3	-0.258	1.549	-1.454	H3	0.611	-2.423	0.963
H4	1.922	2.663	-1.03	H4	3.043	-2.719	1.393

H5	-4.826	2.092	-2.032	H5	-4.807	-3.322	0.505
H6	-3.457	4.081	1.523	H6	-6.977	-0.398	-1.779
H7	1.586	-2.266	-0.75	H7	1.502	0.87	-1.643
H8	4.066	-1.758	0.796	H8	4.356	0.563	-2.455
H9	4.191	-2.262	-0.882	H9	3.878	1.876	-1.388
H10	6.244	-1.057	0.062	H10	6.352	1.23	-1.287
H11	6.249	2.752	0.342	H11	7.446	-1.849	0.684
H12	6.69	1.774	-1.08	H12	7.186	-0.207	1.323
H13	7.313	1.34	0.524	H13	8.101	-0.442	-0.18
H14	4.842	1.787	2.141	H14	6.409	-2.547	-1.45
H15	5.687	0.231	2.262	H15	6.792	-1.083	-2.377
H16	3.948	0.276	1.915	H16	5.108	-1.612	-2.202
H17	-4.604	-2.966	-3.021	H17	-1.475	2.01	4.329
H18	-5.338	-2.605	-1.423	H18	-0.542	0.481	4.212
H19	-4.368	-4.088	-1.639	H19	-2.325	0.44	4.135
H20	-1.444	-0.528	-2.03	H20	-0.741	-0.261	-1.711
H21	-0.66	-2.091	-1.886	H21	-1.132	-1.516	-0.544
H22	-4.882	4.997	-0.041	H22	-7.752	-2.504	-1.249
H23	-0.779	-0.3	3.771	H23	-3.351	3.793	-1.23
H24	5.1	0.155	-2.205	H24	4.954	1.062	1.151
**OME17**				**OME11**			
C1	-3.321	-1.69	0.269	C1	-2.761	1.616	-0.716

C2	-5.081	-0.393	-0.752	C2	-1.755	2.199	1.397
C3	1.575	1.16	-1.529	C3	1.143	-2.361	-0.81
C4	2.958	1.278	-1.466	C4	2.494	-2.269	-0.494
C5	-4.138	-2.813	0.258	C5	-2.981	2.96	-0.988
C6	-5.902	-1.514	-0.767	C6	-1.97	3.545	1.13
C7	1.69	-0.865	-0.243	C7	1.032	0.03	-1.021
C8	-3.766	-0.449	-0.237	C8	-2.141	1.196	0.481
C9	3.085	-0.775	-0.165	C9	2.389	0.155	-0.703
C10	0.914	0.087	-0.91	C10	0.387	-1.209	-1.079
C11	3.714	0.316	-0.785	C11	3.117	-1.016	-0.44
C12	-5.435	-2.734	-0.262	C12	-2.586	3.936	-0.066
C13	-2.927	0.751	-0.251	C13	-1.9	-0.213	0.789
C14	-3.29	2.027	-0.529	C14	-1.529	-0.773	1.964
C15	-2.132	2.92	-0.491	C15	-1.399	-2.225	1.83
C16	-1.084	0.454	1.473	C16	-3.521	-1.609	-0.565
C17	3.913	-1.819	0.555	C17	3.074	1.505	-0.636
C18	5.389	-1.736	0.145	C18	4.411	1.414	0.114
C19	-1.407	0.799	0	C19	-2.028	-1.373	-0.206
C20	5.875	-0.267	0.162	C20	5.224	0.193	-0.375
C21	7.313	-0.142	-0.336	C21	6.516	0.019	0.42
C22	5.719	0.388	1.54	C22	5.515	0.242	-1.879
C23	-1.071	1.248	3.689	C23	-5.159	-1.58	-2.263

C24	-0.593	-0.057	-0.999	C24	-1.083	-1.307	-1.431
O1	-2.128	4.117	-0.697	O1	-1.092	-3.013	2.701
O2	-0.729	-0.647	1.844	O2	-4.344	-1.951	0.253
O3	5.079	0.473	-0.814	O3	4.443	-1.005	-0.075
O4	-1.031	2.185	-0.204	O4	-1.671	-2.565	0.546
O5	-6.191	-3.867	-0.249	O5	-2.824	5.239	-0.385
O6	-4.498	2.548	-0.829	O6	-1.286	-0.205	3.162
O7	5.602	-2.328	-1.137	O7	4.207	1.36	1.526
O8	-1.293	1.491	2.283	O8	-3.783	-1.391	-1.861
H1	-2.331	-1.782	0.698	H1	-3.094	0.888	-1.446
H2	-5.462	0.538	-1.151	H2	-1.276	1.919	2.327
H3	1.001	1.912	-2.063	H3	0.67	-3.338	-0.851
H4	3.475	2.105	-1.944	H4	3.086	-3.155	-0.288
H5	-3.785	-3.76	0.654	H5	-3.463	3.269	-1.91
H6	-6.908	-1.443	-1.174	H6	-1.658	4.296	1.853
H7	1.199	-1.709	0.235	H7	0.466	0.937	-1.225
H8	3.823	-1.694	1.643	H8	3.242	1.898	-1.647
H9	3.546	-2.826	0.328	H9	2.437	2.233	-0.121
H10	6.002	-2.321	0.837	H10	4.996	2.323	-0.058
H11	7.589	0.914	-0.42	H11	7.008	-0.916	0.136
H12	7.431	-0.621	-1.31	H12	6.313	0.003	1.493
H13	7.997	-0.623	0.371	H13	7.2	0.848	0.209

H14	6.133	1.401	1.515	H14	6.143	-0.608	-2.162
H15	6.26	-0.186	2.3	H15	6.048	1.164	-2.137
H16	4.671	0.46	1.844	H16	4.599	0.198	-2.476
H17	-1.286	2.195	4.182	H17	-5.181	-1.357	-3.329
H18	-0.035	0.949	3.86	H18	-5.809	-0.898	-1.71
H19	-1.743	0.464	4.046	H19	-5.465	-2.611	-2.076
H20	-0.942	0.238	-1.995	H20	-1.38	-0.454	-2.046
H21	-0.879	-1.102	-0.861	H21	-1.271	-2.205	-2.028
H22	-7.063	-3.678	-0.635	H22	-2.512	5.811	0.336
H23	-4.359	3.506	-0.964	H23	-1.054	-0.933	3.772
H24	5.195	-1.739	-1.794	H24	3.832	0.486	1.727
**OME19**							
C1	-2.876	1.951	1.39	O5	-5.108	4.287	-0.281
C2	-3.57	0.966	-0.699	O6	-1.406	-0.01	3.16
C3	0.552	1.095	-1.058	O7	6.435	-1.004	0.467
C4	1.754	1.732	-0.771	O8	-3.253	-2.215	-1.814
C5	-3.639	3.077	1.119	H1	-2.31	1.91	2.313
C6	-4.339	2.093	-0.977	H2	-3.577	0.158	-1.42
C7	1.63	-1.038	-0.831	H3	-0.331	1.695	-1.261
C8	-2.817	0.863	0.488	H4	1.824	2.816	-0.746
C9	2.853	-0.425	-0.537	H5	-3.672	3.907	1.818
C10	0.468	-0.305	-1.094	H6	-4.913	2.143	-1.899

C11	2.905	0.978	-0.51	H7	1.589	-2.125	-0.849
C12	-4.378	3.157	-0.069	H8	3.859	-2.1	0.369
C13	-2.006	-0.312	0.802	H9	4.536	-1.619	-1.182
C14	-1.415	-0.647	1.972	H10	4.824	-0.161	1.48
C15	-0.699	-1.919	1.847	H11	5.928	1.86	-2.139
C16	-2.923	-2.271	-0.515	H12	5.019	0.351	-2.316
C17	4.102	-1.228	-0.252	H13	6.706	0.303	-1.769
C18	5.158	-0.37	0.451	H14	6.306	2.895	0.083
C19	-1.657	-1.436	-0.181	H15	7.277	1.476	0.543
C20	5.324	0.996	-0.249	H16	5.929	2.005	1.573
C21	5.77	0.866	-1.71	H17	-4.535	-2.807	-3.268
C22	6.27	1.9	0.538	H18	-5.303	-2.593	-1.658
C23	-4.428	-2.966	-2.196	H19	-4.284	-4.025	-1.972
C24	-0.835	-1.003	-1.421	H20	-1.463	-0.355	-2.038
O1	-0.076	-2.493	2.717	H21	-0.646	-1.907	-2.009
O2	-3.533	-2.898	0.32	H22	-5.574	4.216	-1.131
O3	4.039	1.68	-0.204	H23	-0.879	-0.565	3.769
O4	-0.834	-2.366	0.575	H24	6.337	-1.844	0.943
